# Structure Revision of Formyl Phloroglucinol Meroterpenoids: A Unified Approach Using NMR Fingerprinting and DFT NMR and ECD Analyses

**DOI:** 10.3390/molecules29030594

**Published:** 2024-01-25

**Authors:** Darren C. Holland, Anthony R. Carroll

**Affiliations:** 1 School of Environment and Science, Griffith University, Nathan, QLD 4111, Australia; 2 Griffith Institute for Drug Discovery, Griffith University, Nathan, QLD 4111, Australia

**Keywords:** structure revision, formylated phloroglucinols, NMR fingerprinting, DFT, DP4+

## Abstract

NMR fingerprints are valuable tools for analyzing complex natural product mixtures and identifying incorrectly assigned structures in the literature. Our diagnostic NMR fingerprints for formyl phloroglucinol meroterpenoids revealed discrepancies in the structures reported for eucalyprobusal C (**1a**) and eucalypcamal K (**2a**). NMR fingerprinting PCA analyses identified **1a** as an oxepine-diformyl phloroglucinol and **2a** as an oxepine 3-acyl-1-formyl phloroglucinol, contrary to their initial assignments as pyrano-diformyl and pyrano 3-acyl-1-formyl phloroglucinols, respectively. Extensive reinterpretation of their reported one- and two-dimensional NMR data, coupled with GIAO DFT-calculated ^1^H and ^13^C NMR chemical shift and DP4+ analyses, supported the unequivocal reassignment of eucalyprobusal C to **1b** and eucalypcamal K to **2b**. The absolute configurations of the revised oxepine-containing phloroglucinol meroterpenoids were confirmed via the reinterpretation of their reported ROESY and NOESY NMR data, along with comparative TDDFT-calculated and experimental ECD spectra.

## 1. Introduction

Accurately establishing the correct molecular structures of complex natural products (NPs) remains crucial for their exploitation by various disciplines, such as biochemistry, drug discovery, agriculture, synthetic biology, and molecular biology. Despite advances in nuclear magnetic resonance spectroscopy (NMR) and associated resources designed to aid NP structure elucidation and dereplication [1,2,3,4], incorrectly assigned compounds continue to permeate the literature and associated databases [5,6,7,8,9,10]. While semi-synthesis or total synthesis and/or single-crystal X-ray diffraction (XRD) provide important methods for confirming the identity of NP structures, difficulties encountered with both approaches, such as synthesizing complex NP scaffolds and inherent complications in crystallization, make computational methods more appealing and cost-effective. Recently, we illustrated the power of principal component analyses (PCA) and machine learning-generated NMR fingerprints for identifying common subclasses of formyl phloroglucinol meroterpenoids (FPCs) in complex NP mixtures [7]. This resulted in the targeted extract selection of *Eucalyptus gittinsii* subsp. *gittinsii* and the subsequent isolation and identification of three pyrano-acyl-formyl phloroglucinol NPs [7]. Moreover, utilizing our diagnostic phloroglucinol NMR fingerprint method, 167 inaccurately reported chemical shifts for 44 phloroglucinol-containing NPs were reassigned [7]. In addition, the structures of three erroneously reported NPs—euglobal In-1, psiguadiol E, and psiguadiol G—were revised, corrections of which were validated using gauge, including atomic orbital (GIAO) density functional theory (DFT) NMR calculations [7].

The genus *Eucalyptus* resides within the Myrtaceae family of flowering plants and comprises more than 800 species, of which >99% are endemic to Australia. The diversity of *Eucalyptus* is primarily distributed throughout three subgroups: *Eucalyptus* (717 species), *Corymbia* (90 species), and *Angophora* (12 species) [11]. At the chemotaxonomic level, formyl phloroglucinol meroterpenoids (FPCs) isolated from *Eucalyptus* contrast with the chemical profiles of sister genera *Corymbia* and *Angophora*, both of which contain tetramethyl β-triketone acyl phloroglucinol derivatives [12,13,14,15,16,17]. These findings support Hill and Johnson’s (1995) morphological and phylogenetic taxonomic separation of *Corymbia* (and close taxonomic relationship with *Angophora*) [18], despite its formal reclassification as a sub-genera of *Eucalyptus* by Brooker (2000) [19]. Therefore, the presence or absence of FPCs in Eucalypt extracts offers interesting chemotaxonomic data that contribute to an ongoing taxonomic debate within the speciose genus *Eucalyptus* and the closely related Eucalypt genera, *Corymbia* and *Angophora*. The remarkable chemical diversity of *Eucalyptus*-derived phloroglucinol NPs, encompassing monomers, dimers, trimers, oligomers, polycyclics, meroterpenoids, xanthones, flavonoids, and coumarins, coupled with their demonstrated bioactivities against a broad range of diseases and infection targets, make them attractive targets for biodiscovery efforts [7,20]. Of particular note are the bioactivities demonstrated by FPCs against the pharmacologically relevant infective-disease-causing targets *Staphylococcus aureus* [21] and *Plasmodium falciparum* [22]. 

Machine/deep learning, a subset of artificial intelligence (AI), employs computational algorithms that can be trained to analyze large and high-dimensional datasets without the need for explicit programming. However, the accuracy of machine/deep learning output analyses relies heavily on the precision of the input data under examination [23]. Unfortunately, with incorrectly assigned NP structures continuing to pollute the literature and associated large NP databases, the accuracy of AI-based computational analyses for aiding NP structure elucidation will likely be compromised. While less computationally expensive NMR fact-checking methods are emerging [3,24], solution state GIAO DFT calculations remain best practice for accurately assessing the connectivity and configuration of NP structures [25,26,27]. In addition, comparative metrics commonly employed to compare the accuracy of DFT NMR-calculated chemical shifts with experimental ones, specifically MAE and RMSD, can be expanded upon with DP4+ Bayesian theorem algorithms [28,29]. These probabilistic algorithms analyze and compare scaled and unscaled ^1^H and ^13^C chemical shifts with experimental NMR data, facilitating the resolution of multiple candidate structures for a given NP.

**Figure 1:**
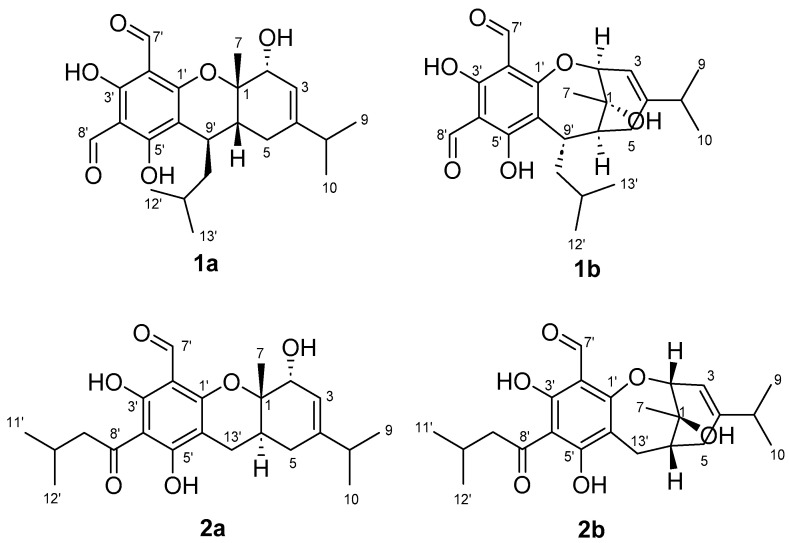
Incorrectly assigned pyrano formyl phloroglucinol structures reported for eucalyprobusal C (**1a**) and eucalypcamal K (**2a**) and their revised oxepine formyl phloroglucinol structures **1b** and **2b**.

Herein, we present a comprehensive approach employing diagnostic NMR fingerprints of FPCs and GIAO DFT NMR analyses. This unified strategy, alongside the reinterpretation of one- and two-dimensional NMR data and comparative time-dependent functional theory (TDDFT) ECD analyses, allowed for the identification and the reassignment of the planar and three-dimensional structures of two misassigned NPs, eucalyprobusal C (**1a**) [30] and eucalypcamal K (**2a**) [31], to oxepine FPCs **1b** and **2b**, respectively (Figure 1).

## 2. Results

### 2.1. Formyl Phloroglucinol NMR Fingerprinting and PCA Analysis

**Figure 2:**
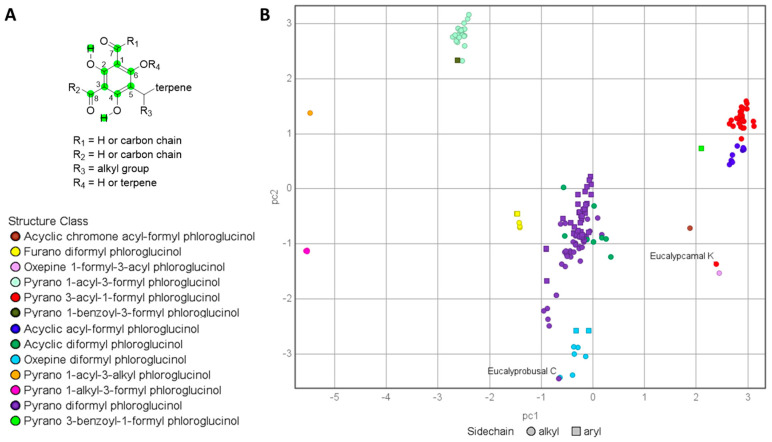
(**A**): NMR chemical shifts analyzed via PCA (green = carbon and hydrogen chemical shifts analyzed). (**B**): PCA analysis of ^1^H and ^13^C NMR data for formyl phloroglucinols (*n* = 179) color coded by sub-structure class. The published structures for eucalyprobusal C and eucalypcamal K (**1a** and **2a**, annotated) do not cluster with other members of their assigned formyl phloroglucinol class, indicative of their structural misassignments.

In a recent publication, we reported the first FPCs containing two spatially separated formyl phloroglucinols conjugated to a terpene core from *Eucalyptus camaldulensis* [32]. As part of this study, we investigated the structure–activity relationships (SARs) associated with the antibacterial activities of related FPCs. However, during the aforementioned SAR analyses, it became clear that two recently reported FPCs with antibacterial activity, eucalyprobusal C (**1a**) and eucalypcamal K (**2a**), were assigned structures inconsistent with their reported NMR data [30,31]. To assess these inconsistencies in more detail, the NMR data (^1^H and ^13^C) assigned to the phloroglucinol cores in **1a** and **2a** were appended to the tabulated NMR data already generated for the 131 FPCs used for our previously reported FPC NMR fingerprints protocol [7]. The tabulated NMR dataset was expanded to include recently published FPCs and now consists of 179 compounds with NMR data reported in CDCl_3_. The NMR data for 179 FPC’s analyzed via PCA included the six carbons (C-1–C-6) associated with phloroglucinol, aldehyde carbonyl carbons (C-7 and C-9), and associated aldehydic and phenolic protons (Figure 2A). With the PCA output color coded according to the phloroglucinol substructure classes, it was clear that eucalyprobusal C (**1a**) and eucalypcamal K (**2a**) occupied regions of PCA space inconsistent with their proposed structure classes (Figure 2B, annotated). Instead, eucalyprobusal C (**1a**) more closely aligns with oxepine-diformyl phloroglucinols (not pyrano-diformyl phloroglucinols), while eucalypcamal K (**2a**) is a better match for an oxepine-1-formyl-3-acyl phloroglucinol (not a pyrano 3-acyl-1-formyl phloroglucinol).

The ^1^H and ^13^C NMR data reported for eucalyprobusal C (**1a**) and eucalypcamal K (**2a**) were compared with the diagnostic NMR chemical shifts ranges for subclasses of FPCs reported in the Supplementary Materials of our NMR fingerprinting protocol (adapted Figure S1) [7]. Eucalyprobusal C (**1a**) displayed markedly better alignment with the NMR fingerprint data ranges associated with oxepine-formyl phloroglucinols in contrast with pyrano-diformyl phloroglucinol NPs (Table S9). Phloroglucinol carbons C-2′ and C-6′ and formyl carbonyl carbon C-7′ in **1a** were exceptionally diagnostic and displayed large deviations from the chemical shift ranges for these positions in pyrano-diformyl phloroglucinols (^13^C = 1.0–12.5 ppm). Moreover, eucalypcamal K (**2a**) was a more suitable match with the ^1^H and ^13^C NMR phloroglucinol fingerprint data for oxepine-formyl phloroglucinols compared with its assignment as a pyrano-3-acyl-1-formyl phloroglucinol (Table S10). Carbons C-1′, 2′, 4′, 6′, and aldehyde C-7′ in **2a** contained large chemical shift deviations from the ranges associated with 3-acyl-1-formyl phloroglucinols (^13^C = 1.4–13.8 ppm). The power and utility of FPC fingerprinting is effectively demonstrated herein, with eucalyprobusal C (**1a**) and eucalypcamal K (**2a**) identified as containing structures inconsistent with their proposed structure classes. Moreover, this method contains important predictive capabilities, leading to the re-evaluation of their likely chemical structures as oxepine-diformyl and oxepine-1-formyl-3-acyl phloroglucinols, respectively.

### 2.2. Reanalysis of One- and Two-Dimensional NMR Data Reported for Eucalyprobusal C and Eucalypcamal K

**Figure 3:**
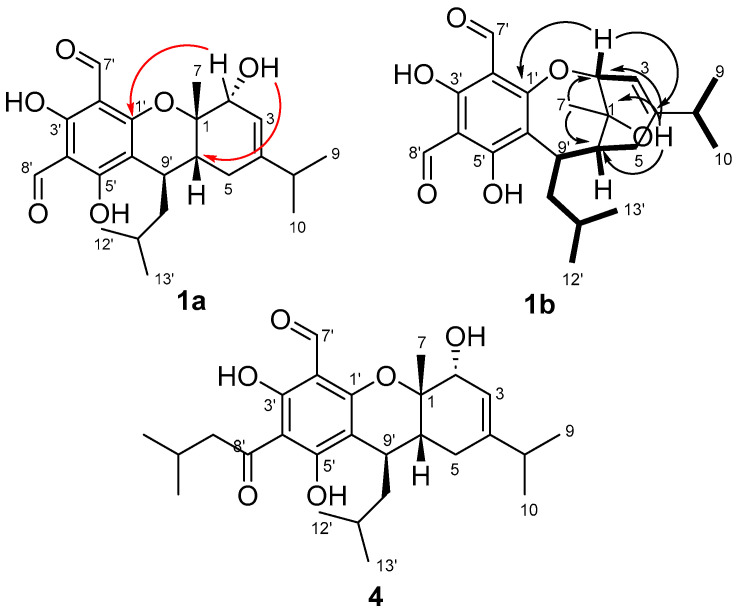
HMBC (arrows) and COSY (bolded lines) correlations for the structure reassignment of eucalyprobusal C from **1a** to **1b**. Red arrows represent ^4^*J*_CH_ HMBC correlations from 2-OH in **1a**, which are more likely ^3^*J*_CH_ correlations from 1-OH in revised **1b**. Eucalypcamal K (**2a**) was incorrectly ascribed as the C-6 diastereomer of the co-isolated pyrano 3-acyl-1-formyl phloroglucinol, eucalypcamal L (**4**).

To confirm our NMR FPC fingerprint analyses and the true structural identities of eucalyprobusal C and eucalypcamal K , their experimental NMR data were thoroughly reanalyzed and compared with the NMR data reported for related FPCs. NMR spectroscopic similarities for the terpenoid sub-structures (MAE = 1.2) of eucalyprobusal C (**1a**, C-1 to C-10 and C-9′) and eucalypcamal K (**2a**, C-1 to C-10 and C-13′) advocated for identical terpene substructures, with the exception of an alkyl-substituted methine in **1a** instead of a methylene in **2a**. In addition, eucalypcamal K (**2a**) exhibited significant chemical shift differences compared with co-isolated eucalypcamal L (**4**), a pyrano 3-acyl-1-formyl phloroglucinol and proposed diastereomer of **2a** (Figure 3) [31]. Consistent with our PCA and FPC NMR chemical shift analyses above (Figure 2B and Table S10), distinct NMR chemical shift differences between **2a** and **4** were evident for phloroglucinol carbons C-2′ (*δ*_C_ 108.4 vs. 103.8), C-4′ (*δ*_C_ 105.6 vs. 103.8), and C-6′ (*δ*_C_ 99.3 vs. 112.3), as well as the aldehyde carbonyl carbon C-7′ (*δ*_C_ 193.4 and 191.8). In addition, terpenoid carbons C-1 (*δ*_C_ 72.4 vs. 81.7), C-2 (*δ*_C_ 77.9 vs. 69.2), C-3 (*δ*_C_ 112.2 vs. 118.8), C-4 (*δ*_C_ 154.7 vs. 144.9), and C-6 (*δ*_C_ 36.7 vs. 32.8) and the methylene C-7′ (*δ*_C_ 24.1 vs. 21.2) also shared large chemical shift deviations, suggesting eucalypcamal K (**2a**) was indeed not a diastereomer of eucalypcamal L (**4**).

In addition, closer inspection of the experimental NMR data provided in the supplementary data for eucalyprobusal C (**1a**) [30] revealed an unassigned oxygenated proton resonance at *δ*_H_ 1.80 consistent with an alcohol group. The oxygenated proton resonance (2-OH in **1a**) exhibited three HMBC correlations, two of which should be expected for both structures (**1a** and **1b**) to carbon signals at *δ*_C_ 72.7 (C-1) and 80.2 (C-2). However, a third HMBC correlation was observed to *δ*_C_ 40.2 (C-6), a correlation of which is more likely a ^3^*J*_CH_ correlation in **1b** than a ^4^*J*_CH_ correlation in **1a** (Figure 3). 

Further, if **1a** was indeed a pyran-substituted FPC, a three-bond HMBC correlation would be expected from 1-OH to the *sp*^2^ methine C-3 (*δ*_C_ 111.6); however, this correlation was not observed in the reported NMR data. In addition, the HMBC data reported for both eucalyprobusal C and eucalypcamal K clearly displayed ^3^*J*_CH_ correlations from H-2 (*δ*_H_ 4.49 and 4.51, respectively) to the oxygenated phloroglucinol carbon C-1′ (*δ*_C_ 165.0 and 164.6, respectively), correlations that could only be assigned as unlikely 4-bond HMBC correlations in the pyrano FPC structures **1a** and **2a**. These findings clearly suggest that methyl-substituted C-1 in **1a** and **2a** should be reassigned from an ether to an alcohol in the revised structures **1b** and **2b**. Moreover, C-2 should also be revised from a secondary alcohol in **1a** and **2a** to a methine-forming part of an ether linkage to C-1′ of phloroglucinol in **1b** and **2b**. Reanalysis of the remaining COSY and HMBC NMR data for the terpene substructures for eucalyprobusal C and eucalypcamal K was consistent with ring expansion from a six-membered pyran system to a seven-membered oxepine in the revised structures **1b** and **2b**. The connectivity of the isopropyl groups to C-4 in both **1b** and **2b**, as well as the isobutyl to C-9′ in **1b**, were consistent with that proposed in their original structure assignments [30,31]. 

**Figure 4:**
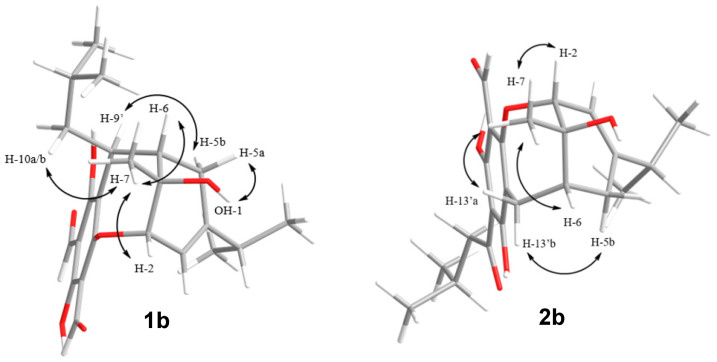
Key ROESY (eucalyprobusal C) and NOESY (eucalypcamal K) NMR correlations (arrows) used to ascribe the relative configurations for the revised structures **1b** and **2b**.

The relative configurations of the revised planar structures **1b** and **2b** were determined via thorough re-examination of the ROESY NMR spectra for eucalyprobusal C and NOESY NMR spectra for eucalypcamal K, provided in their respective supplementary information [30,31]. Key ROESY correlations from 1-OH to H-5a, as well as from methyl protons H-7 to methylene protons H-10′ and methine H-2, were consistent with *S relative configurations at stereocenters C-2, C-6, and C-7 in **1b** (Figure 4). Further, the methine proton H-9′ shared a ROESY correlation with the methylene proton H-5b, suggesting that C-9′ also shared *S relative configuration. 

Eucalypcamal K (**2b**) displayed NOESY correlations consistent with the ROESY correlations observed for **1b** (Figure 4). Key NOESY correlations from methyl protons H-7 to H-2 and alpha methylene proton H-13′a ascribed *R relative configurations at C-1 and C-2, while C-6 was also assigned *R relative configuration with shared NOESY correlations between H-13′b and beta methylene proton H-5b.

### 2.3. GIAO DFT NMR Chemical Shift Analyses for ***1a***, ***1b***, ***2a***, and ***2b*** with Experimental NMR Data for Eucalyprobusal C and Eucalypcamal K

**Figure 5:**
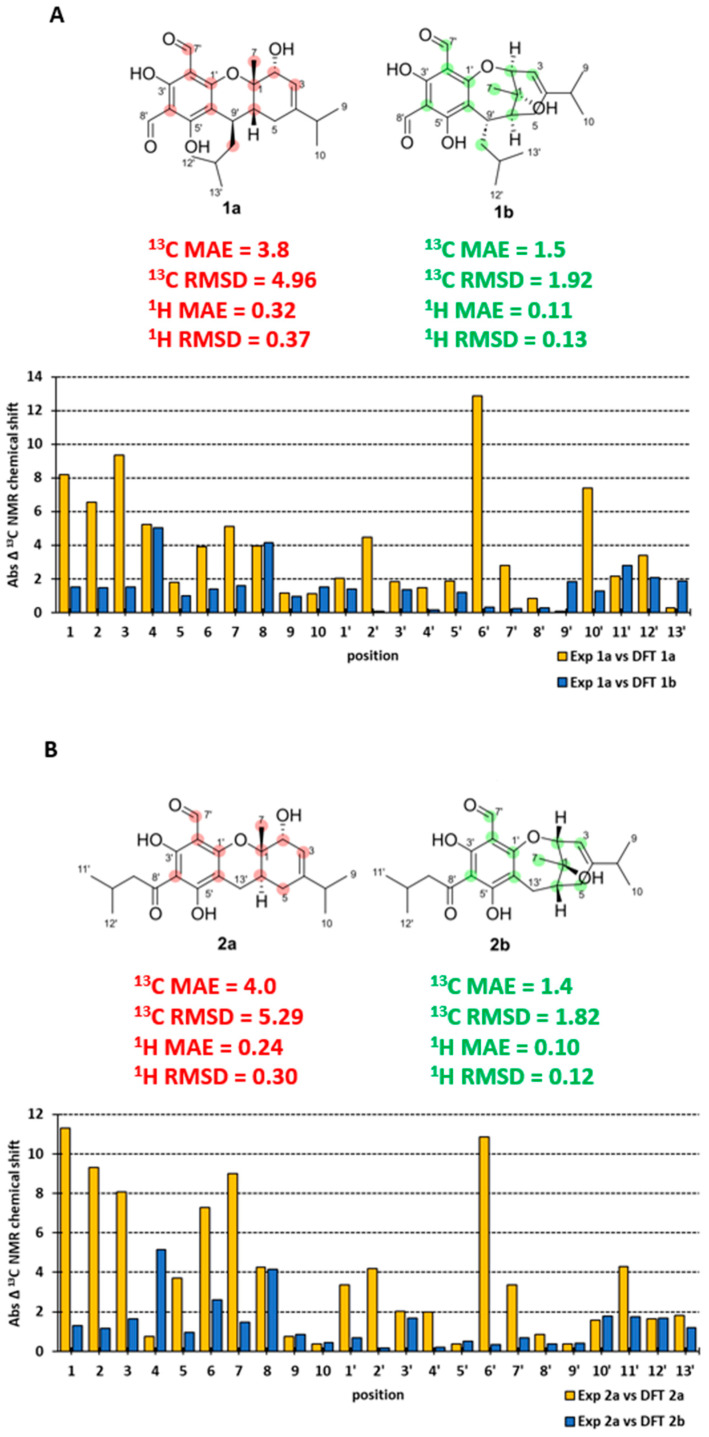
(**A**): ^13^C NMR experimental and DFT-calculated data absolute error (incorrect **1a** = yellow and revised **1b** = blue) for eucalyprobusal C. (**B**): ^13^C NMR experimental and DFT-calculated data absolute error (incorrect **2a** = yellow and revised **2b** = blue) for eucalypcamal K.

To confirm our findings from FPC NMR fingerprinting analyses and re-evaluation of the reported NMR data for eucalyprobusal C and eucalypcamal K, DFT GIAO NMR calculations were performed on the incorrectly assigned (**1a** and **2a**) and revised FPC structures (**1b** and **2b**) and compared with their reported experimental NMR data. The experimental ^13^C NMR chemical shifts were in poor agreement with the DFT-calculated NMR chemical shifts for structures **1a** (^13^C MAE = 3.8, RMSD = 4.96) and **2a** (^13^C MAE = 4.0 and RMSD = 5.29, Figure 5A,B). Notably, and consistent with our FPC NMR fingerprinting analyses outlined above, large deviations in carbon chemical shifts were observed for phloroglucinol carbons C-2′, 4′, and 6′ in **1a**, as well as C-1′, 2′ and 6′ in **2a**, alongside formyl carbonyl carbons C-7′ in both (Figure 5A,B, Tables S1 and S5). Furthermore, significant chemical shift differences were observed for the terpenoid carbons in **1a** (C-1, 2, 3, 6, 7, and 10′) and **2a** (C-1, 2, 3, 5, 6, 7, and 11′), consistent with their misassignment as pyran-substituted phloroglucinols. The DFT GIAO-calculated ^13^C chemical shifts for revised structures **1b** (^13^C MAE = 1.5, RMSD = 1.92) and **2b** (^13^C MAE = 1.4, RMSD = 1.82) were in excellent agreement with the experimental NMR data for eucalyprobusal C and eucalypcamal K (Figure 5A,B, Tables S2 and S6).

The phloroglucinol carbons (C-1′–C-6′) and the formyl carbon C-7′ for both **1b** and **2b** were excellent matches with the published experimental ^13^C NMR data for eucalyprobusal C and eucalypcamal K, respectively. The DFT NMR data for the oxygenated carbons C-1 and C-2 shared minimal deviation (<1.5 ppm) in both oxepine FPCs **1b** and **2b**, while in **1a** and **2a**, large errors ranging from 6.5 to 11.3 ppm were observed. These findings substantiate the reassignment of C-1 from an ether to an alcohol, as well as C-2 from an alcohol to an ether, alongside subsequent ring-expansion from pyrano to oxepine FPC structures for both **1b** and **2b**. Comparative ^1^H NMR analyses were also performed with the DFT-calculated NMR data for the revised structures, **1b** (^1^H MAE = 0.11, RMSD = 0.13) and **2b** (^1^H MAE = 0.10, RMSD = 0.12), displaying lower errors than those of the incorrectly assigned **1a** (^1^H MAE = 0.32, RMSD = 0.37) and **2b** (^1^H MAE = 0.24; RMSD = 0.30; Figure 5A,B and Tables S3, S4, S7, and S8). Moreover, the DFT-calculated NMR shielding tensors for the incorrect and revised structures of eucalyprobusal C and eucalypcamal K were analyzed using DP4+ Bayesian theorem probability analyses [28]. Unsurprisingly, and consistent with our comparative analyses of the scaled DFT NMR chemical shifts outlined above, DP4+ unequivocally supported the revised structures **1b** and **2b** with 100% probability over **1a** and **2a** (Tables S11 and S12). 

### 2.4. TDDFT ECD Comparison of Revised FPC Structures (***1b*** and ***2b***) with Experimental ECD Data Reported for Eucalyprobusal C and Eucalypcamal K

**Figure 6:**
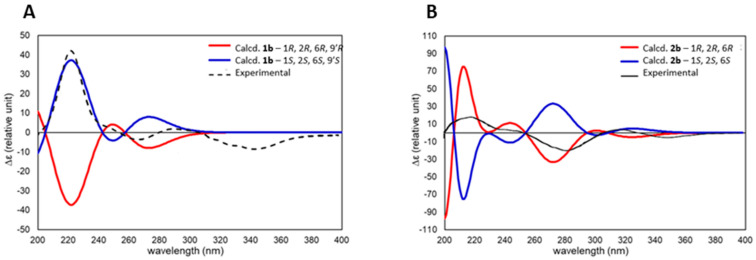
(**A**): TDDFT-calculated ECD spectra for eucalyprobusal C (**1b**—1*S*, 2*S*, 6*S*, and 9′*S*) overlayed with experimental ECD spectra reported for eucalyprobusal C. (**B**): TDDFT-calculated ECD spectra for eucalypcamal K (**2b**—1*R*, 2*R*, and 6*R*) overlayed with experimental ECD spectra reported for eucalypcamal K.

With the revised structures for eucalyprobusal C (**1b**) and eucalypcamal K (**2b**) affirmed by reinterpretation of their experimental NMR data, alongside comparative and probabilistic DFT NMR analyses, TDDFT ECD calculations were performed to assign their absolute configurations. The TDDFT-calculated ECD spectra for **1b** and **2b** were compared with the experimental ECD data published for eucalyprobusal C and eucalypcamal K (Figure 6A,B) [30,31]. Both reassigned structures **1b** and **2b** were found to be excellent matches, with their published experimental ECD spectra confirming the reassignment of absolute configurations. 

Eucalyprobusal C should be revised to structure **1b** with the absolute configuration 1*S*, 2*S*, 6*S*, and 9′*S*, while eucalypcamal K is revised to **2b** with the absolute configuration 1*R*, 2*R*, and 6*R*.

## 3. Discussion

New diformyl and acyl formyl phloroglucinol NPs continue to be reported from Myrtaceae species on a regular basis; however, approximately 10% of all published FPCs have wrongly assigned structures and/or resonances [7]. FPCs containing oxepine ring systems are rare, yet they contain characteristic phloroglucinol ^1^H and ^13^C resonances that differentiate them from the more commonly reported pyrano-containing FPCs. Eucalyprobusal C (**1b**) is only the ninth oxepine-diformyl phloroglucinol meroterpene reported to date, while eucalypcamal K (**2b**) is just the second oxepine 1-formyl-3-acyl phloroglucinol meroterpene reported. Interestingly, eucalyprobusal C is the first oxepine-diformyl phloroglucinol conjugated to a monoterpene, with the eight previously reported NPs in this subclass all containing sesquiterpenes conjugated to the phloroglucinol core. The observation that **1b** and **2b** possess opposite absolute configurations associated with the monoterpene moieties reflects the diversity of terpene building blocks produced by different species of highly speciose genus *Eucalyptus.* Although both compounds have been isolated from species from the Symphyomyrtus sub-genus *E. robusta*, the source of eucalyprobusal C is in the section Latoangulatae, while for *E. camaldulensis*, the source of eucalypcamal K is in the section Exsertaria.

We have previously demonstrated that despite accurate methods to establish correct molecular structures and definitively assign ^1^H and ^13^C NMR resonances available to both authors and peer reviewers, wrongly assigned NP structures and/or incorrectly assigned ^1^H and ^13^C NMR continue to be published in the literature. Our application of computational pattern recognition of NMR data to propose substructure motifs, followed by the verification of these structures using DFT methods, represents an effective and unique approach that has now resulted in the structure revision of five FPCs [7]. These structure corrections complement an additional thirteen plant and marine NP structures that we have corrected based on the reinterpretation of their reported NMR data [8,10,33,34,35]. It is incumbent upon peer reviewers of NP structures to act as gate keepers in an effort to filter out poor interpretation of NMR spectroscopic data; unfortunately however, there are many instances where this process continues to fail [5,36,37]. The development of more tools, such as our NMR fingerprinting PCA methodology, can support researchers and the peer review process to help to reduce the number of erroneous NP structure assignments and prevent their proliferation throughout the literature. This is particularly important for the current and future development of machine learning and AI tools toward automating the structure analysis of complex NPs. Fast methods to analyze big data sets are also becoming increasingly important. DFT NMR calculation methods that offer more computational efficiency, such as DP4, J-DP4, and DP4+ [27,28,29], are excellent choices over more computationally demanding ones at higher levels of theory. 

## 4. Materials and Methods

### 4.1. NMR Fingerprint Visualization, Statistical, and Principal Component Analyses

The visualization and analysis of the literature chemical shift data was performed using the same protocol previously reported [7] within the freely available OSIRIS DataWarrior (version 5.2.1) software [38]. The principal component analysis function within DataWarrior was used to analyze the carbon and proton chemical shift data for 179 formyl phloroglucinol NPs reported in the literature with NMR data recorded in CDCl_3_. PC1 and PC2 were generated with the native visualization function included in the DataWarrior software package (version 5.2.1).

### 4.2. Computational Methods

Extensive conformer searches were performed on **1a**, **1b**, **2a**, and **2b** within the Schrodinger Macromodel (version 10.7) software suite using the Monte Carlo Multiple Minimum (MCMM) method at an energy window of 21.0 kJ/mol and the MMFF forcefield. The step count for Macromodel conformer searches were set so that all low energy conformers were found at least 10 times. The conformer sets for each of the candidate structures (**1a**, **1b**, **2a**, and **2b**) were subjected to gas-phase geometry optimizations (GO) at the B3LYP/6-31+G(d,p) level of theory within Gaussian 16 (Revision C.01) [39]. The GO sets were filtered for duplicate and high-energy conformers (>3.0 kcal/mol above the energy minimum removed). For NMR calculations, ^1^H and ^13^C GIAO NMR DFT chemical shifts were calculated at the mPW1PW91/6-311+G(d,p) level of theory, which included the polarizable continuum PCM solvent model for chloroform [40]. The DFT-calculated NMR isotropic shielding tensors were Boltzmann-averaged across each of the conformational suites (energies < 3.0 kcal/mol) and scaled according to linear regression scaling factors deposited within online resources provided by the Cheshire Chemical Shift Repository (http://cheshirenmr.info/index.htm, accessed 23 October 2023) [41,42]. 

For ECD calculations, the filtered GO conformers used for GIAO NMR calculations (B3LYP/6-31+G(d,p)) were promoted to TDDFT rotational strength and electronic transition calculations using the CAM-B3LYP/6-311+G(d,p) level of theory, with D3 empirical dispersion and the PCM solvent model for chloroform included. The resultant TDDFT-calculated UV and ECD spectra were Boltzmann-weighted and matched with experimental UV and ECD data using the freely available SpecDis (1.71) software [43]. A Gaussian band shape of (eV) of 0.23 and UV corrections of −8 and +7 were applied to **1b** and **2b**, respectively, to match with the published ECD spectra reported for eucalyprobusal C and eucalypcamal K [30,31]. Automation processes with the high-performance computing cluster (‘Gowonda’) were carried out using customized Python scripts [44].

## 5. Conclusions

In conclusion, the incorrectly assigned structures for two FPCs isolated from *Eucalyptus* species, eucalyprobusal C (**1a**) and eucalypcamal K (**2a**), were unequivocally revised to **1b** and **2b**, respectively. Utilizing our previously established NMR fingerprinting method, now expanded to include diagnostic NMR data for 179 FPCs, we identified eucalyprobusal C (**1a**) and eucalypcamal K (**2a**) as having structures inconsistent with their assigned structure classes. Specifically, **1a**, originally identified as a diformyl-pyrano phloroglucinol, and **2a**, designated as a 3-acyl-1-formyl pyrano phloroglucinol, were found to be better matched with NMR fingerprints associated with oxepine-formyl phloroglucinols. After the extensive reanalysis of their reported experimental NMR data and comparison with similar FPC structures in the primary literature, we revised their structures to oxepine-formyl phloroglucinol structures **1b** and **2b**. Subsequent GIAO DFT ^1^H and ^13^C NMR calculations were performed on both the incorrectly assigned structures (**1a** and **2a**) and the revised structures (**1b** and **2b**), followed by extensive comparative analyses using their respective experimental NMR data. The DFT-calculated NMR data for the revised structures **1b** and **2b** were found to be in excellent agreement with the reported experimental NMR data for eucalyprobusal C and eucalypcamal K, respectively. In addition, their absolute configurations were determined by comparing the TDDFT-calculated ECD spectra of the revised structures (**1b** and **2b**) with their published experimental ECD data. By extension, DP4+ Bayesian probability analyses showed 100% probability for the revised structures of eucalyprobusal C (**1b**) and eucalypcamal K (**2b**) over **1a** and **2a**. These structure corrections helped us to refine the data that are publicly available for accurate applications of NMR data for machine learning to aid structure determination of unknown FPCs that might be identified in the future.

The workflow presented herein further outlines the utility of NMR fingerprinting for identifying incorrectly assigned NPs in the literature and associated databases. In combination with computational DFT NMR calculations, we have provided a powerful method for revising the structures of complex NPs. The broad scope of our FPC NMR fingerprinting method also has other demonstrated uses, including the targeting of extracts that contain FPCs and/or identifying subclasses of FPCs within complex NP mixtures [7]. Future applications for NMR fingerprinting should extend to mining subclasses of FPCs from complex NP extracts, particularly efforts targeting specific biological activities such as those currently prioritized for drug resistance (anti-infective ones). Moreover, extending NMR fingerprinting analyses to other valuable subclasses of NPs would provide valuable tools for the many diverse research areas where NPs are of central importance and should decrease the number of incorrect NP structures reported in the literature.

## Data Availability

Supporting data are available in the Electronic Supporting Information (ESI) provided.

## Supplementary Materials

The following supporting information can be downloaded via this link: https://www.mdpi.com/article/10.3390/molecules29030594/s1, Table S1–S8; Comparative experimental (eucalyprobusal C and eucalypcamal K) and DFT calculated NMR data and MAE/RMSD for **1a**, **1b**, **2a**, and **2b**, Table S9; Comparison of eucalyprobusal C (**1a**) experimental NMR data with diagnostic ^1^H and ^13^C NMR data fingerprint data for pyrano-diformyl phloroglucinols and oxepine-formyl phloroglucinols, Table S10; Comparison of eucalypcamal K (2a) experimental NMR data with diagnostic ^1^H and ^13^C NMR data fingerprint data for pyrano-3-acyl-1-formyl phloroglucinols and oxepine-formyl phloroglucinols, Table S11; Eucalyprobusal C incorrect (**1a** = Isomer 1 − 0.00%) versus revised (**1b** = Isomer 2 – 100.00%) DP4+ output, Table S12; Eucalypcamal K incorrect (**2a** = Isomer 1 − 0.00%) versus revised (**2b** = Isomer 2 − 100.00%) DP4+ output, Figure S1; Classification of subclasses for formyl phloroglucinol meroterpenes (image with adapted from Baxter et al.), Tables S13–S16; Eucalyprobusal C (**1a** and **1b**) and eucalypcamal K (**2a** and **2b**) conformational sets, energies, and distributions for GIAO-DFT NMR and TDDFT-ECD calculations, and Tables S17–20; Eucalyprobusal C (**1a** and **1b**) and eucalypcamal K (**2a** and **2b**) DFT geometry optimized conformers calculated at the B3LYP/6-31+G(d,p) level of theory.
