# Supplementary material for: Structure Revision of Formyl Phloroglucinol Meroterpenoids: A Unified Approach Using NMR Fingerprinting and DFT NMR and ECD Analyses
Source: Molecules. 2024 Jan 25;29(3):594. doi: 10.3390/molecules29030594 (PMC10856187; doi:10.3390/molecules29030594)
Supplement: Supplementary file 1 [file molecules-29-00594-s001.zip › molecules-2823724-supplementary.pdf]

Structure Revision of Formyl Phloroglucinol Meroterpenoids: A Unified Approach Using NMR Fingerprinting and DFT NMR and ECD Analyses

Darren C. Holland [1,2,*] and Anthony R. Carroll [1,2,*]

[1] School of Environment and Science, Griffith University, Queensland 4111, Australia

[2] Griffith Institute for Drug Discovery, Griffith University, Nathan, Queensland 4111, Australia

[*]Correspondence: darren.holland@griffith.edu.au (D.C.H.); a.carroll@griffith.edu.au (A.R.C.)

1. Table of Contents

**Table S1**	Eucalyprobusal C (**1a**) comparative ^{13}C NMR data with DFT-calculated ^{13}C NMR data for incorrect structure **1a**.
**Table S2**	Eucalyprobusal C (**1a**) comparative ^{13}C NMR data with DFT-calculated ^{13}C NMR data for revised structure **1b**.
**Table S3**	Eucalyprobusal C (**1a**) comparative ^{1}H NMR data with DFT-calculated ^{1}H NMR data for incorrect structure **1a**.
**Table S4**	Eucalyprobusal C (**1a**) comparative ^{1}H NMR data with DFT-calculated ^{1}H NMR data for revised structure **1b**.
**Table S5**	Eucalypcamal K (**2a**) comparative ^{13}C NMR data with DFT-calculated ^{13}C NMR data for incorrect structure **2a**.
**Table S6**	Eucalypcamal K (**2a**) comparative ^{13}C NMR data with DFT-calculated ^{13}C NMR data for revised structure **2b**.
**Table S7**	Eucalypcamal K (**2a**) comparative ^{1}H NMR data with DFT-calculated ^{1}H NMR data for incorrect structure **2a**.
**Table S8**	Eucalypcamal K (**2a**) comparative ^{1}H NMR data with DFT-calculated ^{1}H NMR data for incorrect structure **2b.**
**Table S9**	Comparison of eucalyprobusal C (**1a**) experimental NMR data with diagnostic ^{1}H and ^{13}C NMR data fingerprint data for pyrano-diformyl phloroglucinols and oxepine-formyl phloroglucinols.
**Table S10**	Comparison of eucalypcamal K (**2a**) experimental NMR data with diagnostic ^{1}H and ^{13}C NMR data fingerprint data for pyrano-3-acyl-1-formyl phloroglucinols and oxepine-formyl phloroglucinols.
**Table S11**	Eucalyprobusal C incorrect (**1a** = Isomer 1- 0.00%) versus revised (**1b** = Isomer 2 – 100.00%) DP4+ output.
**Table S12**	Eucalypcamal K incorrect (**2a** = Isomer 1 - 0.00%) versus revised (**2b** = Isomer 2 - 100.00%) DP4+ output.
**Figure S1**	Classification of subclasses for formyl phloroglucinol meroterpenes (image with adapted from Baxter et al.)
**Table S13**	Eucalyprobusal C (**1a**) conformational set, energies, and distributions for GIAO-DFT NMR and TDDFT-ECD calculations.
**Table S14**	Eucalyprobusal C (**1b**) conformational set, energies, and distributions for GIAO-DFT NMR and TDDFT-ECD calculations.
**Table S15**	Eucalypcamal K (**2a**) conformational set, energies, and distributions for GIAO-DFT NMR and TDDFT-ECD calculations.
**Table S16**	Eucalypcamal K (**2b**) conformational set, energies, and distributions for GIAO-DFT NMR and TDDFT-ECD calculations.
**Table S17**	Eucalyprobusal C (**1a**) DFT geometry optimized conformers calculated at the B3LYP/6-31+G(d,p) level of theory.
**Table S18**	Eucalyprobusal C (**1b**) DFT geometry optimized conformers calculated at the B3LYP/6-31+G(d,p) level of theory.
**Table S19**	Eucalypcamal K (**2a**) DFT geometry optimized conformers calculated at the B3LYP/6-31+G(d,p) level of theory.
**Table S20**	Eucalypcamal K (**2b**) DFT geometry optimized conformers calculated at the B3LYP/6-31+G(d,p) level of theory.

2. References

Table S1. Eucalyprobusal C (**1a**)[1] comparative ^{13}C NMR data with DFT-calculated ^{13}C NMR data for incorrect structure **1a**.

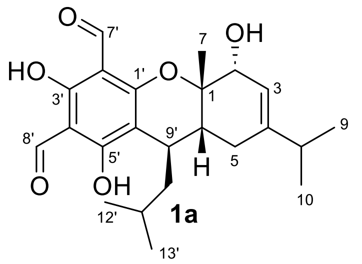

1a

Pos.	Exp. δ_C 1a[1]	DFT calc. δ_C 1a	Abs. error
1	72.7	80.9	8.2
2	80.2	73.7	6.5
3	111.6	120.9	9.3
4	155.2	150.0	5.2
5	32.4	30.6	1.8
6	40.2	36.3	3.9
7	26.4	21.3	5.1
8	34.5	38.5	4.0
9	21.1	19.9	1.2
10	20.9	19.8	1.1
1'	165.0	163.0	2.0
2'	109.1	104.6	4.5
3'	167.3	169.1	1.8
4'	105.6	104.1	1.5
5'	169.1	171.0	1.9
6'	117.4	104.5	12.9
7'	193.7	190.9	2.8
8'	192.3	191.5	0.8
9'	35.9	35.8	0.1
10'	46.7	39.3	7.4
11'	25.6	27.7	2.1
12'	22.3	18.9	3.4
13'	22.8	22.5	0.3
		**^{13}C MAE =**	**3.8**
		**^{13}C RMSD =**	**4.96**

Table S2. Eucalyprobusal C (**1a**)[1] comparative ^{13}C NMR data with DFT-calculated ^{13}C NMR data for revised structure **1b**.

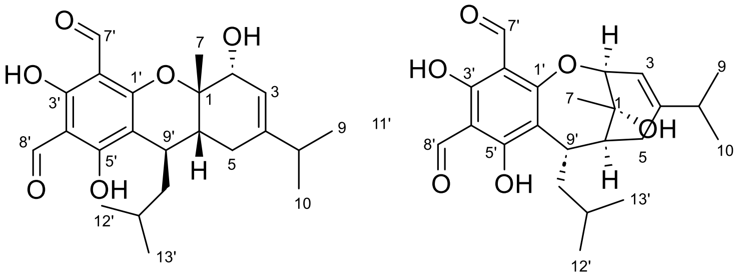

1a **1b**

Pos.	Exp. δ_C 1a[1]	DFT calc. δ_C 1b	Abs. error
1	72.7	74.2	1.5
2	80.2	81.7	1.5
3	111.6	113.1	1.5
4	155.2	160.2	5.0
5	32.4	33.4	1.0
6	40.2	41.6	1.4
7	26.4	24.8	1.6
8	34.5	38.7	4.2
9	21.1	20.2	0.9
10	20.9	19.4	1.5
1'	165.0	166.4	1.4
2'	109.1	109.0	0.1
3'	167.3	168.7	1.4
4'	105.6	105.7	0.1
5'	169.1	170.3	1.2
6'	117.4	117.1	0.3
7'	193.7	193.5	0.2
8'	192.3	192.0	0.3
9'	35.9	37.7	1.8
10'	46.7	45.4	1.3
11'	25.6	28.4	2.8
12'	22.3	20.2	2.1
13'	22.8	20.9	1.9
		**^{13}C MAE =**	**1.5**
		**^{13}C RMSD =**	**1.92**

Table S3. Eucalyprobusal C (**1a**)[1] comparative ^{1}H NMR data with DFT-calculated ^{1}H NMR data for incorrect structure **1a**.

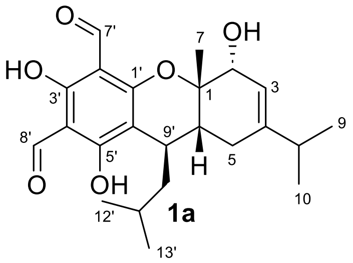

1a

Pos.	Exp. δ_H 1a[1]	DFT calc. δ_H 1a	Abs. error
2	4.49	4.13	0.03
3	5.10	5.51	0.16
5	2.62	2.08	0.17
	1.86	1.74	0.09
6	2.23	2.33	0.04
7	1.65	1.75	0.05
8	2.06	2.31	0.13
9	0.74	1.07	0.17
10	0.72	0.99	0.15
7’	10.10	9.80	0.30
8’	10.21	10.00	0.21
9'	3.52	2.76	0.01
10'	1.42	1.86	0.04
	1.27	1.80	0.07
11'	1.47	1.89	0.23
12'	0.93	1.08	0.03
13'	0.85	1.01	0.09
		**^{1}H MAE =**	**0.32**
		**^{1}H RMSD =**	**0.37**

Table S4. Eucalyprobusal C (**1a**)[1] comparative ^{1}H NMR data with DFT-calculated ^{13}C NMR data for revised structure **1b**.

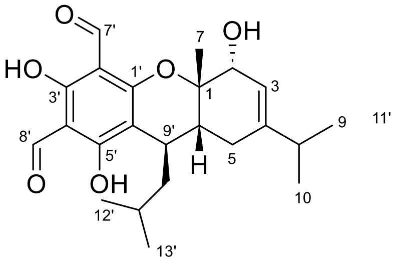

1a

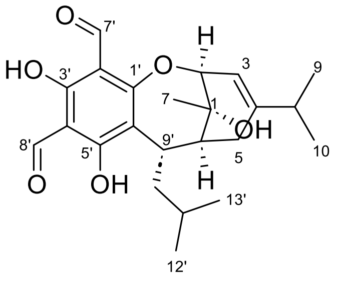

1b

Pos.	Exp. δ_H 1a[1]	DFT calc. δ_H 1b	Abs. error
2	4.49	4.52	0.03
3	5.10	5.26	0.16
5	2.62	2.79	0.17
	1.86	1.95	0.09
6	2.23	2.27	0.04
7	1.65	1.60	0.05
8	2.06	2.19	0.13
9	0.74	0.91	0.17
10	0.72	0.87	0.15
7’	10.10	9.90	0.20
8’	10.21	10.00	0.11
9'	3.52	3.53	0.01
10'	1.42	1.46	0.04
	1.27	1.20	0.07
11'	1.47	1.70	0.23
12'	0.93	0.97	0.03
13'	0.85	0.94	0.09
		**^{1}H MAE =**	**0.11**
		**^{1}H RMSD =**	**0.13**

Table S5. Eucalypcamal K (**2a**)[2] comparative ^{13}C NMR data with DFT-calculated ^{13}C NMR data for incorrect structure **2a**.

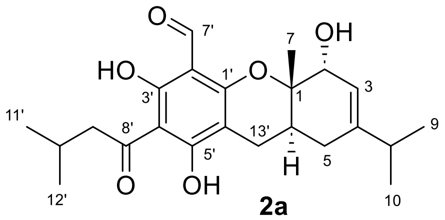

2a

Pos.	Exp. δ_C 2a[1]	DFT calc. δ_C 2a	Abs. error
1	72.4	83.7	11.3
2	79.9	70.6	9.3
3	112.2	120.3	8.1
4	154.7	153.9	0.8
5	28.8	32.5	3.7
6	36.7	29.4	7.3
7	24.0	15.0	9.0
8	34.5	38.8	4.3
9	21.0	20.2	0.8
10	20.1	19.7	0.4
1'	164.6	161.2	3.4
2'	108.4	104.2	4.2
3'	167.3	169.3	2.0
4'	105.6	103.6	2.0
5'	171.5	171.1	0.4
6'	112.3	101.4	10.9
7'	193.4	190.0	3.4
8'	207.0	206.1	0.9
9'	52.9	53.3	0.4
10'	25.3	26.9	1.6
11'	22.6	26.9	4.3
12'	22.7	21.1	1.6
13'	24.1	22.3	1.8
		**^{13}C MAE =**	**4.0**
		**^{13}C RMSD =**	**5.29**

Table S6. Eucalypcamal K (**2a**)[2] comparative ^{13}C NMR data with DFT-calculated ^{13}C NMR data for revised **2b**.

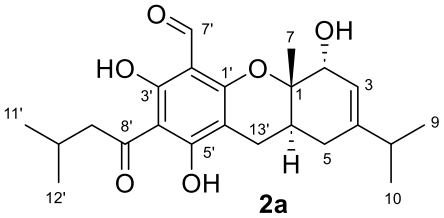

2a

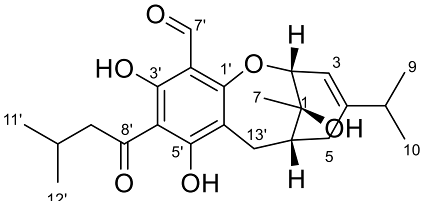

2b

Pos.	Exp. δ_C 2a[2]	DFT calc. δ_C 2b	Abs. error
1	72.4	73.7	1.3
2	79.9	81.1	1.2
3	112.2	113.8	1.6
4	154.7	159.9	5.2
5	28.8	29.8	1.0
6	36.7	39.3	2.6
7	24.0	22.5	1.5
8	34.5	38.6	4.1
9	21.0	20.1	0.9
10	20.1	19.7	0.4
1'	164.6	165.3	0.7
2'	108.4	108.2	0.2
3'	167.3	169.0	1.7
4'	105.6	105.4	0.2
5'	171.5	172.0	0.5
6'	112.3	112.0	0.3
7'	193.4	192.7	0.7
8'	207.0	206.6	0.4
9'	52.9	53.3	0.4
10'	25.3	27.1	1.8
11'	22.6	20.8	1.8
12'	22.7	21.0	1.7
13'	24.1	25.3	1.2
		**^{13}C MAE =**	**1.4**
		**^{13}C RMSD =**	**1.82**

Table S7. Eucalypcamal K (**2a**)[2] comparative ^{1}H NMR data with DFT-calculated ^{1}H NMR data for incorrect structure **2a**.

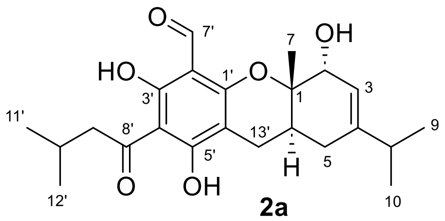

2a

Pos.	Exp. δ_H 2a[2]	DFT calc. δ_H 2a	Abs. error
2	4.51	4.20	0.31
3	5.16	5.74	0.58
5	1.96	1.93	0.03
	2.42	2.49	0.07
6	2.21	2.47	0.26
7	1.58	1.20	0.38
8	2.05	2.42	0.37
9	0.71	1.12	0.41
10	0.70	1.10	0.40
7'	10.05	9.84	0.21
9'	2.92	3.03	0.11
	3.05	3.06	0.01
10'	2.21	2.37	0.16
11'	0.96	1.06	0.10
12'	0.94	1.05	0.11
13'	2.28	2.19	0.09
	3.34	2.81	0.53
		**^{1}H MAE =**	**0.24**
		**^{1}H RMSD =**	**0.30**

Table S8. Eucalypcamal K (**2a**)[2] comparative ^{1}H NMR data with DFT-calculated ^{1}H NMR data for revised **2b**.

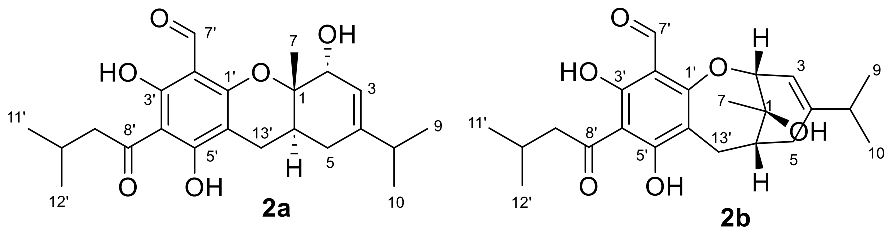

Pos.	Exp. δ_H 2a[2]	DFT calc. δ_H 2b	Abs. error
2	4.51	4.56	0.05
3	5.16	5.36	0.20
5	1.96	2.04	0.08
	2.42	2.58	0.16
6	2.21	2.20	0.01
7	1.58	1.54	0.04
8	2.05	2.19	0.14
9	0.71	0.91	0.20
10	0.70	0.85	0.15
7'	10.05	9.88	0.17
9'	2.92	3.02	0.10
	3.05	3.06	0.01
10'	2.21	2.35	0.14
11'	0.96	1.04	0.08
12'	0.94	1.03	0.09
13'	2.28	2.31	0.03
	3.34	3.38	0.04
		**^{1}H MAE =**	**0.10**
		**^{1}H RMSD =**	**0.12**

Table S9. Comparison of eucalyprobusal C (**1a**) experimental data with pyrano-diformyl phloroglucinols and oxepine-formyl phloroglucinols (NMR data adapted from Tables S04 and S09 from our publication Baxter et al. – https://doi.org/10.1021/acs.jnatprod.3c00139).[3]

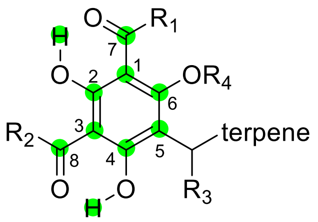

R_1 = H or carbon chain
R_2 = H or carbon chain
R_3 = alkyl group
R_4 = H or terpene

pyrano-diformyl phloroglucinols	C-1	C-2	C-3	C-4	C-5	C-6	C-7	H-7	C-8	H-8
Eucalyprobusal C (**1a**)	109.1	167.3	105.6	169.1	117.4	165.0	193.7	10.10	192.3	10.21
Lower Limit	103.6	166.9	103.4	167.5	104.9	160.8	190.2	9.96	191.3	10.00
Upper Limit	106.9	168.5	106.0	171.4	114.1	166.6	192.7	10.27	192.9	10.27
Upper and lower limit abs. error	2.2-5.5				3.3-12.5		3.5-1.0			

oxepine-formyl phloroglucinols	C-1	C-2	C-3	C-4	C-5	C-6	C-7	H-7	C-8	H-8
Eucalyprobusal C (**1a**)	109.1	167.3	105.6	169.1	117.4	165.0	193.7	10.10	192.3	10.21
Lower Limit	107.6	167.2	104.6	169.5	115.9	165.0	193.3	10.09	192.1	10.20
Upper Limit	108.8	167.8	106.1	170.5	117.8	168.4	193.7	10.24	192.8	10.27
Upper and lower limit abs. error	0.3-2.2			0.4-1.4						

Table S10. Comparison of eucalypcamal K (**2a**) experimental data with pyrano-3-acyl-1-formyl phloroglucinols and oxepine-formyl phloroglucinols (NMR data adapted from Tables S02 and S09 from our publication Baxter et al. - https://doi.org/10.1021/acs.jnatprod.3c00139).[3]

pyrano-3-acyl-1-formy phloroglucinols	C-1	C-2	C-3	C-4	C-5	C-6	C-7	H-7
Eucalypcamal K (**2a**)	108.4	167.4	105.6	171.5	112.3	164.6	193.4	10.05
Lower Limit	103.7	168.0	103.1	170.9	98.5	160.4	191.4	9.91
Upper Limit	104.4	168.4	103.9	172.1	102.0	164.3	192.0	10.05
Upper and lower limit abs. error	4.0-4.7		1.7-2.5		10.3-13.8	0.3-4.3	1.4-2.0	

oxepine-formyl phloroglucinols	C-1	C-2	C-3	C-4	C-5	C-6	C-7	H-7
Eucalypcamal K (**2a**)	108.4	167.4	105.6	171.5	112.3	164.6	193.4	10.05
Lower Limit	107.6	167.2	104.6	169.5	115.9	166.9	193.3	10.09
Upper Limit	108.8	167.8	106.1	170.5	117.8	168.4	193.6	10.24
Upper and lower limit abs. error				1.0-2.0	3.6-5.5	2.3-3.8		0.04-0.19

Table S11. Eucalyprobusal C incorrect (**1a** = Isomer 1- 0.00%) versus revised (**1b** = Isomer 2 – 100.00%) DP4+ output.[4]

Functional	Solvent?		Basis Set		Type of Data	
mPW1PW91	PCM		6-311+G(d,p)		Shielding Tensors	
	Isomer 1	Isomer 2	Isomer 3	Isomer 4	Isomer 5	Isomer 6
sDP4+ (H data)	0.00%	100.00%	-	-	-	-
sDP4+ (C data)	0.00%	100.00%	-	-	-	-
sDP4+ (all data)	0.00%	100.00%	-	-	-	-
uDP4+ (H data)	0.01%	99.99%	-	-	-	-
uDP4+ (C data)	0.00%	100.00%	-	-	-	-
uDP4+ (all data)	0.00%	100.00%	-	-	-	-
DP4+ (H data)	0.00%	100.00%	-	-	-	-
DP4+ (C data)	0.00%	100.00%	-	-	-	-
DP4+ (all data)	0.00%	100.00%	-	-	-	-

Table S12. Eucalypcamal K incorrect (**2a** = Isomer 1 - 0.00%) versus revised (**2b** = Isomer 2 - 100.00%) DP4+ output.[4]

Functional	Solvent?		Basis Set		Type of Data	
mPW1PW91	PCM		6-311+G(d,p)		Shielding Tensors	
	Isomer 1	Isomer 2	Isomer 3	Isomer 4	Isomer 5	Isomer 6
sDP4+ (H data)	0.00%	100.00%	-	-	-	-
sDP4+ (C data)	0.00%	100.00%	-	-	-	-
sDP4+ (all data)	0.00%	100.00%	-	-	-	-
uDP4+ (H data)	0.15%	99.85%	-	-	-	-
uDP4+ (C data)	0.00%	100.00%	-	-	-	-
uDP4+ (all data)	0.00%	100.00%	-	-	-	-
DP4+ (H data)	0.00%	100.00%	-	-	-	-
DP4+ (C data)	0.00%	100.00%	-	-	-	-
DP4+ (all data)	0.00%	100.00%	-	-	-	-

Figure S1. Classification of subclasses for formyl phloroglucinol meroterpenes (image adapted from our publication Baxter et al. - https://doi.org/10.1021/acs.jnatprod.3c00139).[3]

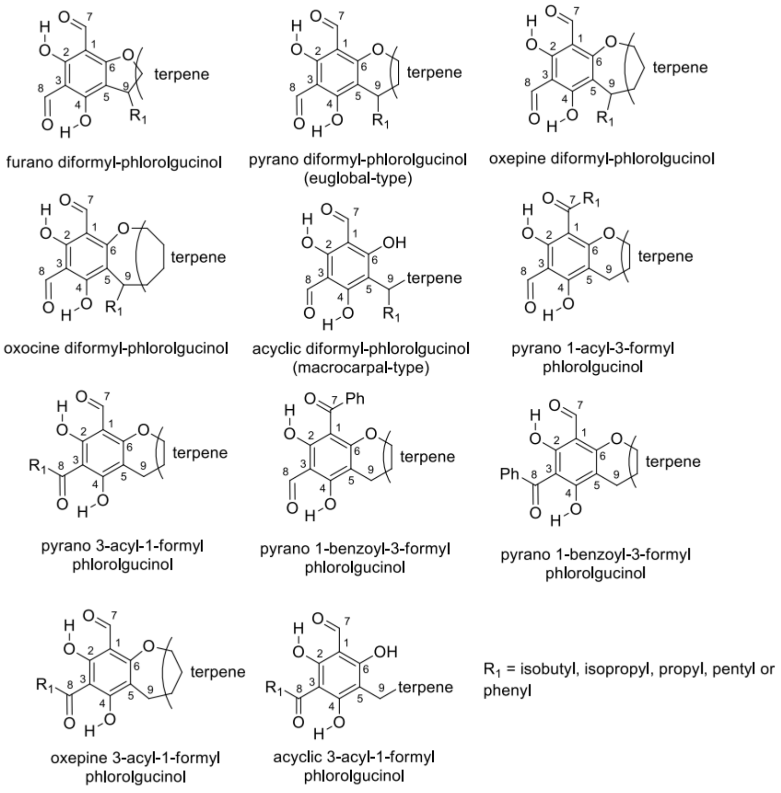

Table S13. Eucalyprobusal C (**1a**) conformational set, energies, and distributions for GIAO-DFT NMR and TDDFT-ECD calculations.

Conformer Number (1a)	Energy (a.u.)	Energy (kcal/mol)	Relative Energy (kcal/mol)	Boltzmann Factor	Equilibrium Mole Fraction
9	-1345.526978	-844330.8145	0	1.000	0.350
24	-1345.526167	-844330.3056	0.508910116	0.423	0.148
12	-1345.526117	-844330.2743	0.540285586	0.401	0.140
4	-1345.525285	-844329.7522	1.062373399	0.166	0.058
11	-1345.525213	-844329.707	1.107554075	0.154	0.054
2	-1345.524797	-844329.4459	1.368597982	0.099	0.035
3	-1345.524691	-844329.3794	1.435113977	0.088	0.031
6	-1345.524157	-844329.0443	1.770203992	0.050	0.018
5	-1345.523841	-844328.846	1.96849696	0.036	0.013

**conformational sets shown filtered for duplicate conformers, imaginary frequencies, and energies >3.0 kcal.mol*

Table S14. Eucalyprobusal C (**1b**) conformational set, energies, and distributions for GIAO-DFT NMR and TDDFT-ECD calculations.

Conformer Number (1b)	Energy (a.u.)	Energy (kcal/mol)	Relative Energy (kcal/mol)	Boltzmann Factor	Equilibrium Mole Fraction
4	-1345.509007	-844319.5376	0	1.000	0.137
1	-1345.508787	-844319.3995	0.138052066	0.792	0.108
19	-1345.508582	-844319.2709	0.266691491	0.637	0.087
3	-1345.508365	-844319.1347	0.402861029	0.506	0.069
8	-1345.508348	-844319.124	0.413528689	0.497	0.068
20	-1345.508316	-844319.104	0.433608989	0.481	0.066
9	-1345.508304	-844319.0964	0.441139102	0.475	0.065
6	-1345.508262	-844319.0701	0.467494496	0.454	0.062
10	-1345.508115	-844318.9778	0.559738377	0.388	0.053
27	-1345.507869	-844318.8235	0.714105687	0.299	0.041
16	-1345.507812	-844318.7877	0.749873722	0.282	0.038
17	-1345.507677	-844318.703	0.83458749	0.244	0.033
28	-1345.507671	-844318.6992	0.838352546	0.243	0.033
24	-1345.507643	-844318.6816	0.855922809	0.235	0.032
13	-1345.507594	-844318.6509	0.88667077	0.224	0.031
14	-1345.507285	-844318.457	1.080571171	0.161	0.022
5	-1345.505506	-844317.3407	2.196910378	0.024	0.003
21	-1345.505089	-844317.079	2.458581794	0.016	0.002
11	-1345.505033	-844317.0438	2.49372232	0.015	0.002
23	-1345.504925	-844316.9761	2.561493334	0.013	0.002
7	-1345.504814	-844316.9064	2.631146877	0.012	0.002
29	-1345.50446	-844316.6843	2.853285201	0.008	0.001
31	-1345.504301	-844316.5845	2.953059194	0.007	0.001

**conformational sets shown filtered for duplicate conformers, imaginary frequencies, and energies >3.0 kcal.mol*

Table S15. Eucalypcamal K (**2a**) conformational set, energies, and distributions for GIAO-DFT NMR and TDDFT-ECD calculations.

Conformer Number 2a	Energy (a.u.)	Energy (kcal/mol)	Relative Energy (kcal/mol)	Boltzmann Factor	uilibrium Mole Fracti
12	-1345.542744	-844340.7079	0	1.000	0.070
26	-1345.542741	-844340.706	0.001882528	0.997	0.070
23	-1345.542656	-844340.6526	0.055220826	0.911	0.064
7	-1345.54199	-844340.2347	0.473142081	0.450	0.031
33	-1345.541887	-844340.1701	0.537775548	0.403	0.028
8	-1345.541856	-844340.1506	0.557228339	0.390	0.027
29	-1345.541801	-844340.1161	0.591741356	0.368	0.026
20	-1345.541709	-844340.0584	0.64947222	0.334	0.023
22	-1345.541699	-844340.0521	0.655747314	0.330	0.023
24	-1345.541079	-844339.663	1.044803136	0.171	0.012
54	-1345.541075	-844339.6605	1.047313174	0.170	0.012
46	-1345.540905	-844339.5539	1.15398977	0.142	0.010
43	-1345.540901	-844339.5514	1.156499808	0.142	0.010
64	-1345.540159	-844339.0857	1.622111776	0.065	0.005
71	-1345.540108	-844339.0537	1.654114755	0.061	0.004
79	-1345.539553	-844338.7055	2.002382467	0.034	0.002
80	-1345.539423	-844338.6239	2.083958688	0.030	0.002
10	-1345.539405	-844338.6126	2.095253857	0.029	0.002
72	-1345.539309	-844338.5524	2.155494758	0.026	0.002
38	-1345.539277	-844338.5323	2.175575059	0.025	0.002
60	-1345.539133	-844338.4419	2.265936411	0.022	0.002
86	-1345.53912	-844338.4338	2.274094033	0.021	0.001
28	-1345.538794	-844338.2292	2.478662094	0.015	0.001
62	-1345.538607	-844338.1118	2.596006351	0.012	0.001
87	-1345.538599	-844338.1068	2.601026426	0.012	0.001
73	-1345.538376	-844337.9669	2.74096102	0.010	0.001
18	-1345.53837	-844337.9631	2.744726076	0.010	0.001
83	-1345.538365	-844337.96	2.747863623	0.010	0.001

**conformational sets shown filtered for duplicate conformers, imaginary frequencies, and energies >3.0 kcal.mol*

Table S16. Eucalypcamal K (**2b**) conformational set, energies, and distributions for GIAO-DFT NMR and TDDFT-ECD calculations.

Conformer Number (2b)	Energy (a.u.)	Energy (kcal/mol)	Relative Energy (kcal/mol)	Boltzmann Factor	Equilibrium Mole Fraction
16	-1345.527498	-844331.1408	0	1	0.058418228
12	-1345.527361	-844331.0549	0.085968787	0.864797634	0.050519945
43	-1345.52685	-844330.7342	0.406626085	0.503048844	0.029387222
35	-1345.526751	-844330.6721	0.468749515	0.452921376	0.026458864
53	-1345.526744	-844330.6677	0.473142081	0.449572223	0.026263213
11	-1345.526708	-844330.6451	0.495732419	0.432735234	0.025279626
13	-1345.526678	-844330.6263	0.514557701	0.419187103	0.024488168
23	-1345.526671	-844330.6219	0.518950266	0.416087401	0.024307089
15	-1345.526547	-844330.5441	0.596761431	0.364825574	0.021312464
17	-1345.526539	-844330.5391	0.601781506	0.361744094	0.021132449
52	-1345.52646	-844330.4895	0.651354748	0.332677688	0.019434441
31	-1345.526396	-844330.4493	0.691515349	0.310851575	0.018159398
61	-1345.526115	-844330.273	0.867845488	0.230759033	0.013480534
58	-1345.525902	-844330.1393	1.001504988	0.184109782	0.010755367
5	-1345.525834	-844330.0967	1.044175627	0.171302756	0.010007203
30	-1345.525828	-844330.0929	1.047940683	0.170216431	0.009943742
45	-1345.525828	-844330.0929	1.047940683	0.170216431	0.009943742
69	-1345.52582	-844330.0879	1.052960758	0.168778707	0.009859753
56	-1345.525799	-844330.0747	1.066138455	0.165062194	0.009642641
21	-1345.52577	-844330.0565	1.084336228	0.16006403	0.009350657
38	-1345.525727	-844330.0295	1.111319131	0.152930175	0.00893391
47	-1345.525705	-844330.0157	1.125124338	0.149404152	0.008727926
42	-1345.525678	-844329.9988	1.142067092	0.145187677	0.008481607
57	-1345.525577	-844329.9354	1.20544554	0.130443206	0.007620261
40	-1345.525057	-844329.6091	1.531750423	0.075157586	0.004390573
63	-1345.525057	-844329.6091	1.531750423	0.075157586	0.004390573
59	-1345.524969	-844329.5539	1.58697125	0.068462183	0.003999439
36	-1345.524962	-844329.5495	1.591363816	0.067955936	0.003969865
50	-1345.524903	-844329.5125	1.62838687	0.063835048	0.00372913
51	-1345.524705	-844329.3882	1.752633729	0.051746921	0.003022963
6	-1345.523972	-844328.9282	2.212598113	0.023787786	0.00138964
60	-1345.523546	-844328.6609	2.479917113	0.015142247	0.000884583
32	-1345.523419	-844328.5812	2.559610806	0.013234563	0.00077314
9	-1345.52336	-844328.5442	2.59663386	0.012432011	0.000726256
46	-1345.523229	-844328.462	2.67883759	0.010819788	0.000632073

**conformational sets filtered for duplicate conformers, imaginary frequencies, and energies >3.0 kcal.mol*

Table S17. Eucalyprobusal C (**1a**) DFT-geometry optimized conformers calculated at the B3LYP/6-31+G(d,p) level of theory.

Conformer 2:

C	-2.950458	-1.827436	0.038254
C	-3.308839	-0.618545	0.677654
C	-2.368646	0.455484	0.674008
C	-1.109088	0.372440	0.040681
C	-0.778959	-0.852768	-0.547665
C	-1.682247	-1.963833	-0.580602
C	-0.124231	1.532432	0.124310
C	1.296890	1.004016	-0.203819
O	0.431799	-1.093272	-1.097379
H	1.949286	1.844096	-0.466655
C	-1.349674	-3.203203	-1.239714
C	-4.581149	-0.467642	1.343879
O	-4.920142	0.570964	1.941563
O	-3.821097	-2.835107	0.027738
O	-2.672790	1.584785	1.315229
O	-2.115784	-4.185433	-1.276861
C	-0.594324	2.788161	-0.661446
C	0.280145	4.053603	-0.519151
C	-0.292983	5.175180	-1.401850
C	0.409331	4.531243	0.936640
C	1.876640	0.323041	1.057883
C	3.123731	-0.503904	0.845114
C	3.470918	-0.890512	-0.389791
C	2.708754	-0.554394	-1.643165
C	3.916926	-0.855466	2.097541
C	3.094757	-1.758329	3.045299
C	5.290671	-1.483868	1.827901
C	1.297130	0.047466	-1.412087
C	0.801650	0.639782	-2.737543
O	2.655399	-1.676266	-2.528191
H	-0.106898	1.834453	1.178903
H	-0.372480	-3.268677	-1.736935
H	-5.269253	-1.323801	1.311927
H	-3.382462	-3.593567	-0.465598
H	-3.591403	1.466148	1.706530
H	-0.696863	2.555161	-1.724500
H	-1.600318	3.033479	-0.304384
H	1.291080	3.833722	-0.892881
H	0.333390	6.072973	-1.356114
H	-0.360034	4.865784	-2.451133
H	-1.301228	5.454983	-1.072019
H	0.988680	5.459728	0.989765
H	0.911728	3.797245	1.575284
H	-0.578633	4.727953	1.370846
H	2.084227	1.106377	1.799495
H	1.102247	-0.307448	1.515593
H	4.370518	-1.473313	-0.566383
H	3.272415	0.197516	-2.211796
H	4.088825	0.094175	2.629202
H	3.658137	-1.959728	3.962722
H	2.145025	-1.299703	3.336160
H	2.874565	-2.719042	2.565546
H	5.827530	-1.625602	2.771574
H	5.907772	-0.850835	1.182193
H	5.197953	-2.467327	1.352993
H	1.301842	1.591223	-2.943649
H	1.032817	-0.058086	-3.545918
H	-0.275495	0.812059	-2.729043
H	2.176276	-2.383012	-2.072493

Conformer 3:

C	-3.042951	-1.631559	0.277203
C	-3.283241	-0.319862	0.747641
C	-2.252421	0.654416	0.591136
C	-1.016012	0.371547	-0.026146

C	-0.811988	-0.939281	-0.464581
C	-1.807132	-1.960811	-0.335723
C	0.068171	1.439049	-0.087028
C	1.425180	0.757543	-0.402574
O	0.352611	-1.341651	-1.018406
H	2.123761	1.512825	-0.781983
C	-1.599293	-3.300558	-0.828178
C	-4.526724	0.036324	1.389701
O	-4.763727	1.175765	1.832638
O	-3.996232	-2.551266	0.415108
O	-2.443866	1.888750	1.059349
O	-2.447004	-4.207795	-0.722627
C	-0.284976	2.661662	-0.993491
C	-0.101639	4.033646	-0.312046
C	1.365274	4.325522	0.043996
C	-0.671364	5.150071	-1.200424
C	2.014297	0.160932	0.896078
C	3.165745	-0.797655	0.706033
C	3.408201	-1.336920	-0.496299
C	2.621371	-1.050600	-1.746360
C	4.026752	-1.143078	1.912754
C	4.882270	0.054277	2.376726
C	3.195202	-1.706870	3.082055
C	1.278817	-0.311960	-1.503362
C	0.738238	0.184832	-2.849488
O	2.429004	-2.238026	-2.518894
H	0.148621	1.836021	0.933697
H	-0.643704	-3.517112	-1.324753
H	-5.287540	-0.751087	1.483398
H	-3.637317	-3.403948	0.021504
H	-3.363186	1.912086	1.464910
H	0.326450	2.645733	-1.902798
H	-1.325999	2.579868	-1.322951
H	-0.683296	4.014418	0.619730
H	1.459524	5.296388	0.542966
H	1.787063	3.572375	0.718739
H	1.989768	4.355741	-0.858593
H	-0.577513	6.127844	-0.714793
H	-1.732451	4.984136	-1.417132
H	-0.137658	5.203542	-2.158261
H	2.323800	0.995678	1.537198
H	1.219217	-0.350851	1.456001
H	4.238496	-2.028179	-0.633388
H	3.214854	-0.404169	-2.406539
H	4.718868	-1.931285	1.588954
H	5.549107	-0.249321	3.191304
H	5.499845	0.440908	1.559448
H	4.262385	0.876201	2.752599
H	3.854575	-2.034460	3.893105
H	2.594978	-2.566881	2.766524
H	2.516562	-0.952899	3.496792
H	1.359259	0.999905	-3.233311
H	0.769887	-0.638642	-3.566823
H	-0.289284	0.544660	-2.772983
H	1.923962	-2.859722	-1.975657

Conformer 4:

C	3.125586	-1.640159	-0.218953
C	3.376576	-0.371799	-0.790713
C	2.358230	0.624676	-0.704335
C	1.124028	0.408171	-0.053001
C	0.901114	-0.871132	0.466481
C	1.886227	-1.909715	0.414581
C	0.052903	1.492033	-0.048048
C	-1.317028	0.837970	0.272056
O	-0.274117	-1.235502	1.024532
H	-2.023133	1.609067	0.599744
C	1.663435	-3.208041	1.002465
C	4.617426	-0.084831	-1.471345
O	4.862487	1.009925	-2.012062
O	4.069640	-2.577035	-0.286304

O	2.560893	1.809678	-1.281974
O	2.502112	-4.129119	0.965444
C	0.444483	2.731383	0.803970
C	-0.523591	3.933961	0.750333
C	-0.018225	5.041750	1.689981
C	-0.715282	4.484823	-0.672368
C	-1.875662	0.197094	-1.019223
C	-3.028929	-0.760750	-0.839986
C	-3.313017	-1.259943	0.369681
C	-2.574079	-0.914223	1.634321
C	-3.803437	-1.107019	-2.105392
C	-4.540177	-2.453052	-2.051023
C	-4.785338	0.027049	-2.477848
C	-1.219486	-0.186723	1.419230
C	-0.746534	0.365123	2.770018
O	-2.415964	-2.065420	2.467358
H	-0.010466	1.853935	-1.081794
H	0.705516	-3.378002	1.512312
H	5.368733	-0.886042	-1.504628
H	3.701143	-3.394741	0.168186
H	3.476108	1.784842	-1.697660
H	0.584910	2.445334	1.849578
H	1.422322	3.071735	0.446683
H	-1.508233	3.617557	1.124937
H	-0.709874	5.891325	1.707059
H	0.091319	4.677413	2.717822
H	0.960206	5.414912	1.362815
H	-1.362988	5.368509	-0.661645
H	-1.173519	3.753580	-1.346427
H	0.246958	4.780395	-1.108265
H	-2.169347	1.008675	-1.697074
H	-1.070312	-0.335820	-1.546375
H	-4.134113	-1.957543	0.513200
H	-3.192337	-0.238174	2.240226
H	-3.060554	-1.170665	-2.915576
H	-4.983253	-2.678637	-3.026758
H	-3.863277	-3.271941	-1.787354
H	-5.356171	-2.438393	-1.319802
H	-5.258995	-0.176701	-3.444456
H	-4.286183	0.998552	-2.552293
H	-5.575381	0.111159	-1.723132
H	-1.314507	1.261343	3.038558
H	-0.907731	-0.393657	3.539399
H	0.313744	0.621190	2.755661
H	-1.886324	-2.709297	1.975815

Conformer 5:

C	-2.705366	-2.126729	0.151022
C	-3.203999	-0.912573	0.677210
C	-2.379942	0.249506	0.594837
C	-1.102014	0.243688	-0.002371
C	-0.639515	-0.978359	-0.497221
C	-1.419099	-2.178270	-0.444211
C	-0.245125	1.502325	0.017459
C	1.223278	1.113384	-0.294786
O	0.590828	-1.118584	-1.037402
H	1.769054	2.008031	-0.617384
C	-0.946359	-3.425118	-0.994289
C	-4.503237	-0.837757	1.303143
O	-4.964498	0.209541	1.794088
O	-3.463643	-3.219394	0.219648
O	-2.815474	1.397361	1.116596
O	-1.602903	-4.483497	-0.954151
C	-0.813881	2.679732	-0.837496
C	-0.904695	4.025076	-0.087743
C	0.474499	4.572435	0.314865
C	-1.665751	5.056445	-0.934686
C	1.894077	0.568424	0.985571
C	3.223442	-0.123067	0.783863
C	3.589714	-0.532883	-0.438299
C	2.771227	-0.353049	-1.689089

C	4.075062	-0.315452	2.032482
C	3.377404	-1.250209	3.046756
C	5.505067	-0.801802	1.761752
C	1.306347	0.094795	-1.448566
C	0.701976	0.545547	-2.783722
O	2.829562	-1.518995	-2.514712
H	-0.260459	1.852804	1.058091
H	0.041032	-3.426547	-1.475622
H	-5.097735	-1.761308	1.338842
H	-2.939081	-3.964978	-0.204514
H	-3.728309	1.221272	1.498631
H	-0.199678	2.826514	-1.733150
H	-1.816031	2.418324	-1.192848
H	-1.484105	3.848098	0.828748
H	0.374598	5.517500	0.860136
H	1.023231	3.880750	0.963626
H	1.093876	4.765762	-0.570903
H	-1.767077	6.008140	-0.401116
H	-2.672552	4.702766	-1.183022
H	-1.139593	5.259337	-1.876573
H	2.023398	1.406411	1.684139
H	1.203190	-0.121387	1.489057
H	4.546575	-1.018989	-0.606422
H	3.233901	0.426666	-2.308757
H	4.149699	0.675237	2.508971
H	3.977118	-1.336931	3.959148
H	2.387921	-0.884384	3.336716
H	3.257203	-2.254469	2.624081
H	6.072265	-0.831662	2.697817
H	6.035213	-0.140540	1.068827
H	5.513714	-1.813876	1.341077
H	1.157813	1.484622	-3.111864
H	0.906319	-0.217975	-3.538078
H	-0.377256	0.694070	-2.717776
H	2.446112	-2.251252	-2.011289

Conformer 6:

C	2.933578	-1.906922	-0.317132
C	3.308456	-0.624363	-0.779848
C	2.380599	0.448634	-0.624704
C	1.118076	0.289877	-0.015983
C	0.779173	-0.995472	0.414282
C	1.666631	-2.112009	0.286369
C	0.147583	1.461644	0.043914
C	-1.273718	0.920547	0.347685
O	-0.423915	-1.280300	0.958434
H	-1.893196	1.741351	0.728395
C	1.320575	-3.426216	0.769759
C	4.585510	-0.393235	-1.413113
O	4.939263	0.718142	-1.849502
O	3.789782	-2.917825	-0.453681
O	2.698925	1.659129	-1.085986
O	2.072906	-4.414085	0.665016
C	0.617073	2.637619	0.959206
C	0.571378	4.025392	0.286674
C	-0.858253	4.462150	-0.072852
C	1.243879	5.074830	1.184836
C	-1.914467	0.393681	-0.954924
C	-3.154368	-0.451872	-0.784395
C	-3.458272	-0.975563	0.410316
C	-2.657930	-0.765674	1.668135
C	-3.990581	-0.660125	-2.040703
C	-4.861597	-1.924457	-2.022481
C	-4.858250	0.584165	-2.337231
C	-1.245805	-0.164791	1.442008
C	-0.669929	0.267850	2.795550
O	-2.592777	-1.965625	2.442681
H	0.114888	1.869629	-0.975129
H	0.344131	-3.547512	1.258121
H	5.263381	-1.253172	-1.506098
H	3.343681	-3.731555	-0.066819

H	3.618306	1.590758	-1.486320
H	0.005289	2.675849	1.867590
H	1.644339	2.451749	1.289475
H	1.152348	3.955420	-0.643151
H	-0.854538	5.441543	-0.563998
H	-1.348269	3.759384	-0.755811
H	-1.481286	4.546210	0.827350
H	1.249163	6.060066	0.705285
H	2.282385	4.803862	1.405013
H	0.713316	5.174716	2.140703
H	-2.138358	1.257299	-1.594141
H	-1.175702	-0.194324	-1.519673
H	-4.342256	-1.593248	0.546290
H	-3.191194	-0.062962	2.322208
H	-3.277703	-0.763278	-2.873526
H	-5.348846	-2.058988	-2.993962
H	-4.267454	-2.819719	-1.813213
H	-5.654039	-1.859085	-1.268471
H	-5.374073	0.473026	-3.297331
H	-4.263043	1.501697	-2.385767
H	-5.616485	0.715440	-1.557045
H	-1.208821	1.139627	3.178930
H	-0.790723	-0.551599	3.507935
H	0.389267	0.522547	2.729361
H	-2.144034	-2.634351	1.905904

Conformer 9:

C	-3.196868	-1.379048	0.210966
C	-3.328169	-0.068955	0.725870
C	-2.218886	0.821160	0.605791
C	-1.008513	0.461024	-0.026673
C	-0.907531	-0.854478	-0.490016
C	-1.986535	-1.791947	-0.401088
C	0.162985	1.435173	-0.056847
C	1.463805	0.649388	-0.371461
O	0.229693	-1.349739	-1.025954
H	2.227862	1.344015	-0.739734
C	-1.886020	-3.129529	-0.931980
C	-4.538947	0.363757	1.383456
O	-4.680960	1.499094	1.874885
O	-4.225933	-2.218391	0.311058
O	-2.308186	2.043340	1.132037
O	-2.808388	-3.964571	-0.861976
C	-0.085243	2.692500	-0.935187
C	0.888692	3.868643	-0.694501
C	0.713643	4.924451	-1.798835
C	0.710810	4.513450	0.690300
C	1.986347	0.009568	0.936550
C	3.057984	-1.040521	0.769236
C	3.274796	-1.602380	-0.427299
C	2.536038	-1.254876	-1.690347
C	3.867607	-1.450591	1.991194
C	4.808912	-0.324707	2.467716
C	2.973958	-1.942214	3.147221
C	1.254101	-0.404210	-1.478383
C	0.801584	0.132339	-2.842508
O	2.259415	-2.424844	-2.464085
H	0.264287	1.806177	0.970666
H	-0.946794	-3.411075	-1.427006
H	-5.363303	-0.360321	1.444346
H	-3.935212	-3.086095	-0.105467
H	-3.223082	2.124682	1.540592
H	-0.051174	2.423016	-1.993510
H	-1.101474	3.050674	-0.739981
H	1.921359	3.494333	-0.762068
H	1.405948	5.762134	-1.659119
H	0.895390	4.500750	-2.793171
H	-0.305490	5.330921	-1.790994
H	1.415622	5.341518	0.825210
H	0.877553	3.805615	1.508593
H	-0.303730	4.913887	0.803591

H	2.352852	0.819241	1.579565
H	1.143741	-0.433427	1.485752
H	4.047282	-2.360225	-0.549565
H	3.193548	-0.662733	-2.340930
H	4.499258	-2.292928	1.680352
H	5.435732	-0.678816	3.293592
H	5.468952	0.008794	1.660271
H	4.251074	0.545377	2.832248
H	3.591274	-2.319883	3.969523
H	2.311486	-2.751753	2.822881
H	2.351758	-1.134868	3.549748
H	1.458265	0.942916	-3.173352
H	0.853920	-0.677414	-3.574230
H	-0.222455	0.507934	-2.815015
H	1.693107	-2.999556	-1.929570

Conformer 11:

C	-2.867877	-1.946610	0.082732
C	-3.280525	-0.721315	0.654664
C	-2.380096	0.385242	0.610309
C	-1.108991	0.318596	-0.001692
C	-0.725857	-0.920979	-0.523342
C	-1.586137	-2.065871	-0.511309
C	-0.166912	1.515255	0.050238
C	1.274824	1.035628	-0.265165
O	0.500350	-1.139829	-1.047265
H	1.883116	1.892865	-0.576839
C	-1.197504	-3.323376	-1.101830
C	-4.567618	-0.584830	1.295178
O	-4.953255	0.468898	1.835279
O	-3.700272	-2.985728	0.112031
O	-2.733730	1.532666	1.190781
O	-1.925783	-4.334610	-1.099047
C	-0.654452	2.739597	-0.772884
C	0.039554	4.078701	-0.433596
C	-0.312817	5.133280	-1.495847
C	-0.321652	4.595664	0.969074
C	1.895687	0.444816	1.021649
C	3.176544	-0.336150	0.838307
C	3.531658	-0.767486	-0.379382
C	2.746455	-0.526944	-1.640510
C	3.993030	-0.590860	2.099188
C	3.218172	-1.481875	3.096694
C	5.391468	-1.170488	1.848005
C	1.310431	0.021366	-1.426246
C	0.778880	0.520610	-2.775954
O	2.737999	-1.691815	-2.469913
H	-0.164143	1.843154	1.097268
H	-0.210820	-3.376801	-1.581457
H	-5.223517	-1.466493	1.296981
H	-3.226368	-3.750994	-0.335987
H	-3.653341	1.400127	1.574925
H	-0.532802	2.544765	-1.841095
H	-1.728917	2.862918	-0.601446
H	1.129699	3.932086	-0.468514
H	0.180129	6.088672	-1.284261
H	-0.007294	4.812429	-2.498344
H	-1.394506	5.315842	-1.519625
H	0.191137	5.541399	1.177143
H	-0.044921	3.892165	1.760827
H	-1.400662	4.773597	1.050010
H	2.073143	1.272590	1.721707
H	1.153339	-0.195937	1.516983
H	4.454904	-1.318210	-0.535831
H	3.271841	0.220797	-2.249721
H	4.125994	0.390250	2.582695
H	3.796027	-1.613333	4.017797
H	2.250937	-1.051933	3.373344
H	3.037723	-2.473560	2.665877
H	5.940568	-1.242177	2.792554
H	5.975359	-0.543183	1.166800

H	5.339256	-2.179097	1.422201
H	1.257868	1.465273	-3.051419
H	1.011192	-0.222693	-3.542414
H	-0.301251	0.674105	-2.758938
H	2.296260	-2.397112	-1.975829

Conformer 12:

C	-3.075983	-1.721740	0.250490
C	-3.364294	-0.437619	0.766809
C	-2.369552	0.579281	0.650112
C	-1.123509	0.367752	0.019789
C	-0.864382	-0.925523	-0.444707
C	-1.823572	-1.985722	-0.359198
C	-0.077694	1.475666	-0.007935
C	1.309195	0.851429	-0.316686
O	0.324995	-1.280668	-0.978201
H	1.985567	1.632637	-0.682805
C	-1.562411	-3.300838	-0.891303
C	-4.619328	-0.153808	1.422332
O	-4.896852	0.955779	1.914921
O	-3.997386	-2.678399	0.347166
O	-2.606166	1.781165	1.177478
O	-2.378281	-4.240479	-0.824546
C	-0.474931	2.692179	-0.889154
C	0.360800	3.973380	-0.666388
C	0.040582	5.000836	-1.764913
C	0.138910	4.594017	0.723131
C	1.900560	0.279028	0.991753
C	3.081206	-0.649390	0.838503
C	3.369431	-1.186921	-0.353579
C	2.608657	-0.913792	-1.623004
C	3.877086	-0.921943	2.108557
C	4.654190	-2.246171	2.099452
C	4.826841	0.255234	2.426638
C	1.233904	-0.220215	-1.423128
C	0.732086	0.259954	-2.790907
O	2.478869	-2.101246	-2.408803
H	-0.025326	1.857790	1.019140
H	-0.595117	-3.467186	-1.384354
H	-5.351434	-0.971213	1.480638
H	-3.604088	-3.504506	-0.069745
H	-3.525194	1.751291	1.584231
H	-0.423356	2.422217	-1.946693
H	-1.522932	2.933525	-0.682774
H	1.428293	3.722018	-0.756876
H	0.634828	5.912787	-1.639487
H	0.247621	4.599743	-2.763673
H	-1.018145	5.287244	-1.733764
H	0.742047	5.500963	0.842825
H	0.408632	3.912499	1.536290
H	-0.913470	4.870008	0.860565
H	2.174422	1.125305	1.634591
H	1.116605	-0.257546	1.546971
H	4.210325	-1.864246	-0.478452
H	3.200403	-0.243767	-2.261120
H	3.143657	-0.976195	2.928021
H	5.112731	-2.419595	3.078711
H	4.000250	-3.094878	1.875020
H	5.462727	-2.235837	1.359922
H	5.315127	0.104141	3.395631
H	4.298607	1.213258	2.468125
H	5.607159	0.333718	1.661256
H	1.288497	1.144968	-3.114818
H	0.888227	-0.535721	-3.523195
H	-0.330072	0.508725	-2.771713
H	1.975226	-2.741805	-1.886559

Conformer 24:

C	3.242362	1.277666	0.165406
C	3.332058	-0.005239	0.752582
C	2.198031	-0.868442	0.673026

C	1.002005	-0.507620	0.014481
C	0.941411	0.783329	-0.520309
C	2.047912	1.691813	-0.476319
C	-0.195448	-1.450208	0.016002
C	-1.472128	-0.634102	-0.316711
O	-0.178231	1.284555	-1.086841
H	-2.269183	-1.314343	-0.636800
C	1.989911	3.000806	-1.079814
C	4.525924	-0.436412	1.441152
O	4.631655	-1.547045	1.994523
O	4.295499	2.090516	0.226050
O	2.250323	-2.062792	1.264435
O	2.936552	3.810760	-1.049742
C	0.043067	-2.737532	-0.821338
C	-1.069759	-3.807542	-0.767720
C	-0.698579	-4.980442	-1.690814
C	-1.344524	-4.314239	0.657722
C	-1.946610	0.087300	0.967451
C	-2.981008	1.168368	0.765496
C	-3.199175	1.678404	-0.453842
C	-2.497367	1.241087	-1.710074
C	-3.751242	1.669650	1.978872
C	-4.726050	0.606601	2.526981
C	-2.817954	2.182514	3.093619
C	-1.243455	0.355660	-1.476138
C	-0.834871	-0.266747	-2.817342
O	-2.193347	2.359627	-2.546993
H	-0.306782	-1.790303	1.053104
H	1.061916	3.282612	-1.595426
H	5.370951	0.265505	1.468574
H	4.032865	2.942287	-0.239231
H	3.160678	-2.147349	1.683090
H	0.228232	-2.481798	-1.867833
H	0.966753	-3.195241	-0.451154
H	-2.002547	-3.373509	-1.157018
H	-1.492018	-5.735826	-1.708213
H	-0.532772	-4.643974	-2.720622
H	0.221171	-5.470847	-1.348476
H	-2.097979	-5.109693	0.647718
H	-1.714671	-3.523856	1.319016
H	-0.431800	-4.723435	1.108062
H	-2.332654	-0.675664	1.654656
H	-1.079092	0.523538	1.481947
H	-3.944687	2.458556	-0.600657
H	-3.187436	0.641325	-2.318705
H	-4.355836	2.519729	1.636857
H	-5.322869	1.025350	3.344796
H	-5.413763	0.260127	1.748609
H	-4.195371	-0.265863	2.924847
H	-3.404874	2.624836	3.905884
H	-2.130854	2.948083	2.717820
H	-2.220027	1.372340	3.526124
H	-1.507693	-1.089182	-3.079723
H	-0.897348	0.496998	-3.596200
H	0.185642	-0.651326	-2.793494
H	-1.594625	2.937919	-2.053223

Table S18. Eucalyprobusal C (**1b**) DFT-geometry optimized conformers calculated at the B3LYP/6-31+G(d,p) level of theory.

Conformer 1:

C	2.527701	-1.520565	0.712922
C	2.592805	-0.890136	-0.550000
C	1.569342	0.040946	-0.902528
C	0.496221	0.370406	-0.037709
C	0.436131	-0.328387	1.169842
C	1.438633	-1.258524	1.581108
C	-0.450141	1.506461	-0.416021

C	-1.978667	1.195416	-0.321574
C	-2.592575	0.926925	1.078247
O	-0.576679	-0.144162	2.061504
C	-0.017117	2.833655	0.272895
C	1.229317	3.523644	-0.324814
C	0.970017	4.096145	-1.727380
C	1.714957	4.632506	0.623378
C	-2.340571	0.019049	-1.266182
C	-2.239114	-1.361612	-0.645578
C	-2.098203	-1.517492	0.684262
C	-1.976337	-0.364976	1.641181
C	-2.338775	-2.573223	-1.562147
C	-3.637867	-2.577363	-2.392666
C	-1.104511	-2.707014	-2.478458
H	-2.481302	2.090862	-0.707236
C	1.361489	-1.953638	2.846120
O	2.224462	-2.766465	3.229833
O	3.486661	-2.377351	1.061679
C	3.672401	-1.171737	-1.469909
O	3.767612	-0.647442	-2.594557
O	1.628360	0.634746	-2.097193
C	-2.510352	2.075467	2.083048
O	-4.013845	0.711550	0.940527
H	-0.299455	1.662119	-1.486934
H	-0.852703	3.545105	0.224343
H	0.172393	2.648135	1.334449
H	2.034409	2.780919	-0.404851
H	1.873285	4.571798	-2.125223
H	0.669915	3.323861	-2.441713
H	0.180539	4.858371	-1.694840
H	2.607756	5.128317	0.226467
H	1.964802	4.233376	1.613035
H	0.942128	5.400295	0.758231
H	-1.706022	0.063364	-2.159128
H	-3.363824	0.155547	-1.640004
H	-2.040033	-2.518106	1.108832
H	-2.464370	-0.601036	2.591085
H	-2.359803	-3.457723	-0.912298
H	-3.719132	-3.511658	-2.958116
H	-4.524363	-2.494577	-1.754187
H	-3.659855	-1.754348	-3.115292
H	-1.170250	-3.631387	-3.062340
H	-0.178125	-2.739372	-1.896867
H	-1.030938	-1.875351	-3.187335
H	0.496310	-1.740840	3.487941
H	3.244311	-2.727093	1.972552
H	4.436698	-1.888932	-1.139725
H	2.447689	0.288181	-2.564042
H	-3.146847	1.828822	2.938061
H	-1.497146	2.242680	2.442887
H	-2.890484	2.998606	1.636706
H	-4.166849	-0.142668	0.512985

Conformer 3:

C	1.964315	-2.220031	0.599979
C	2.244235	-1.593323	-0.635056
C	1.568321	-0.376252	-0.952414
C	0.637283	0.238153	-0.078674
C	0.339845	-0.454225	1.096934
C	0.995618	-1.665542	1.473492
C	0.100617	1.627972	-0.409306
C	-1.450262	1.806075	-0.340591
C	-2.148413	1.682766	1.039548
O	-0.584483	0.000680	1.987846
C	0.911177	2.721699	0.345687
C	2.324701	3.009487	-0.207458
C	2.291055	3.694979	-1.582658
C	3.112264	3.866537	0.797423
C	-2.139659	0.839994	-1.338151
C	-2.476388	-0.534806	-0.786192
C	-2.416965	-0.782142	0.535706

C	-1.974252	0.241559	1.544482
C	-2.899759	-1.578627	-1.812644
C	-2.967790	-3.015264	-1.276627
C	-4.239329	-1.196957	-2.483958
H	-1.640998	2.828709	-0.688556
C	0.683608	-2.350170	2.707648
O	1.243434	-3.405861	3.060939
O	2.602738	-3.345811	0.916866
C	3.196522	-2.162748	-1.562171
O	3.468652	-1.651682	-2.663806
O	1.829811	0.215273	-2.120881
C	-1.738274	2.706402	2.097463
O	-3.563544	1.923450	0.880470
H	0.315122	1.774912	-1.470529
H	0.341998	3.661037	0.323497
H	1.008068	2.441198	1.398843
H	2.857308	2.055192	-0.316794
H	3.307032	3.879183	-1.949081
H	1.780543	3.088277	-2.336319
H	1.780210	4.664721	-1.519728
H	4.124633	4.072444	0.432524
H	3.200886	3.366477	1.768622
H	2.617132	4.832151	0.962885
H	-1.505338	0.716186	-2.225374
H	-3.060623	1.305785	-1.711448
H	-2.654942	-1.767178	0.925590
H	-2.533508	0.127203	2.477479
H	-2.129582	-1.556115	-2.598843
H	-3.178777	-3.707800	-2.097709
H	-2.024909	-3.322497	-0.813546
H	-3.767563	-3.134003	-0.536541
H	-4.497681	-1.930153	-3.255041
H	-4.200285	-0.214965	-2.963977
H	-5.050908	-1.185543	-1.746763
H	-0.083010	-1.902710	3.354128
H	2.247692	-3.637289	1.811031
H	3.693261	-3.095077	-1.259542
H	2.508069	-0.351306	-2.598655
H	-2.437434	2.632314	2.935721
H	-0.730398	2.538190	2.471503
H	-1.806655	3.719659	1.691712
H	-3.963043	1.179380	0.408651

Conformer 4:

C	-3.169303	-0.536619	0.536231
C	-2.715044	-1.160629	-0.647147
C	-1.311591	-1.200030	-0.899809
C	-0.346785	-0.641235	-0.021598
C	-0.844377	-0.007019	1.118574
C	-2.240013	0.055950	1.425975
C	1.126519	-0.907044	-0.326641
C	2.120907	0.280246	-0.142197
C	2.288726	0.901828	1.269098
O	-0.040700	0.564393	2.056266
C	1.528235	-2.270261	0.310522
C	2.916838	-2.838104	-0.054256
C	3.148577	-4.155924	0.703966
C	3.089770	-3.051346	-1.567033
C	1.786991	1.408941	-1.154851
C	0.783504	2.436506	-0.669486
C	0.442768	2.511638	0.630127
C	0.965775	1.570157	1.677514
C	0.196483	3.407640	-1.684185
C	1.283366	4.195382	-2.442613
C	-0.756121	2.701415	-2.671660
H	3.106680	-0.109400	-0.418066
C	-2.729002	0.722958	2.611288
O	-3.938486	0.791010	2.904617
O	-4.476462	-0.501746	0.791167
C	-3.642776	-1.761187	-1.579266
O	-3.287998	-2.326369	-2.629882

O	-0.877770	-1.807806	-2.006654
C	2.797210	-0.038968	2.360280
O	3.301056	1.930482	1.216959
H	1.154353	-1.087328	-1.404102
H	1.433483	-2.223050	1.399327
H	0.777104	-2.999700	-0.021022
H	3.689614	-2.131973	0.278163
H	4.143710	-4.562877	0.492785
H	3.066799	-4.014865	1.787688
H	2.410092	-4.912065	0.409129
H	4.065712	-3.498609	-1.785960
H	3.026213	-2.115007	-2.131333
H	2.317172	-3.725638	-1.956679
H	1.427371	0.961963	-2.088907
H	2.711388	1.933121	-1.431827
H	-0.269505	3.265337	0.961222
H	1.123176	2.098532	2.622384
H	-0.401325	4.132480	-1.116469
H	0.819982	4.950058	-3.086806
H	1.959867	4.713138	-1.753653
H	1.884638	3.541890	-3.084153
H	-1.224089	3.438624	-3.332942
H	-1.551615	2.166358	-2.143998
H	-0.226101	1.982128	-3.305293
H	-1.986480	1.185324	3.274714
H	-4.587557	-0.007394	1.660078
H	-4.711104	-1.704565	-1.328316
H	-1.685889	-2.143388	-2.500900
H	3.044039	0.557493	3.243677
H	2.054471	-0.782521	2.644502
H	3.708673	-0.545239	2.031705
H	2.958067	2.691675	0.728284

Conformer 5:

C	2.409167	-1.568180	0.763339
C	2.489442	-1.054260	-0.550860
C	1.521190	-0.092196	-0.968537
C	0.486630	0.377417	-0.121108
C	0.403571	-0.212340	1.141208
C	1.355785	-1.162732	1.620084
C	-0.411304	1.515822	-0.595096
C	-1.949853	1.262852	-0.466484
C	-2.567864	1.153537	0.953126
O	-0.585757	0.106589	2.022010
C	0.044937	2.908772	-0.065710
C	1.326803	3.504509	-0.707301
C	1.215741	5.037643	-0.780985
C	2.610188	3.116518	0.047212
C	-2.353537	0.011893	-1.291900
C	-2.313919	-1.304169	-0.538090
C	-2.177138	-1.335196	0.800917
C	-1.998577	-0.100091	1.638328
C	-2.471881	-2.594088	-1.331522
C	-3.770949	-2.619312	-2.161701
C	-1.246765	-2.875370	-2.226449
H	-2.427118	2.131017	-0.937273
C	1.260244	-1.738414	2.942533
O	2.077756	-2.568576	3.384222
O	3.319106	-2.451376	1.171978
C	3.533227	-1.480421	-1.456643
O	3.641276	-1.059052	-2.622498
O	1.601217	0.399892	-2.207070
C	-2.438176	2.388065	1.844788
O	-3.996793	0.980829	0.835090
H	-0.262315	1.566812	-1.677766
H	-0.783292	3.601119	-0.260288
H	0.178624	2.887700	1.020420
H	1.406962	3.125827	-1.734936
H	2.117187	5.478430	-1.221443
H	0.358465	5.349029	-1.388699
H	1.090701	5.470652	0.219933

H	3.495111	3.523853	-0.454506
H	2.742064	2.034417	0.123498
H	2.591997	3.520213	1.067801
H	-1.712700	-0.058685	-2.178657
H	-3.368145	0.151800	-1.687374
H	-2.164877	-2.290758	1.322011
H	-2.484258	-0.220846	2.610764
H	-2.533426	-3.408288	-0.597785
H	-3.897045	-3.600245	-2.631901
H	-4.651647	-2.431819	-1.537524
H	-3.754991	-1.871993	-2.962466
H	-1.357507	-3.848087	-2.717622
H	-0.322169	-2.895257	-1.641461
H	-1.134621	-2.121351	-3.012895
H	0.423323	-1.414582	3.575387
H	3.073595	-2.704445	2.113546
H	4.256712	-2.214841	-1.076199
H	2.387863	-0.037576	-2.653411
H	-3.094044	2.254579	2.710187
H	-1.420961	2.536819	2.201769
H	-2.766146	3.283181	1.309106
H	-4.183546	0.095347	0.493046

Conformer 6:

C	-2.882657	-1.155089	0.713440
C	-2.341809	-1.796916	-0.423013
C	-0.973732	-1.560989	-0.750726
C	-0.126729	-0.712455	0.008616
C	-0.713529	-0.074052	1.103143
C	-2.077801	-0.278498	1.481744
C	1.358077	-0.687411	-0.350750
C	2.068806	0.701087	-0.349894
C	2.143659	1.502000	0.976582
O	-0.024449	0.759745	1.928548
C	2.072320	-1.848790	0.401790
C	3.539532	-2.140210	0.019658
C	4.082825	-3.279773	0.898016
C	3.705973	-2.484065	-1.469594
C	1.450907	1.606804	-1.447148
C	0.265530	2.446937	-1.013349
C	-0.035599	2.598855	0.288823
C	0.720768	1.910621	1.391028
C	-0.512358	3.115324	-2.139768
C	-1.473774	2.107525	-2.811010
C	-1.273387	4.383896	-1.727021
H	3.106103	0.507741	-0.643856
C	-2.662361	0.399048	2.616681
O	-3.846733	0.238185	2.970299
O	-4.156117	-1.373842	1.038192
C	-3.144866	-2.684255	-1.234019
O	-2.709302	-3.273980	-2.239883
O	-0.454775	-2.180925	-1.813485
C	2.885952	0.825191	2.127851
O	2.900199	2.712498	0.757281
H	1.389816	-0.979936	-1.403198
H	2.005251	-1.698350	1.483272
H	1.490892	-2.756687	0.192185
H	4.146635	-1.249652	0.230835
H	5.136114	-3.482444	0.674398
H	4.006933	-3.035821	1.963747
H	3.521650	-4.207086	0.727085
H	4.749133	-2.731457	-1.695560
H	3.418166	-1.655245	-2.124862
H	3.089850	-3.350110	-1.741209
H	1.161821	0.991637	-2.307663
H	2.218605	2.289919	-1.838559
H	-0.871658	3.221347	0.594914
H	0.791267	2.561355	2.267505
H	0.230396	3.406596	-2.898038
H	-1.949385	2.562453	-3.686418
H	-0.958403	1.202163	-3.145212

H	-2.262999	1.808175	-2.112837
H	-1.713589	4.858977	-2.609781
H	-0.614934	5.112624	-1.243069
H	-2.094913	4.157639	-1.038290
H	-2.018522	1.083745	3.184017
H	-4.344551	-0.819278	1.855933
H	-4.190357	-2.831647	-0.929605
H	-1.185417	-2.736773	-2.222546
H	3.024171	1.557741	2.928676
H	2.337700	-0.024781	2.530610
H	3.875351	0.495178	1.800445
H	2.379825	3.319835	0.212990

Conformer 7:

C	1.899435	-2.032105	0.840755
C	2.230365	-1.506086	-0.428549
C	1.543872	-0.341003	-0.885132
C	0.551630	0.319113	-0.117983
C	0.206535	-0.278866	1.095111
C	0.872658	-1.431924	1.611161
C	-0.019548	1.644383	-0.612899
C	-1.580813	1.737163	-0.648336
C	-2.355986	1.721562	0.696325
O	-0.778534	0.222002	1.891584
C	0.670767	2.882240	0.035105
C	2.110727	3.201086	-0.449317
C	2.343067	4.722335	-0.446903
C	3.194425	2.512844	0.398433
C	-2.156881	0.633220	-1.571872
C	-2.491544	-0.683078	-0.894771
C	-2.511802	-0.788869	0.446801
C	-2.152803	0.349982	1.361407
C	-2.833208	-1.834011	-1.833065
C	-1.549956	-2.454019	-2.432472
C	-3.711341	-2.928835	-1.209175
H	-1.804319	2.704236	-1.115250
C	0.512705	-2.015002	2.883850
O	1.078219	-3.019697	3.356601
O	2.546702	-3.107804	1.286923
C	3.247155	-2.123719	-1.250690
O	3.567990	-1.699554	-2.375757
O	1.859929	0.159819	-2.081959
C	-2.054394	2.864791	1.664781
O	-3.766879	1.878108	0.431131
H	0.251624	1.696098	-1.671545
H	0.037363	3.747291	-0.196338
H	0.683088	2.794260	1.126083
H	2.212640	2.849440	-1.484620
H	3.359231	4.967392	-0.775944
H	1.641261	5.236450	-1.113508
H	2.212172	5.137762	0.560696
H	4.193615	2.731428	0.005264
H	3.080055	1.426428	0.425157
H	3.158739	2.874808	1.434089
H	-1.458979	0.447415	-2.397188
H	-3.072611	0.999826	-2.057434
H	-2.775170	-1.728629	0.923608
H	-2.753313	0.310855	2.274588
H	-3.397815	-1.392971	-2.668631
H	-1.809105	-3.207053	-3.184267
H	-0.914321	-1.706512	-2.916110
H	-0.960189	-2.944802	-1.650660
H	-3.999771	-3.656753	-1.974440
H	-4.626663	-2.515823	-0.772830
H	-3.178423	-3.478225	-0.425157
H	-0.297140	-1.534614	3.448798
H	2.152069	-3.330703	2.184397
H	3.749016	-3.012113	-0.842778
H	2.576848	-0.426148	-2.472379
H	-2.811286	2.852214	2.454635
H	-1.072881	2.770485	2.125199

H	-2.120018	3.828052	1.151387
H	-4.105539	1.069709	0.021601

Conformer 8:

C	-3.108949	-0.836356	0.467831
C	-2.595489	-1.451852	-0.695564
C	-1.190872	-1.387107	-0.936141
C	-0.281727	-0.732827	-0.064623
C	-0.838905	-0.109688	1.053847
C	-2.237981	-0.149601	1.349010
C	1.211278	-0.890857	-0.348186
C	2.107587	0.375871	-0.190725
C	2.210472	1.051458	1.201510
O	-0.091253	0.550228	1.979824
C	1.711016	-2.198930	0.333755
C	3.145146	-2.664631	0.001872
C	3.470799	-3.936404	0.802980
C	3.354886	-2.908506	-1.501582
C	1.696939	1.446131	-1.236493
C	0.602008	2.401528	-0.796677
C	0.239056	2.483323	0.496385
C	0.834135	1.622492	1.575797
C	-0.019111	3.255876	-1.894963
C	-1.286716	4.014461	-1.478604
C	1.013857	4.235494	-2.498230
H	3.123708	0.056967	-0.446619
C	-2.789417	0.506450	2.512784
O	-4.003296	0.486072	2.795156
O	-4.417219	-0.898533	0.711949
C	-3.463841	-2.145433	-1.620154
O	-3.055105	-2.705967	-2.653569
O	-0.700116	-1.986116	-2.023877
C	2.781270	0.188489	2.325561
O	3.136876	2.156955	1.126053
H	1.265790	-1.099777	-1.419400
H	1.598088	-2.127972	1.419599
H	1.024474	-2.995064	0.015784
H	3.854731	-1.889354	0.320856
H	4.497978	-4.268506	0.615572
H	3.363343	-3.769942	1.880843
H	2.798998	-4.757739	0.523460
H	4.366097	-3.282988	-1.695672
H	3.225010	-1.997533	-2.095247
H	2.643563	-3.653586	-1.878789
H	1.379592	0.949879	-2.162544
H	2.582390	2.030252	-1.518941
H	-0.545915	3.165218	0.809362
H	0.937848	2.192417	2.503861
H	-0.302062	2.555666	-2.696005
H	-1.719413	4.519003	-2.348403
H	-2.047465	3.342828	-1.068656
H	-1.070024	4.784637	-0.729344
H	0.563029	4.800604	-3.320602
H	1.893310	3.721042	-2.896035
H	1.351006	4.954633	-1.742278
H	-2.092004	1.042617	3.169596
H	-4.575402	-0.392865	1.566917
H	-4.535835	-2.166817	-1.379683
H	-1.474857	-2.396057	-2.515514
H	2.963153	0.827489	3.194869
H	2.101086	-0.608487	2.621180
H	3.737540	-0.245922	2.022372
H	2.740508	2.871963	0.608838

Conformer 9:

C	2.582417	-1.465829	0.690011
C	2.617759	-0.819159	-0.565564
C	1.561438	0.081643	-0.900234
C	0.484042	0.365974	-0.024938
C	0.455051	-0.349186	1.174517
C	1.490821	-1.250637	1.567600

C	-0.496686	1.478688	-0.387305
C	-2.017935	1.146864	-0.273722
C	-2.593537	0.837493	1.127063
O	-0.559418	-0.213931	2.072850
C	-0.081765	2.815143	0.297822
C	1.132675	3.542182	-0.321379
C	0.829694	4.110825	-1.716642
C	1.604678	4.661730	0.621142
C	-2.406742	-0.008785	-1.230651
C	-2.214337	-1.400169	-0.667356
C	-2.033963	-1.592246	0.649240
C	-1.954798	-0.469079	1.636387
C	-2.287666	-2.582193	-1.622458
C	-3.615530	-2.614514	-2.405697
C	-1.083031	-2.629578	-2.585110
H	-2.535716	2.047814	-0.625570
C	1.442716	-1.966012	2.822123
O	2.335056	-2.753205	3.192733
O	3.572063	-2.293836	1.023147
C	3.699278	-1.054612	-1.495633
O	3.770360	-0.514255	-2.614622
O	1.593864	0.691075	-2.088034
C	-2.490110	1.954240	2.169763
O	-3.995890	0.585194	0.890012
H	-0.366051	1.638242	-1.460227
H	-0.936092	3.505614	0.270575
H	0.136556	2.630589	1.354296
H	1.956637	2.823114	-0.419600
H	1.711033	4.613392	-2.130279
H	0.538358	3.331835	-2.427139
H	0.019305	4.849885	-1.666531
H	2.475078	5.184044	0.208766
H	1.885309	4.267043	1.604379
H	0.813127	5.406964	0.773544
H	-1.845508	0.096334	-2.166847
H	-3.464706	0.097425	-1.496500
H	-1.919327	-2.600099	1.044178
H	-2.428750	-0.755184	2.582168
H	-2.247047	-3.488954	-1.004636
H	-3.680703	-3.533362	-2.998493
H	-4.477705	-2.582961	-1.731375
H	-3.695815	-1.769984	-3.098990
H	-1.132444	-3.531379	-3.205229
H	-0.135853	-2.647272	-2.036960
H	-1.066938	-1.767218	-3.260374
H	0.572062	-1.793594	3.468618
H	3.346403	-2.662962	1.930474
H	4.488479	-1.750608	-1.178920
H	2.419947	0.375513	-2.564670
H	-3.083571	1.678645	3.052136
H	-1.470294	2.121262	2.511645
H	-2.894486	2.888121	1.769540
H	-4.456394	0.562689	1.739472

Conformer 10:

C	2.561797	-1.491347	0.696285
C	2.610811	-0.847329	-0.560364
C	1.568150	0.067080	-0.900605
C	0.491483	0.366370	-0.029928
C	0.448022	-0.345476	1.171170
C	1.470188	-1.259558	1.569643
C	-0.475573	1.488702	-0.397135
C	-2.000029	1.164277	-0.285150
C	-2.587986	0.861102	1.117013
O	-0.566812	-0.191897	2.065780
C	-0.052890	2.820917	0.290462
C	1.173712	3.534905	-0.319797
C	0.887493	4.105777	-1.717680
C	1.650742	4.649835	0.625623
C	-2.390132	0.007292	-1.239599
C	-2.223423	-1.382833	-0.663907

C	-2.052124	-1.569519	0.654491
C	-1.963552	-0.442936	1.636514
C	-2.312840	-2.570176	-1.611401
C	-3.641811	-2.590669	-2.393219
C	-1.109669	-2.641369	-2.574287
H	-2.505490	2.070414	-0.648369
C	1.407973	-1.971764	2.825680
O	2.288623	-2.770031	3.200306
O	3.538771	-2.332137	1.034502
C	3.692814	-1.098466	-1.485664
O	3.775774	-0.560353	-2.605002
O	1.613573	0.675057	-2.088953
C	-2.484635	1.979782	2.157554
O	-3.989850	0.537187	0.961153
H	-0.339080	1.647274	-1.469503
H	-0.899711	3.520731	0.256264
H	0.154908	2.634767	1.348726
H	1.990376	2.806733	-0.411453
H	1.777290	4.598420	-2.125030
H	0.593125	3.329441	-2.429859
H	0.085004	4.853941	-1.674130
H	2.529857	5.162385	0.219581
H	1.919577	4.252753	1.611148
H	0.866283	5.403846	0.771646
H	-1.814994	0.097757	-2.168848
H	-3.442963	0.121618	-1.522442
H	-1.957307	-2.576529	1.056233
H	-2.447584	-0.712235	2.579159
H	-2.284526	-3.472766	-0.987003
H	-3.720666	-3.513737	-2.977748
H	-4.502399	-2.541075	-1.718002
H	-3.710609	-1.751586	-3.094512
H	-1.170808	-3.547931	-3.186370
H	-0.162377	-2.666219	-2.026569
H	-1.082982	-1.785453	-3.257460
H	0.537607	-1.786493	3.468918
H	3.304650	-2.696327	1.941922
H	4.471263	-1.804661	-1.164997
H	2.437353	0.347523	-2.561706
H	-3.087770	1.703497	3.029031
H	-1.463751	2.149035	2.495116
H	-2.872697	2.922550	1.753656
H	-4.493303	1.362134	0.956106

Conformer 11:

C	1.925141	-2.164593	0.633644
C	2.175933	-1.620867	-0.646526
C	1.514741	-0.410989	-1.016509
C	0.623121	0.273960	-0.153661
C	0.348615	-0.340501	1.069015
C	0.996929	-1.537023	1.501300
C	0.078747	1.637916	-0.565861
C	-1.474724	1.801244	-0.477330
C	-2.145705	1.778510	0.921776
O	-0.546850	0.183320	1.952429
C	0.874081	2.822255	0.061038
C	2.285088	3.092664	-0.526800
C	2.590164	4.600665	-0.492838
C	3.398148	2.325246	0.207373
C	-2.171162	0.753601	-1.384350
C	-2.497872	-0.573289	-0.720485
C	-2.413898	-0.717606	0.615261
C	-1.951134	0.381283	1.531614
C	-2.942832	-1.692285	-1.654547
C	-2.981859	-3.086145	-1.012821
C	-4.305799	-1.369596	-2.309591
H	-1.687235	2.791385	-0.898802
C	0.714369	-2.136568	2.785767
O	1.266138	-3.179645	3.186035
O	2.552393	-3.281829	0.999166
C	3.088597	-2.264804	-1.565084

O	3.336976	-1.826359	-2.702928
O	1.757526	0.108421	-2.222313
C	-1.722898	2.880369	1.892731
O	-3.564967	1.999087	0.770684
H	0.270263	1.710500	-1.640558
H	0.265951	3.721861	-0.093893
H	0.967607	2.697747	1.144485
H	2.287699	2.775215	-1.578111
H	3.587567	4.811139	-0.895078
H	1.863187	5.170486	-1.082770
H	2.560245	4.984158	0.535278
H	4.372746	2.515041	-0.256195
H	3.235910	1.244626	0.204811
H	3.460227	2.648097	1.254658
H	-1.546543	0.559876	-2.265903
H	-3.097972	1.184919	-1.783811
H	-2.645134	-1.669123	1.084388
H	-2.484350	0.336095	2.485467
H	-2.197527	-1.722890	-2.463999
H	-3.208522	-3.838837	-1.774673
H	-2.023430	-3.350606	-0.555264
H	-3.759714	-3.156794	-0.243807
H	-4.579993	-2.157443	-3.018844
H	-4.291052	-0.423640	-2.858485
H	-5.094716	-1.312465	-1.550269
H	-0.021080	-1.633441	3.427480
H	2.222461	-3.510131	1.920953
H	3.576109	-3.187671	-1.221389
H	2.408430	-0.500700	-2.685677
H	-2.416153	2.875012	2.739097
H	-0.713356	2.734442	2.271969
H	-1.788161	3.859737	1.410690
H	-3.967934	1.218757	0.364959

Conformer 13:

C	-2.164504	1.861607	0.835451
C	-2.448741	1.209853	-0.385261
C	-1.610966	0.127880	-0.793288
C	-0.515068	-0.332471	-0.022196
C	-0.229941	0.387706	1.140184
C	-1.040281	1.468816	1.603504
C	0.204761	-1.612826	-0.439648
C	1.764119	-1.562954	-0.493643
C	2.532343	-1.336431	0.828365
O	0.834539	0.087743	1.934275
C	-0.368922	-2.837175	0.334653
C	-1.754296	-3.339842	-0.128803
C	-1.711173	-3.980684	-1.525012
C	-2.316992	-4.335511	0.899117
C	2.247983	-0.512944	-1.523445
C	2.373543	0.901563	-0.999673
C	2.375397	1.149960	0.319480
C	2.198129	0.074894	1.348288
C	2.548077	1.980689	-2.059601
C	1.193916	2.337736	-2.713982
C	3.253037	3.252299	-1.565350
H	2.071751	-2.549271	-0.862678
C	-0.731128	2.186053	2.819161
O	-1.424239	3.128016	3.250063
O	-2.949722	2.860452	1.237680
C	-3.563893	1.620818	-1.208311
O	-3.848806	1.083029	-2.294102
O	-1.876694	-0.484546	-1.950102
C	2.341021	-2.394304	1.918965
O	3.923685	-1.350995	0.440471
H	-0.066225	-1.766263	-1.486911
H	0.338454	-3.673365	0.246288
H	-0.435242	-2.595919	1.400114
H	-2.440294	-2.483457	-0.169458
H	-2.708365	-4.319501	-1.827079
H	-1.361020	-3.282426	-2.290757

H	-1.047938	-4.855632	-1.529678
H	-3.306924	-4.696140	0.598381
H	-2.413809	-3.877617	1.890202
H	-1.661876	-5.210858	0.997815
H	1.582378	-0.527522	-2.395304
H	3.238218	-0.806129	-1.894173
H	2.497058	2.161010	0.697502
H	2.813544	0.285904	2.230191
H	3.179278	1.537938	-2.845193
H	1.345533	3.031303	-3.548221
H	0.676567	1.456018	-3.103870
H	0.533178	2.822062	-1.986491
H	3.437488	3.929071	-2.406313
H	4.214947	3.022838	-1.095855
H	2.640558	3.798633	-0.839095
H	0.159430	1.870773	3.378842
H	-2.565831	3.198852	2.102847
H	-4.178519	2.453194	-0.838287
H	-2.680476	-0.033669	-2.350259
H	3.065741	-2.215667	2.724929
H	1.350427	-2.364154	2.369139
H	2.524349	-3.394233	1.516032
H	4.468991	-1.393755	1.237314

Conformer 14:

C	-2.077731	1.951414	0.838130
C	-2.395756	1.315181	-0.382603
C	-1.610493	0.196029	-0.794483
C	-0.534867	-0.314748	-0.027201
C	-0.212979	0.389026	1.135794
C	-0.971130	1.505484	1.602761
C	0.127202	-1.624190	-0.447984
C	1.688749	-1.632754	-0.503328
C	2.474577	-1.441080	0.819263
O	0.836902	0.035826	1.927013
C	-0.492698	-2.823671	0.328768
C	-1.901628	-3.265072	-0.125902
C	-1.895719	-3.905847	-1.522786
C	-2.501241	-4.235774	0.904998
C	2.208512	-0.597170	-1.529974
C	2.406680	0.805418	-0.995342
C	2.424345	1.045356	0.325033
C	2.200888	-0.025351	1.348906
C	2.633948	1.881250	-2.048964
C	1.298894	2.312833	-2.697543
C	3.405595	3.110734	-1.547690
H	1.949909	-2.630856	-0.883029
C	-0.625534	2.205998	2.818697
O	-1.273252	3.178557	3.252022
O	-2.814298	2.985299	1.243672
C	-3.493169	1.778414	-1.201642
O	-3.806303	1.255486	-2.287000
O	-1.908124	-0.402769	-1.950915
C	2.247787	-2.494757	1.907033
O	3.888240	-1.429202	0.509980
H	-0.153716	-1.766048	-1.494303
H	0.177028	-3.690064	0.234579
H	-0.540772	-2.581553	1.394989
H	-2.549288	-2.379173	-0.161812
H	-2.908705	-4.200261	-1.818672
H	-1.520258	-3.222838	-2.290312
H	-1.271797	-4.809311	-1.532301
H	-3.507921	-4.552228	0.610251
H	-2.571670	-3.774797	1.896840
H	-1.884810	-5.139296	0.999287
H	1.533147	-0.571884	-2.394040
H	3.180213	-0.929324	-1.917058
H	2.599113	2.046287	0.708564
H	2.828313	0.146081	2.227723
H	3.239794	1.412012	-2.839241
H	1.484455	3.003633	-3.527227

H	0.735385	1.462130	-3.092545
H	0.665787	2.825646	-1.965024
H	3.624293	3.782274	-2.384658
H	4.354257	2.827567	-1.080817
H	2.823529	3.683748	-0.817035
H	0.250824	1.849102	3.375699
H	-2.412508	3.304251	2.108335
H	-4.067191	2.638135	-0.828991
H	-2.691286	0.085708	-2.348059
H	2.986222	-2.342114	2.701356
H	1.256623	-2.434175	2.352848
H	2.380016	-3.505124	1.501838
H	4.200292	-2.343433	0.480987

Conformer 16:

C	2.125584	-2.084791	0.590815
C	2.348782	-1.430854	-0.641363
C	1.577861	-0.268334	-0.947478
C	0.606803	0.267171	-0.065524
C	0.369332	-0.454851	1.106319
C	1.120182	-1.613641	1.471441
C	-0.030510	1.616686	-0.388286
C	-1.586231	1.706717	-0.296435
C	-2.247664	1.508854	1.086166
O	-0.587989	-0.085004	2.001373
C	0.720017	2.763382	0.352978
C	2.096594	3.154307	-0.229122
C	1.983073	3.835529	-1.602083
C	2.840147	4.066736	0.760432
C	-2.258351	0.736827	-1.299652
C	-2.441124	-0.682401	-0.801371
C	-2.339794	-0.967202	0.506896
C	-1.992170	0.059940	1.540929
C	-2.778349	-1.722992	-1.860676
C	-2.723761	-3.177275	-1.373098
C	-4.149507	-1.430946	-2.512677
H	-1.834824	2.727275	-0.613173
C	0.865196	-2.331113	2.699437
O	1.508779	-3.340842	3.045297
O	2.852428	-3.158657	0.898386
C	3.337330	-1.918988	-1.576442
O	3.564131	-1.382102	-2.676281
O	1.787021	0.348317	-2.113556
C	-1.865617	2.514438	2.175937
O	-3.662392	1.652041	0.831344
H	0.155790	1.774892	-1.453133
H	0.085951	3.660808	0.348344
H	0.862122	2.487389	1.402527
H	2.695032	2.241817	-0.351416
H	2.974692	4.093315	-1.990220
H	1.501915	3.192825	-2.345036
H	1.404203	4.765409	-1.526719
H	3.826734	4.346488	0.374633
H	2.986061	3.574296	1.728681
H	2.279256	4.993582	0.937912
H	-1.679749	0.720970	-2.232606
H	-3.245560	1.134948	-1.558692
H	-2.475378	-1.981433	0.870240
H	-2.544825	-0.127666	2.468538
H	-2.017717	-1.611380	-2.649054
H	-2.885073	-3.858015	-2.215291
H	-1.754738	-3.421349	-0.926208
H	-3.504351	-3.384167	-0.631841
H	-4.355604	-2.161249	-3.302455
H	-4.190468	-0.435297	-2.963256
H	-4.951259	-1.498993	-1.768557
H	0.064083	-1.952933	3.348271
H	2.523925	-3.483385	1.791149
H	3.906070	-2.811948	-1.282003
H	2.503598	-0.162261	-2.598438
H	-2.524002	2.372763	3.043809

H	-0.843342	2.389067	2.528071
H	-1.999961	3.537546	1.813832
H	-4.125348	1.723480	1.676736

Conformer 17:

C	2.080109	-2.124222	0.594515
C	2.323158	-1.476938	-0.637590
C	1.579929	-0.297776	-0.948225
C	0.617397	0.259431	-0.070882
C	0.359263	-0.454635	1.101552
C	1.082499	-1.629178	1.470907
C	0.008365	1.620475	-0.396977
C	-1.547583	1.732015	-0.307873
C	-2.222326	1.548389	1.075578
O	-0.590582	-0.059150	1.993328
C	0.774375	2.753176	0.348948
C	2.163337	3.116020	-0.221721
C	2.075397	3.797842	-1.596249
C	2.916899	4.014107	0.773330
C	-2.228561	0.767634	-1.310117
C	-2.450842	-0.642308	-0.800365
C	-2.360102	-0.921386	0.509691
C	-1.993248	0.102762	1.539619
C	-2.815529	-1.680454	-1.853390
C	-2.791841	-3.132889	-1.357669
C	-4.183150	-1.361790	-2.500480
H	-1.773479	2.756903	-0.635182
C	0.806863	-2.339016	2.699212
O	1.426597	-3.362455	3.048105
O	2.781107	-3.213909	0.906049
C	3.304582	-1.987942	-1.567832
O	3.548326	-1.457032	-2.667044
O	1.808192	0.313565	-2.113717
C	-1.831853	2.551451	2.164625
O	-3.656043	1.632497	0.897438
H	0.202426	1.775789	-1.460902
H	0.158446	3.663439	0.337677
H	0.901565	2.476086	1.400042
H	2.743982	2.191374	-0.338181
H	3.075270	4.035327	-1.976018
H	1.588008	3.163976	-2.342721
H	1.514900	4.739460	-1.526722
H	3.912225	4.273247	0.395721
H	3.044454	3.519817	1.743185
H	2.373662	4.952486	0.945255
H	-1.639028	0.728049	-2.235519
H	-3.203915	1.182241	-1.588083
H	-2.525036	-1.928878	0.879071
H	-2.553145	-0.061332	2.464333
H	-2.056209	-1.590671	-2.645818
H	-2.970386	-3.814574	-2.195627
H	-1.827251	-3.395668	-0.911820
H	-3.574779	-3.318126	-0.613296
H	-4.410497	-2.093375	-3.283238
H	-4.203286	-0.369300	-2.959667
H	-4.981881	-1.404848	-1.751398
H	0.012305	-1.942090	3.344797
H	2.441891	-3.529810	1.798147
H	3.851771	-2.893257	-1.270353
H	2.515241	-0.213636	-2.594950
H	-2.502275	2.417801	3.020325
H	-0.809626	2.418230	2.514046
H	-1.937856	3.580268	1.800037
H	-3.911091	2.564348	0.921940

Conformer 19:

C	-3.206602	-0.431857	0.501714
C	-2.749171	-1.078156	-0.668107
C	-1.343218	-1.153659	-0.897922
C	-0.377760	-0.609780	-0.010846
C	-0.877960	0.048608	1.114920

C	-2.276916	0.147969	1.399067
C	1.091445	-0.920836	-0.298511
C	2.136598	0.214723	-0.085958
C	2.275137	0.830426	1.323860
O	-0.077888	0.615435	2.058190
C	1.431744	-2.308026	0.325960
C	2.804072	-2.924780	-0.020815
C	2.964774	-4.266330	0.714093
C	3.002528	-3.113934	-1.533571
C	1.918767	1.363176	-1.105314
C	0.898493	2.400420	-0.692944
C	0.499870	2.499665	0.584832
C	0.972303	1.578055	1.666312
C	0.379499	3.364857	-1.748674
C	1.521050	4.133661	-2.443724
C	-0.519407	2.661273	-2.786495
H	3.110246	-0.229391	-0.319076
C	-2.767934	0.841974	2.567370
O	-3.979704	0.940493	2.843378
O	-4.516462	-0.363550	0.736385
C	-3.676150	-1.665532	-1.608787
O	-3.318998	-2.250698	-2.647852
O	-0.908421	-1.783995	-1.991707
C	2.673232	-0.132511	2.446210
O	3.326742	1.810310	1.183798
H	1.127539	-1.086974	-1.377983
H	1.319774	-2.272206	1.413875
H	0.660268	-3.004706	-0.027635
H	3.598061	-2.258383	0.343296
H	3.948034	-4.708399	0.518202
H	2.861209	-4.145172	1.798498
H	2.204661	-4.985982	0.385092
H	3.962718	-3.599395	-1.740604
H	2.992743	-2.164107	-2.078182
H	2.210106	-3.744961	-1.954445
H	1.645603	0.933797	-2.076422
H	2.873108	1.880069	-1.259242
H	-0.217543	3.264567	0.876921
H	1.117440	2.130596	2.601943
H	-0.243287	4.101461	-1.224139
H	1.108881	4.890199	-3.120272
H	2.161402	4.641779	-1.715125
H	2.151222	3.466230	-3.041894
H	-0.934763	3.396343	-3.484639
H	-1.353946	2.143152	-2.303776
H	0.038624	1.926665	-3.377342
H	-2.024864	1.298200	3.234310
H	-4.628767	0.143084	1.597979
H	-4.746578	-1.580050	-1.375760
H	-1.717451	-2.103414	-2.495156
H	2.901555	0.442702	3.353616
H	1.877255	-0.829392	2.704964
H	3.569782	-0.692523	2.167330
H	3.578453	2.125788	2.062134

Conformer 20:

C	-3.198986	-0.450341	0.509820
C	-2.744331	-1.094600	-0.662386
C	-1.339161	-1.165098	-0.898222
C	-0.372796	-0.618086	-0.014807
C	-0.869731	0.037393	1.114264
C	-2.267545	0.131270	1.404283
C	1.096648	-0.921138	-0.306871
C	2.130067	0.227875	-0.096947
C	2.272431	0.850192	1.314857
O	-0.066554	0.603397	2.054853
C	1.448893	-2.302997	0.321875
C	2.823984	-2.912350	-0.026913
C	2.996653	-4.249780	0.712763
C	3.019457	-3.105798	-1.539544
C	1.892201	1.372986	-1.115365

C	0.872673	2.406374	-0.689966
C	0.483830	2.501571	0.590885
C	0.968782	1.582018	1.668159
C	0.344314	3.373537	-1.738894
C	1.478903	4.152354	-2.434291
C	-0.555734	2.671866	-2.776906
H	3.104998	-0.212420	-0.342499
C	-2.756105	0.823683	2.574947
O	-3.967215	0.918132	2.854982
O	-4.507994	-0.386427	0.749964
C	-3.673017	-1.684333	-1.599774
O	-3.317861	-2.267585	-2.640709
O	-0.906365	-1.793895	-1.994021
C	2.688930	-0.105846	2.436221
O	3.254913	1.910058	1.249737
H	1.131074	-1.090509	-1.385902
H	1.339975	-2.263751	1.410000
H	0.681150	-3.006314	-0.026593
H	3.614979	-2.239351	0.332690
H	3.982157	-4.686147	0.515265
H	2.895496	-4.125120	1.796949
H	2.240354	-4.975655	0.388958
H	3.982311	-3.585444	-1.747994
H	3.000229	-2.158376	-2.088185
H	2.230259	-3.744114	-1.955231
H	1.607306	0.941538	-2.082163
H	2.839896	1.897329	-1.285460
H	-0.231633	3.265176	0.890423
H	1.123561	2.130422	2.601513
H	-0.279389	4.104200	-1.207423
H	1.060024	4.911677	-3.103535
H	2.120258	4.658256	-1.705059
H	2.108777	3.491978	-3.040789
H	-0.978856	3.409107	-3.468074
H	-1.384899	2.145736	-2.293544
H	0.003332	1.944448	-3.375739
H	-2.011996	1.282195	3.239080
H	-4.618084	0.119621	1.612468
H	-4.742831	-1.602534	-1.362633
H	-1.716615	-2.115497	-2.494305
H	2.915224	0.480364	3.333116
H	1.906873	-0.819744	2.691113
H	3.588296	-0.665704	2.154115
H	4.134232	1.514690	1.317870

Conformer 21:

C	2.455527	-1.526781	0.750422
C	2.512190	-1.002503	-0.560619
C	1.515042	-0.064113	-0.964989
C	0.474075	0.371874	-0.107641
C	0.416092	-0.228628	1.151338
C	1.397529	-1.155928	1.616775
C	-0.453074	1.491789	-0.571777
C	-1.986636	1.228464	-0.424576
C	-2.572124	1.086055	0.999164
O	-0.578297	0.052118	2.039114
C	-0.009081	2.894235	-0.053377
C	1.253765	3.509309	-0.714959
C	1.109215	5.038515	-0.808679
C	2.551571	3.159001	0.033228
C	-2.421489	-0.003162	-1.260142
C	-2.296331	-1.337082	-0.556268
C	-2.124283	-1.401980	0.773769
C	-1.988010	-0.187880	1.639453
C	-2.430013	-2.605663	-1.385684
C	-3.763940	-2.656434	-2.157718
C	-1.236505	-2.806962	-2.342231
H	-2.473081	2.106552	-0.867112
C	1.324676	-1.745854	2.933809
O	2.168305	-2.555108	3.365849
O	3.392404	-2.387189	1.147461

C	3.560285	-1.395013	-1.476133
O	3.650244	-0.963161	-2.639900
O	1.574336	0.438484	-2.200409
C	-2.419083	2.295343	1.926270
O	-3.984276	0.871979	0.783446
H	-0.321103	1.540698	-1.656694
H	-0.850641	3.573734	-0.236990
H	0.142465	2.878437	1.030739
H	1.332868	3.117965	-1.737595
H	1.997173	5.492238	-1.263226
H	0.240338	5.323007	-1.413189
H	0.983687	5.482929	0.187280
H	3.423741	3.574772	-0.483705
H	2.704629	2.081001	0.126089
H	2.535199	3.579273	1.047221
H	-1.851983	-0.020283	-2.197042
H	-3.472254	0.122183	-1.545621
H	-2.058718	-2.368470	1.270159
H	-2.465119	-0.353452	2.612005
H	-2.426300	-3.445489	-0.678411
H	-3.875796	-3.627306	-2.652524
H	-4.618542	-2.515531	-1.487772
H	-3.810466	-1.884834	-2.934118
H	-1.331765	-3.764556	-2.865830
H	-0.286554	-2.811847	-1.798630
H	-1.186981	-2.020438	-3.103049
H	0.480503	-1.453112	3.572094
H	3.159334	-2.653893	2.088213
H	4.304874	-2.113185	-1.105563
H	2.368155	0.023611	-2.655483
H	-3.034332	2.140587	2.823047
H	-1.394605	2.439824	2.264837
H	-2.768071	3.205365	1.430529
H	-4.444767	0.953625	1.629256

Conformer 23:

C	2.437463	-1.548209	0.753155
C	2.505094	-1.025601	-0.558198
C	1.520465	-0.076070	-0.966834
C	0.481798	0.371870	-0.113392
C	0.411906	-0.226345	1.146189
C	1.380977	-1.164163	1.615636
C	-0.432876	1.500069	-0.580403
C	-1.968951	1.242597	-0.433395
C	-2.564070	1.104751	0.991787
O	-0.580870	0.069672	2.030442
C	0.018537	2.898167	-0.058068
C	1.290462	3.503499	-0.710975
C	1.160754	5.034277	-0.800773
C	2.580786	3.138696	0.043172
C	-2.405428	0.010567	-1.267192
C	-2.304466	-1.320375	-0.552860
C	-2.139083	-1.380848	0.778040
C	-1.992222	-0.165341	1.639549
C	-2.455216	-2.591765	-1.375447
C	-3.790439	-2.629884	-2.146007
C	-1.265090	-2.815504	-2.331094
H	-2.444380	2.124432	-0.885908
C	1.296633	-1.752058	2.933213
O	2.129647	-2.570498	3.368308
O	3.362781	-2.419148	1.154087
C	3.551920	-1.430555	-1.469639
O	3.651126	-1.000128	-2.633243
O	1.590201	0.425635	-2.202307
C	-2.410858	2.314863	1.917688
O	-3.978742	0.826276	0.865790
H	-0.296728	1.549425	-1.664820
H	-0.816215	3.585131	-0.246770
H	0.162603	2.880436	1.027016
H	1.371412	3.114423	-1.734369
H	2.055316	5.481058	-1.249126

H	0.297959	5.328877	-1.409275
H	1.034081	5.476883	0.195844
H	3.459516	3.548064	-0.467681
H	2.723199	2.059039	0.133099
H	2.562772	3.555689	1.058465
H	-1.823690	-0.019669	-2.196215
H	-3.450959	0.141779	-1.569232
H	-2.092361	-2.345395	1.279874
H	-2.477028	-0.315576	2.607901
H	-2.462783	-3.427034	-0.662988
H	-3.916361	-3.602882	-2.633207
H	-4.641984	-2.472080	-1.475987
H	-3.826761	-1.864095	-2.928850
H	-1.372459	-3.775986	-2.847009
H	-0.314904	-2.827859	-1.787978
H	-1.206113	-2.035928	-3.098457
H	0.453726	-1.449228	3.568385
H	3.123166	-2.682171	2.094458
H	4.286852	-2.157069	-1.096085
H	2.380942	0.001131	-2.653978
H	-3.034348	2.156988	2.804222
H	-1.384898	2.461387	2.250820
H	-2.744310	3.232616	1.418845
H	-4.446972	1.667317	0.779480

Conformer 24:

C	-2.920605	-1.091213	0.698015
C	-2.380384	-1.743797	-0.432475
C	-1.006019	-1.529583	-0.748954
C	-0.152098	-0.692807	0.015085
C	-0.737901	-0.042204	1.103689
C	-2.108593	-0.225270	1.470505
C	1.335399	-0.697919	-0.336828
C	2.091158	0.665954	-0.305884
C	2.147106	1.455073	1.026345
O	-0.045192	0.784396	1.932312
C	2.012702	-1.891396	0.401683
C	3.474748	-2.219240	0.029043
C	3.972336	-3.396291	0.884945
C	3.649298	-2.530716	-1.466348
C	1.554765	1.612432	-1.408700
C	0.350165	2.442478	-1.022850
C	0.004038	2.593776	0.264425
C	0.728220	1.918592	1.388794
C	-0.380711	3.114812	-2.177011
C	-1.307356	2.111451	-2.900793
C	-1.161967	4.378155	-1.787371
H	3.129294	0.427635	-0.570966
C	-2.693138	0.467795	2.595849
O	-3.882348	0.324773	2.941526
O	-4.199827	-1.289959	1.013364
C	-3.189851	-2.620543	-1.247856
O	-2.755044	-3.220695	-2.248210
O	-0.487972	-2.161908	-1.805210
C	2.811852	0.741819	2.207169
O	2.863896	2.690246	0.795540
H	1.365239	-0.973015	-1.394013
H	1.940601	-1.757440	1.485343
H	1.409078	-2.779619	0.173008
H	4.107775	-1.353665	0.269874
H	5.022156	-3.625017	0.670027
H	3.888284	-3.177980	1.955691
H	3.386321	-4.301479	0.682573
H	4.686353	-2.807743	-1.686295
H	3.396555	-1.676866	-2.103562
H	3.007935	-3.368262	-1.766778
H	1.330043	1.029410	-2.310195
H	2.346349	2.316600	-1.695624
H	-0.839626	3.216763	0.547185
H	0.794784	2.582939	2.254826
H	0.395132	3.416194	-2.897493

H	-1.742257	2.570588	-3.795282
H	-0.775136	1.208496	-3.215340
H	-2.128327	1.805974	-2.242777
H	-1.565395	4.860573	-2.683943
H	-0.524690	5.101212	-1.268481
H	-2.011681	4.143682	-1.136256
H	-2.045151	1.148851	3.162682
H	-4.386165	-0.729106	1.827496
H	-4.240205	-2.750323	-0.952334
H	-1.224898	-2.705748	-2.219396
H	2.936914	1.457786	3.026305
H	2.220430	-0.093541	2.579493
H	3.801717	0.364595	1.924764
H	3.811472	2.505528	0.842026

Conformer 27:

C	-3.156111	-0.709931	0.444269
C	-2.648984	-1.344742	-0.711181
C	-1.240227	-1.324201	-0.935587
C	-0.319652	-0.696708	-0.056151
C	-0.870335	-0.051778	1.053810
C	-2.273962	-0.047241	1.332333
C	1.169401	-0.912133	-0.329733
C	2.134104	0.295660	-0.136636
C	2.219312	0.952487	1.258428
O	-0.116779	0.592513	1.985492
C	1.599021	-2.258559	0.328275
C	3.013621	-2.786720	0.005854
C	3.260085	-4.096852	0.772940
C	3.237860	-2.995795	-1.500654
C	1.847529	1.405288	-1.180441
C	0.743977	2.372203	-0.806949
C	0.327493	2.470794	0.465199
C	0.865854	1.616040	1.571874
C	0.190993	3.227301	-1.938906
C	-1.064940	4.033334	-1.580800
C	1.279414	4.163106	-2.513196
H	3.137151	-0.086866	-0.353815
C	-2.818179	0.633954	2.484467
O	-4.035058	0.651386	2.755061
O	-4.468425	-0.731074	0.674397
C	-3.527950	-2.013508	-1.643455
O	-3.125878	-2.590776	-2.670492
O	-0.757109	-1.942679	-2.015931
C	2.673038	0.046230	2.406425
O	3.202068	1.999452	1.102286
H	1.224609	-1.099166	-1.404961
H	1.475218	-2.206502	1.414315
H	0.880397	-3.014477	-0.014945
H	3.756559	-2.058900	0.360055
H	4.273163	-4.474148	0.594298
H	3.139088	-3.958451	1.853468
H	2.554321	-4.874696	0.455520
H	4.231244	-3.417694	-1.689592
H	3.166708	-2.061482	-2.067132
H	2.494928	-3.689916	-1.912382
H	1.612242	0.941919	-2.147449
H	2.766548	1.981911	-1.333255
H	-0.458208	3.166356	0.744697
H	0.964840	2.203395	2.492259
H	-0.085392	2.525982	-2.741740
H	-1.446079	4.543221	-2.471565
H	-1.864098	3.392947	-1.194139
H	-0.850006	4.803057	-0.830488
H	0.878725	4.736795	-3.355741
H	2.152294	3.611302	-2.873339
H	1.618760	4.872290	-1.749555
H	-2.112332	1.155834	3.143729
H	-4.620112	-0.216207	1.525008
H	-4.602744	-1.999808	-1.415229
H	-1.539281	-2.328410	-2.515444

H	2.850210	0.656421	3.302371
H	1.926345	-0.700030	2.674128
H	3.610192	-0.454126	2.148261
H	3.425983	2.349482	1.975050

Conformer 28:

C	-3.145008	-0.736432	0.450933
C	-2.638080	-1.369213	-0.705919
C	-1.230310	-1.340218	-0.935239
C	-0.311573	-0.705693	-0.059659
C	-0.861595	-0.063487	1.052368
C	-2.263933	-0.067882	1.335699
C	1.177958	-0.909055	-0.336438
C	2.125778	0.314850	-0.148078
C	2.211674	0.980916	1.247970
O	-0.107421	0.583591	1.981105
C	1.623189	-2.246552	0.328478
C	3.041602	-2.763517	0.004669
C	3.303531	-4.066476	0.778810
C	3.263722	-2.979094	-1.501266
C	1.815647	1.417005	-1.192753
C	0.710604	2.378283	-0.807374
C	0.303246	2.473804	0.467732
C	0.855945	1.624840	1.571360
C	0.146433	3.233728	-1.933880
C	-1.115939	4.025960	-1.567420
C	1.223936	4.183814	-2.505443
H	3.131295	-0.061002	-0.376883
C	-2.808420	0.610601	2.489689
O	-4.024494	0.620065	2.763861
O	-4.456298	-0.765390	0.685620
C	-3.515930	-2.044574	-1.634358
O	-3.113614	-2.620736	-2.662002
O	-0.746541	-1.957531	-2.016307
C	2.685935	0.086221	2.396562
O	3.115522	2.107286	1.163003
H	1.232867	-1.101200	-1.410778
H	1.502249	-2.189087	1.414578
H	0.911009	-3.011573	-0.007884
H	3.778977	-2.026577	0.352810
H	4.319519	-4.435381	0.599029
H	3.184376	-3.922823	1.858804
H	2.604170	-4.852704	0.468218
H	4.260611	-3.392381	-1.691051
H	3.180526	-2.049206	-2.073457
H	2.526567	-3.683085	-1.906337
H	1.569428	0.948475	-2.154546
H	2.726005	2.002558	-1.363823
H	-0.482311	3.166686	0.753632
H	0.962727	2.209922	2.489123
H	-0.125834	2.534602	-2.740061
H	-1.505679	4.535554	-2.454646
H	-1.906994	3.376010	-1.180067
H	-0.905492	4.794645	-0.814903
H	0.814521	4.760474	-3.341769
H	2.099983	3.642685	-2.874378
H	1.560349	4.889592	-1.737471
H	-2.103718	1.137199	3.146349
H	-4.607951	-0.250899	1.536686
H	-4.590034	-2.037246	-1.402641
H	-1.528179	-2.348532	-2.512683
H	2.858488	0.709045	3.280626
H	1.956640	-0.677913	2.661939
H	3.627473	-0.411480	2.136274
H	4.020711	1.778383	1.244402

Conformer 29:

C	2.051922	-2.059321	0.627741
C	2.259464	-1.497010	-0.651886
C	1.520179	-0.330333	-1.013701
C	0.592412	0.294803	-0.143653

C	0.364517	-0.340415	1.078464
C	1.090511	-1.494941	1.502329
C	-0.032010	1.626357	-0.551439
C	-1.587621	1.727458	-0.438777
C	-2.227939	1.647038	0.965886
O	-0.560589	0.117691	1.967414
C	0.717588	2.851836	0.055557
C	2.110349	3.179316	-0.547735
C	2.338123	4.701196	-0.556546
C	3.263321	2.489539	0.201810
C	-2.279615	0.682042	-1.349211
C	-2.476199	-0.687803	-0.732126
C	-2.357994	-0.865820	0.593470
C	-1.975861	0.237612	1.532106
C	-2.849543	-1.805758	-1.696439
C	-2.797087	-3.216730	-1.094594
C	-4.233756	-1.550956	-2.336402
H	-1.841045	2.719230	-0.833398
C	0.850975	-2.117624	2.784019
O	1.471272	-3.123565	3.179769
O	2.751841	-3.135058	0.986546
C	3.204973	-2.079152	-1.577900
O	3.419046	-1.622443	-2.715704
O	1.723521	0.206460	-2.219261
C	-1.820569	2.732032	1.966757
O	-3.645001	1.783484	0.720312
H	0.135666	1.702165	-1.629832
H	0.068399	3.722211	-0.101740
H	0.828277	2.742582	1.139180
H	2.125116	2.833544	-1.589565
H	3.322204	4.951059	-0.968981
H	1.581233	5.216572	-1.158879
H	2.292016	5.111185	0.460794
H	4.225017	2.709087	-0.275367
H	3.150975	1.403065	0.235981
H	3.316975	2.849172	1.237601
H	-1.710195	0.579744	-2.282341
H	-3.264333	1.069156	-1.633309
H	-2.504063	-1.844120	1.041309
H	-2.506496	0.132139	2.485168
H	-2.107914	-1.766906	-2.509402
H	-2.983861	-3.961209	-1.875351
H	-1.820331	-3.434361	-0.650880
H	-3.562344	-3.354901	-0.322037
H	-4.466157	-2.339150	-3.060509
H	-4.276803	-0.593698	-2.863474
H	-5.017218	-1.550959	-1.570049
H	0.084367	-1.667434	3.428579
H	2.441345	-3.387191	1.908745
H	3.751238	-2.970826	-1.240222
H	2.407559	-0.360265	-2.688561
H	-2.477895	2.676317	2.845128
H	-0.799854	2.612563	2.325762
H	-1.934083	3.724863	1.522782
H	-4.095348	1.923632	1.563895

Conformer 31:

C	2.051922	-2.059321	0.627741
C	2.259464	-1.497010	-0.651886
C	1.520179	-0.330333	-1.013701
C	0.592412	0.294803	-0.143653
C	0.364517	-0.340415	1.078464
C	1.090511	-1.494941	1.502329
C	-0.032010	1.626357	-0.551439
C	-1.587621	1.727458	-0.438777
C	-2.227939	1.647038	0.965886
O	-0.560589	0.117691	1.967414
C	0.717588	2.851836	0.055557
C	2.110349	3.179316	-0.547735
C	2.338123	4.701196	-0.556546
C	3.263321	2.489539	0.201810

C	-2.279615	0.682042	-1.349211
C	-2.476199	-0.687803	-0.732126
C	-2.357994	-0.865820	0.593470
C	-1.975861	0.237612	1.532106
C	-2.849543	-1.805758	-1.696439
C	-2.797087	-3.216730	-1.094594
C	-4.233756	-1.550956	-2.336402
H	-1.841045	2.719230	-0.833398
C	0.850975	-2.117624	2.784019
O	1.471272	-3.123565	3.179769
O	2.751841	-3.135058	0.986546
C	3.204973	-2.079152	-1.577900
O	3.419046	-1.622443	-2.715704
O	1.723521	0.206460	-2.219261
C	-1.820569	2.732032	1.966757
O	-3.645001	1.783484	0.720312
H	0.135666	1.702165	-1.629832
H	0.068399	3.722211	-0.101740
H	0.828277	2.742582	1.139180
H	2.125116	2.833544	-1.589565
H	3.322204	4.951059	-0.968981
H	1.581233	5.216572	-1.158879
H	2.292016	5.111185	0.460794
H	4.225017	2.709087	-0.275367
H	3.150975	1.403065	0.235981
H	3.316975	2.849172	1.237601
H	-1.710195	0.579744	-2.282341
H	-3.264333	1.069156	-1.633309
H	-2.504063	-1.844120	1.041309
H	-2.506496	0.132139	2.485168
H	-2.107914	-1.766906	-2.509402
H	-2.983861	-3.961209	-1.875351
H	-1.820331	-3.434361	-0.650880
H	-3.562344	-3.354901	-0.322037
H	-4.466157	-2.339150	-3.060509
H	-4.276803	-0.593698	-2.863474
H	-5.017218	-1.550959	-1.570049
H	0.084367	-1.667434	3.428579
H	2.441345	-3.387191	1.908745
H	3.751238	-2.970826	-1.240222
H	2.407559	-0.360265	-2.688561
H	-2.477895	2.676317	2.845128
H	-0.799854	2.612563	2.325762
H	-1.934083	3.724863	1.522782
H	-4.095348	1.923632	1.563895

Table S19. Eucalypcamal K (**2a**) DFT geometry optimized conformers calculated at the B3LYP/6-31+G(d,p) level of theory.

Conformer 7:

C	2.575236	0.858160	-0.340123
C	2.616490	-0.555269	-0.472869
C	1.348768	-1.233997	-0.513062
C	0.107486	-0.568409	-0.407879
C	0.118010	0.814264	-0.288935
C	1.337226	1.558416	-0.255978
C	-1.182269	-1.352072	-0.404745
C	-2.328772	-0.501202	0.149927
O	-1.016680	1.546489	-0.184852
C	-3.709163	-1.146592	-0.045179
C	-4.861986	-0.219267	0.282728
C	-4.672747	1.103884	0.411107
C	-3.335519	1.790591	0.309053
C	-6.246644	-0.828033	0.455790
H	-2.169502	-0.366308	1.227216
C	1.339127	2.994398	-0.162085
C	-6.691634	-1.641632	-0.775389
C	-6.342916	-1.678572	1.739634

C	3.843256	-1.358136	-0.591551
O	3.748120	-2.600794	-0.737811
O	3.704343	1.563693	-0.299899
O	1.296146	-2.553527	-0.647001
O	2.378679	3.686061	-0.123405
C	5.238011	-0.779103	-0.527673
C	5.734415	-0.501552	0.919915
C	5.840275	-1.788676	1.751011
C	7.082617	0.231824	0.862833
C	-2.313326	0.910982	-0.448421
C	-2.543118	0.973204	-1.961313
O	-2.904719	2.078535	1.644010
H	-1.415486	-1.704799	-1.419497
H	-1.058180	-2.257016	0.199209
H	-3.810859	-1.496923	-1.083273
H	-3.767771	-2.051270	0.572449
H	-5.513569	1.748990	0.657301
H	-3.448638	2.737502	-0.241951
H	-6.946631	0.009941	0.568452
H	0.364509	3.499581	-0.128535
H	-7.724636	-1.983970	-0.650455
H	-6.644639	-1.041048	-1.690360
H	-6.068025	-2.531175	-0.920156
H	-7.371599	-2.025861	1.886799
H	-6.049623	-1.099167	2.620739
H	-5.703104	-2.566840	1.688444
H	3.441818	2.537548	-0.219911
H	2.264358	-2.864592	-0.713353
H	5.892863	-1.524216	-0.991734
H	5.287636	0.147991	-1.101265
H	5.011014	0.167402	1.401029
H	6.176731	-1.559317	2.768312
H	4.882983	-2.313924	1.825055
H	6.562850	-2.484337	1.307235
H	7.448630	0.451619	1.871948
H	6.997640	1.179637	0.320674
H	7.841651	-0.380312	0.359555
H	-1.800939	0.379881	-2.502180
H	-3.538172	0.604488	-2.222088
H	-2.463264	2.009066	-2.304003
H	-2.042093	2.514516	1.585230

Conformer 8:

C	-2.551781	0.848634	-0.339110
C	-2.594804	-0.567929	-0.431948
C	-1.338886	-1.258420	-0.307785
C	-0.106401	-0.599006	-0.105154
C	-0.116779	0.785463	-0.008570
C	-1.324922	1.539750	-0.121904
C	1.178630	-1.386807	-0.028668
C	2.386523	-0.472167	-0.253999
O	1.009705	1.511995	0.185089
C	3.727548	-1.155916	0.053244
C	4.904719	-0.201659	0.063966
C	4.719849	1.127258	0.116897
C	3.373896	1.804458	0.110749
C	6.307662	-0.791387	0.021401
H	2.397822	-0.167673	-1.308150
C	-1.330506	2.973408	0.002444
C	6.561861	-1.791765	1.166161
C	6.619328	-1.432814	-1.346932
C	-3.809572	-1.363484	-0.666263
O	-3.714400	-2.611430	-0.757203
O	-3.670423	1.564279	-0.442799
O	-1.288250	-2.582239	-0.390271
O	-2.359408	3.674801	-0.097549
C	-5.194037	-0.769587	-0.790108
C	-5.859220	-0.444248	0.577664
C	-7.179156	0.302786	0.336075
C	-6.084985	-1.705294	1.424700
C	2.256689	0.828408	0.548513

C	2.242665	0.649286	2.069415
O	3.157081	2.302843	-1.214064
H	1.254056	-1.898179	0.941589
H	1.163484	-2.183282	-0.779940
H	3.668393	-1.667905	1.025414
H	3.896196	-1.951659	-0.682760
H	5.580062	1.793407	0.112958
H	3.384615	2.651950	0.814077
H	7.004813	0.046079	0.153730
H	-0.369733	3.466849	0.201498
H	7.606211	-2.122108	1.156191
H	6.361071	-1.340527	2.143988
H	5.935523	-2.685856	1.069166
H	7.663245	-1.763831	-1.380255
H	6.461633	-0.720433	-2.162847
H	5.992503	-2.311456	-1.536820
H	-3.409547	2.536733	-0.342740
H	-2.246391	-2.886971	-0.557009
H	-5.161475	0.140285	-1.391764
H	-5.797227	-1.522030	-1.309123
H	-5.189765	0.229998	1.124998
H	-7.662445	0.555545	1.286417
H	-7.014280	1.233655	-0.216879
H	-7.880100	-0.314564	-0.239835
H	-6.537891	-1.442432	2.387278
H	-5.152148	-2.240301	1.627964
H	-6.759859	-2.404358	0.915940
H	1.432685	-0.013587	2.385478
H	3.188926	0.233091	2.423521
H	2.094780	1.619087	2.553602
H	2.290474	2.734682	-1.226023

Conformer 10:

C	-2.436732	1.271618	-0.021792
C	-2.620800	-0.135375	0.014501
C	-1.430411	-0.936083	0.104630
C	-0.128368	-0.390658	0.147272
C	0.000404	0.991164	0.121392
C	-1.136456	1.851552	0.038102
C	1.078387	-1.295996	0.194826
C	2.344196	-0.530129	-0.202238
O	1.203394	1.612862	0.160358
C	3.635426	-1.318958	0.063197
C	4.894342	-0.491659	-0.101324
C	4.841200	0.848998	-0.154205
C	3.569805	1.656737	-0.118189
C	6.226568	-1.222358	-0.196807
H	2.295848	-0.315543	-1.277274
C	-0.997075	3.283972	0.036523
C	6.482005	-2.143126	1.012664
C	6.358624	-2.005272	-1.519855
C	-3.926781	-0.818883	-0.033272
O	-3.957126	-2.070062	0.006766
O	-3.489603	2.083999	-0.103404
O	-1.509355	-2.261922	0.144931
O	-1.960383	4.075078	-0.042234
C	-5.230694	-0.052039	-0.129331
C	-6.541260	-0.866435	-0.173871
C	-6.671425	-1.745031	-1.429732
C	-6.809920	-1.661259	1.115385
C	2.405795	0.838220	0.488198
C	2.502741	0.785568	2.015707
O	3.290252	2.065881	-1.461683
H	1.186930	-1.732218	1.198096
H	0.924690	-2.145086	-0.479327
H	3.609938	-1.740520	1.079242
H	3.665484	-2.185567	-0.608698
H	5.757159	1.421755	-0.283537
H	3.720267	2.552823	0.504255
H	7.008374	-0.451867	-0.196121
H	0.019608	3.692150	0.114778

H	7.485766	-2.577674	0.953146
H	6.409121	-1.592759	1.957050
H	5.767982	-2.973852	1.047265
H	7.360026	-2.442145	-1.602232
H	6.199916	-1.351949	-2.383607
H	5.637866	-2.828653	-1.579318
H	-3.133112	3.030238	-0.104552
H	-2.499700	-2.482164	0.097509
H	-5.258493	0.653065	0.710620
H	-5.164876	0.596639	-1.011617
H	-7.325949	-0.098578	-0.241843
H	-7.678286	-2.173573	-1.491156
H	-6.506157	-1.159127	-2.342093
H	-5.952317	-2.567865	-1.417215
H	-7.819147	-2.088174	1.095419
H	-6.742124	-1.016262	1.999943
H	-6.096809	-2.480759	1.234602
H	1.662583	0.236774	2.449709
H	3.431365	0.304945	2.332989
H	2.489103	1.801478	2.421364
H	2.470080	2.580389	-1.445955

Conformer 12:

C	-2.442688	1.129083	0.073956
C	-2.577242	-0.279384	0.183712
C	-1.360020	-1.035415	0.292411
C	-0.076205	-0.448012	0.260536
C	0.005399	0.933527	0.151058
C	-1.161506	1.752291	0.061021
C	1.161043	-1.310169	0.322272
C	2.386570	-0.530254	-0.163521
O	1.187580	1.593984	0.113316
C	3.711688	-1.260799	0.101710
C	4.935900	-0.405740	-0.156576
C	4.836185	0.927032	-0.285984
C	3.540365	1.695071	-0.252187
C	6.287503	-1.099120	-0.258205
H	2.295049	-0.382094	-1.246933
C	-1.070687	3.186705	-0.014231
C	6.611080	-1.946090	0.988260
C	6.401883	-1.947035	-1.542319
C	-3.858833	-1.004448	0.193695
O	-3.853862	-2.248023	0.341326
O	-3.525760	1.900913	-0.003549
O	-1.395838	-2.357727	0.418731
O	-2.062513	3.941239	-0.096530
C	-5.192965	-0.300241	0.043311
C	-6.412753	-1.230619	-0.071673
C	-7.701333	-0.428695	0.172053
C	-6.463186	-1.941757	-1.433591
C	2.425722	0.878217	0.442645
C	2.576429	0.920715	1.966146
O	3.201840	2.013751	-1.606564
H	1.316278	-1.679867	1.345884
H	1.014704	-2.203730	-0.293530
H	3.734806	-1.620715	1.141269
H	3.748006	-2.165028	-0.518308
H	5.727551	1.519490	-0.481508
H	3.681574	2.631251	0.310549
H	7.043639	-0.306187	-0.324400
H	-0.066346	3.630827	0.007927
H	7.625877	-2.352405	0.917737
H	6.550651	-1.348990	1.904727
H	5.925513	-2.795080	1.089740
H	7.413043	-2.358714	-1.634743
H	6.196289	-1.345256	-2.433194
H	5.704913	-2.792726	-1.535078
H	-3.203182	2.857129	-0.063468
H	-2.379463	-2.610019	0.423165
H	-5.297054	0.370248	0.907214
H	-5.141968	0.379707	-0.814993

H	-6.330184	-1.997414	0.707863
H	-8.582126	-1.076754	0.104712
H	-7.704136	0.038223	1.163802
H	-7.819315	0.368898	-0.572508
H	-7.330367	-2.609263	-1.490705
H	-5.566812	-2.543753	-1.605246
H	-6.552491	-1.213003	-2.249990
H	1.770849	0.372655	2.462228
H	3.531272	0.490169	2.277526
H	2.542152	1.958310	2.311176
H	2.365614	2.501800	-1.591747

Conformer 18:

C	-2.446481	1.273041	0.043625
C	-2.669373	-0.128300	0.006370
C	-1.500775	-0.964858	0.035590
C	-0.183462	-0.458258	0.087731
C	-0.016375	0.918950	0.133948
C	-1.129763	1.813232	0.114917
C	0.997885	-1.397470	0.068378
C	2.278583	-0.646069	-0.308020
O	1.203894	1.504852	0.186970
C	3.551858	-1.478821	-0.100237
C	4.834090	-0.682174	-0.252210
C	4.815200	0.660470	-0.245218
C	3.565817	1.496556	-0.133012
C	6.110357	-1.501409	-0.404380
H	2.221407	-0.374724	-1.369694
C	-0.950396	3.239261	0.188874
C	7.345157	-0.692186	-0.823291
C	6.413715	-2.313295	0.875419
C	-3.994484	-0.772317	-0.059652
O	-4.059467	-2.022545	-0.085247
O	-3.477235	2.117006	0.022421
O	-1.616305	-2.288300	0.005907
O	-1.892170	4.059407	0.167961
C	-5.277373	0.033969	-0.098243
C	-6.610559	-0.740547	-0.172338
C	-6.777237	-1.545589	-1.472431
C	-6.889190	-1.596743	1.074825
C	2.389256	0.681220	0.451509
C	2.510602	0.546658	1.972324
O	3.272555	1.989452	-1.445154
H	1.108690	-1.888907	1.045532
H	0.810730	-2.205173	-0.646877
H	3.524489	-1.948573	0.893968
H	3.555933	-2.312684	-0.815208
H	5.730867	1.226899	-0.381282
H	3.756296	2.351447	0.535009
H	5.909547	-2.229790	-1.205857
H	0.078286	3.614800	0.273603
H	8.188792	-1.366699	-1.002900
H	7.165504	-0.125161	-1.742022
H	7.650774	0.012353	-0.041093
H	7.300816	-2.938656	0.728266
H	5.587640	-2.974596	1.153846
H	6.610965	-1.641624	1.719214
H	-3.094484	3.051856	0.067105
H	-2.612966	-2.478699	-0.038169
H	-5.276934	0.692010	0.779533
H	-5.202395	0.728423	-0.944177
H	-7.374044	0.051126	-0.189989
H	-7.796537	-1.941523	-1.546280
H	-6.603382	-0.915897	-2.353488
H	-6.082091	-2.387941	-1.512052
H	-7.910094	-1.994140	1.042752
H	-6.795097	-1.002953	1.992221
H	-6.198159	-2.440737	1.141710
H	1.665286	-0.004795	2.392784
H	3.433029	0.029727	2.247863
H	2.527548	1.540335	2.429630

H	2.467100	2.523391	-1.383674

Conformer 20:

C	2.446756	1.128073	0.023494
C	2.623023	-0.275124	0.141152
C	1.429697	-1.075813	0.138910
C	0.131444	-0.533714	0.016696
C	0.010299	0.842851	-0.117110
C	1.150595	1.703032	-0.116061
C	-1.079817	-1.433067	0.060905
C	-2.344716	-0.621043	0.355409
O	-1.188606	1.461306	-0.240164
C	-3.638524	-1.425401	0.163370
C	-4.897775	-0.581825	0.231508
C	-4.835228	0.756618	0.143266
C	-3.557014	1.545395	0.014489
C	-6.203358	-1.350267	0.398842
H	-2.306152	-0.286563	1.399766
C	1.019253	3.127926	-0.270403
C	-7.421858	-0.478607	0.731194
C	-6.499352	-2.232282	-0.835479
C	3.924521	-0.952630	0.265925
O	3.952660	-2.197044	0.403354
O	3.503513	1.939119	0.037876
O	1.503433	-2.397050	0.260277
O	1.985980	3.918624	-0.263246
C	5.239229	-0.197922	0.249169
C	6.499111	-1.080707	0.256985
C	6.714405	-1.782616	-1.093594
C	7.724837	-0.231766	0.630973
C	-2.392906	0.659736	-0.486558
C	-2.479661	0.434962	-1.999016
O	-3.281616	2.109456	1.301595
H	-1.181560	-1.980321	-0.887124
H	-0.937051	-2.200297	0.828997
H	-3.600874	-1.955858	-0.799470
H	-3.687969	-2.213295	0.927112
H	-5.735225	1.358293	0.219585
H	-3.702668	2.362696	-0.709508
H	-6.047200	-2.031969	1.249809
H	0.006135	3.529793	-0.406660
H	-8.291251	-1.114151	0.929126
H	-7.247996	0.140045	1.617134
H	-7.683991	0.183665	-0.102053
H	-7.410379	-2.818667	-0.674709
H	-5.688671	-2.935776	-1.047927
H	-6.651635	-1.609988	-1.725201
H	3.153716	2.881633	-0.068377
H	2.491344	-2.612988	0.353083
H	5.247881	0.482253	-0.610481
H	5.231117	0.473557	1.118476
H	6.367633	-1.854068	1.023225
H	7.608343	-2.415716	-1.063497
H	5.864078	-2.418017	-1.355389
H	6.857143	-1.046857	-1.896034
H	8.632010	-0.845681	0.652412
H	7.609426	0.229832	1.618470
H	7.887031	0.573504	-0.096826
H	-1.641747	-0.166444	-2.361833
H	-3.410601	-0.070361	-2.267332
H	-2.453794	1.398429	-2.516580
H	-2.457756	2.613183	1.229886

Conformer 22:

C	2.446756	1.128073	0.023494
C	2.623023	-0.275124	0.141152
C	1.429697	-1.075813	0.138910
C	0.131444	-0.533714	0.016696
C	0.010299	0.842851	-0.117110
C	1.150595	1.703032	-0.116061
C	-1.079817	-1.433067	0.060905

C	-2.344716	-0.621043	0.355409
O	-1.188606	1.461306	-0.240164
C	-3.638524	-1.425401	0.163370
C	-4.897775	-0.581825	0.231508
C	-4.835228	0.756618	0.143266
C	-3.557014	1.545395	0.014489
C	-6.203358	-1.350267	0.398842
H	-2.306152	-0.286563	1.399766
C	1.019253	3.127926	-0.270403
C	-7.421858	-0.478607	0.731194
C	-6.499352	-2.232282	-0.835479
C	3.924521	-0.952630	0.265925
O	3.952660	-2.197044	0.403354
O	3.503513	1.939119	0.037876
O	1.503433	-2.397050	0.260277
O	1.985980	3.918624	-0.263246
C	5.239229	-0.197922	0.249169
C	6.499111	-1.080707	0.256985
C	6.714405	-1.782616	-1.093594
C	7.724837	-0.231766	0.630973
C	-2.392906	0.659736	-0.486558
C	-2.479661	0.434962	-1.999016
O	-3.281616	2.109456	1.301595
H	-1.181560	-1.980321	-0.887124
H	-0.937051	-2.200297	0.828997
H	-3.600874	-1.955858	-0.799470
H	-3.687969	-2.213295	0.927112
H	-5.735225	1.358293	0.219585
H	-3.702668	2.362696	-0.709508
H	-6.047200	-2.031969	1.249809
H	0.006135	3.529793	-0.406660
H	-8.291251	-1.114151	0.929126
H	-7.247996	0.140045	1.617134
H	-7.683991	0.183665	-0.102053
H	-7.410379	-2.818667	-0.674709
H	-5.688671	-2.935776	-1.047927
H	-6.651635	-1.609988	-1.725201
H	3.153716	2.881633	-0.068377
H	2.491344	-2.612988	0.353083
H	5.247881	0.482253	-0.610481
H	5.231117	0.473557	1.118476
H	6.367633	-1.854068	1.023225
H	7.608343	-2.415716	-1.063497
H	5.864078	-2.418017	-1.355389
H	6.857143	-1.046857	-1.896034
H	8.632010	-0.845681	0.652412
H	7.609426	0.229832	1.618470
H	7.887031	0.573504	-0.096826
H	-1.641747	-0.166444	-2.361833
H	-3.410601	-0.070361	-2.267332
H	-2.453794	1.398429	-2.516580
H	-2.457756	2.613183	1.229886

Conformer 23:

C	2.434246	1.122945	0.075204
C	2.571110	-0.288953	0.116794
C	1.357247	-1.055203	0.049372
C	0.076205	-0.471645	-0.062821
C	-0.006066	0.913054	-0.120611
C	1.156446	1.740161	-0.052992
C	-1.159368	-1.338078	-0.088063
C	-2.406452	-0.509175	0.233948
O	-1.185791	1.570098	-0.227632
C	-3.718027	-1.269447	-0.014430
C	-4.951115	-0.392899	0.073417
C	-4.853387	0.946048	0.048440
C	-3.554709	1.708010	-0.005982
C	-6.308988	-1.072563	0.183467
H	-2.373469	-0.232908	1.295404
C	1.065635	3.174472	-0.129388
C	-6.569156	-2.061031	-0.970208

C	-6.495242	-1.763607	1.550489
C	3.851695	-1.007827	0.225852
O	3.844406	-2.258349	0.292041
O	3.511684	1.902611	0.152149
O	1.393770	-2.382719	0.096589
O	2.052702	3.936735	-0.062222
C	5.185802	-0.289547	0.276744
C	6.421645	-1.205528	0.259986
C	6.647245	-1.836907	-1.123322
C	7.660980	-0.412735	0.705463
C	-2.406979	0.817135	-0.536667
C	-2.473825	0.677536	-2.060340
O	-3.289448	2.186628	1.317293
H	-1.260651	-1.827734	-1.067143
H	-1.049816	-2.151213	0.637071
H	-3.686069	-1.750553	-1.003498
H	-3.791567	-2.093643	0.705745
H	-5.751722	1.555243	0.123481
H	-3.661425	2.570069	-0.683105
H	-7.064278	-0.278987	0.115233
H	0.066107	3.610919	-0.259450
H	-7.588051	-2.458180	-0.906814
H	-6.457334	-1.575800	-1.945981
H	-5.882956	-2.914827	-0.934413
H	-7.511593	-2.163707	1.636570
H	-6.334922	-1.060692	2.374063
H	-5.802383	-2.602743	1.680018
H	3.188887	2.858893	0.093149
H	2.373783	-2.630587	0.193007
H	5.230226	0.436421	-0.543467
H	5.177054	0.333092	1.181661
H	6.253503	-2.016426	0.978803
H	7.523230	-2.495198	-1.110987
H	5.786168	-2.432960	-1.437305
H	6.826659	-1.061877	-1.880126
H	8.550998	-1.051610	0.710487
H	7.536614	-0.003812	1.714853
H	7.860004	0.427062	0.027516
H	-1.643902	0.077023	-2.442471
H	-3.411457	0.209665	-2.370124
H	-2.417303	1.666775	-2.524055
H	-2.452559	2.672947	1.290050

Conformer 24:

C	2.582710	0.886365	-0.304830
C	2.675651	-0.519442	-0.484185
C	1.433322	-1.239641	-0.570277
C	0.167813	-0.621144	-0.466048
C	0.127973	0.756295	-0.300186
C	1.319712	1.540266	-0.219855
C	-1.093661	-1.448520	-0.513073
C	-2.277678	-0.655999	0.048945
O	-1.033107	1.445329	-0.192065
C	-3.632911	-1.336183	-0.189031
C	-4.826393	-0.459815	0.133803
C	-4.688204	0.864414	0.304153
C	-3.368469	1.591946	0.261705
C	-6.161892	-1.186525	0.238967
H	-2.140286	-0.551111	1.132589
C	1.269695	2.971332	-0.076944
C	-6.295435	-1.883881	1.612066
C	-7.390991	-0.306740	-0.032521
C	3.931685	-1.274997	-0.607188
O	3.882785	-2.514732	-0.795221
O	3.685565	1.628556	-0.220261
O	1.429377	-2.554869	-0.749256
O	2.283589	3.696633	0.003983
C	5.303962	-0.650302	-0.500297
C	5.767024	-0.403885	0.963871
C	5.906654	-1.713586	1.753679
C	7.088269	0.379033	0.953878

C	-2.301343	0.774702	-0.502768
C	-2.503752	0.879257	-2.017294
O	-2.977669	1.841159	1.616842
H	-1.297185	-1.774093	-1.543155
H	-0.948497	-2.368748	0.062413
H	-3.711709	-1.662224	-1.238371
H	-3.678375	-2.259537	0.402297
H	-5.548984	1.484220	0.536328
H	-3.497082	2.555787	-0.255387
H	-6.148067	-1.977724	-0.526583
H	0.277524	3.441087	-0.043561
H	-7.208105	-2.488961	1.647265
H	-5.449905	-2.546154	1.823354
H	-6.347109	-1.139578	2.414168
H	-8.297186	-0.921569	-0.043696
H	-7.315458	0.205283	-0.997311
H	-7.524407	0.452882	0.745693
H	3.387734	2.589481	-0.112201
H	2.408921	-2.830020	-0.807322
H	5.992097	-1.356325	-0.977142
H	5.329981	0.296509	-1.042396
H	5.012210	0.222079	1.454472
H	6.217859	-1.506613	2.783736
H	4.968242	-2.275336	1.793508
H	6.661453	-2.367271	1.299834
H	7.430248	0.577637	1.975738
H	6.977159	1.340958	0.442159
H	7.876914	-0.188006	0.443345
H	-1.728344	0.333724	-2.561836
H	-3.478233	0.481513	-2.311000
H	-2.457849	1.928428	-2.323884
H	-2.130321	2.309378	1.595357

Conformer 26:

C	-2.442651	1.128899	0.073442
C	-2.577183	-0.279566	0.183178
C	-1.359963	-1.035536	0.292066
C	-0.076171	-0.448114	0.260486
C	0.005445	0.933469	0.151265
C	-1.161487	1.752170	0.060907
C	1.161101	-1.310241	0.322308
C	2.386590	-0.530205	-0.163372
O	1.187568	1.593993	0.114139
C	3.711751	-1.260775	0.101644
C	4.935939	-0.405642	-0.156537
C	4.836201	0.927151	-0.285720
C	3.540356	1.695128	-0.251767
C	6.287542	-1.098995	-0.258345
H	2.295003	-0.381863	-1.246755
C	-1.070749	3.186591	-0.014358
C	6.611170	-1.946225	0.987931
C	6.401882	-1.946634	-1.542643
C	-3.858829	-1.004553	0.193205
O	-3.853960	-2.248146	0.340509
O	-3.525695	1.900685	-0.004439
O	-1.395720	-2.357911	0.418270
O	-2.062580	3.941099	-0.096963
C	-5.192884	-0.300116	0.043167
C	-6.412876	-1.230300	-0.071116
C	-7.701235	-0.428150	0.173019
C	-6.464001	-1.941687	-1.432881
C	2.425801	0.878126	0.443056
C	2.576679	0.920330	1.966554
O	3.201758	2.013916	-1.606097
H	1.316277	-1.680028	1.345891
H	1.014846	-2.203746	-0.293599
H	3.734936	-1.620876	1.141136
H	3.748044	-2.164875	-0.518558
H	5.727555	1.519652	-0.481174
H	3.681555	2.631248	0.311071
H	7.043677	-0.306046	-0.324397

H	-0.066448	3.630782	0.008150
H	7.625976	-2.352497	0.917288
H	6.550755	-1.349321	1.904527
H	5.925629	-2.795256	1.089251
H	7.413032	-2.358319	-1.635175
H	6.196288	-1.344657	-2.433386
H	5.704891	-2.792310	-1.535579
H	-3.202957	2.856922	-0.064182
H	-2.379193	-2.610365	0.422601
H	-5.296491	0.370647	0.906921
H	-5.142034	0.379571	-0.815361
H	-6.330097	-1.996976	0.708520
H	-8.582159	-1.076086	0.106170
H	-7.703548	0.038944	1.164689
H	-7.819425	0.369335	-0.571626
H	-7.331304	-2.609079	-1.489506
H	-5.567789	-2.543852	-1.604800
H	-6.553534	-1.213060	-2.249367
H	1.771166	0.372148	2.462609
H	3.531573	0.489768	2.277755
H	2.542387	1.957863	2.311767
H	2.365853	2.502497	-1.591172

Conformer 28:

C	-2.439262	1.274203	-0.065107
C	-2.666933	-0.123738	0.028720
C	-1.502999	-0.955265	0.170545
C	-0.185309	-0.448255	0.206308
C	-0.013588	0.926629	0.121135
C	-1.122510	1.816539	-0.013152
C	0.992361	-1.386975	0.307813
C	2.283745	-0.677290	-0.109979
O	1.207448	1.512918	0.146936
C	3.550182	-1.487595	0.198777
C	4.837676	-0.707340	0.025427
C	4.828567	0.630752	-0.079703
C	3.577192	1.471207	-0.104065
C	6.110141	-1.545297	-0.011426
H	2.249686	-0.507099	-1.193503
C	-0.939075	3.242679	-0.074037
C	6.301935	-2.182620	-1.406610
C	7.381891	-0.794555	0.409857
C	-3.992449	-0.769162	-0.010382
O	-4.062009	-2.015675	0.086238
O	-3.465626	2.113815	-0.194441
O	-1.623299	-2.275026	0.268222
O	-1.876670	4.058467	-0.198581
C	-5.270292	0.031142	-0.165849
C	-6.603759	-0.745437	-0.205511
C	-6.733381	-1.672992	-1.425712
C	-6.924351	-1.475880	1.109694
C	2.381612	0.716645	0.522330
C	2.460066	0.727026	2.051830
O	3.328281	1.821651	-1.470228
H	1.078464	-1.780490	1.330687
H	0.819072	-2.260539	-0.329285
H	3.509682	-1.871341	1.230652
H	3.567525	-2.380707	-0.438734
H	5.756106	1.181219	-0.204827
H	3.742552	2.392613	0.476097
H	5.962352	-2.369197	0.703845
H	0.088727	3.622882	-0.000437
H	7.158939	-2.865155	-1.401487
H	5.424188	-2.754458	-1.723980
H	6.486531	-1.407048	-2.157900
H	8.227822	-1.488285	0.459632
H	7.267238	-0.326904	1.393150
H	7.646593	-0.011865	-0.309718
H	-3.080182	3.048009	-0.231224
H	-2.619159	-2.467293	0.217088
H	-5.293758	0.770949	0.643891

H	-5.166979	0.640513	-1.072190
H	-7.363088	0.041575	-0.323822
H	-7.751184	-2.074084	-1.491932
H	-6.530814	-1.131982	-2.358065
H	-6.040003	-2.515317	-1.361893
H	-7.945553	-1.873052	1.084722
H	-6.855914	-0.795805	1.967523
H	-6.239448	-2.310323	1.279788
H	1.595448	0.228820	2.498623
H	3.366704	0.227052	2.401225
H	2.479607	1.759457	2.413194
H	2.525082	2.362127	-1.491701

Conformer 29:

C	2.438513	1.121894	0.118243
C	2.619576	-0.285597	0.109673
C	1.431105	-1.086481	0.003658
C	0.133224	-0.539649	-0.100007
C	0.007967	0.843115	-0.110011
C	1.143101	1.703095	-0.000620
C	-1.074072	-1.443196	-0.167551
C	-2.349669	-0.663674	0.166136
O	-1.190791	1.466529	-0.207074
C	-3.635822	-1.450369	-0.121532
C	-4.898925	-0.617374	-0.036823
C	-4.845290	0.723719	-0.022321
C	-3.566114	1.521579	-0.020402
C	-6.200691	-1.407753	0.021961
H	-2.336692	-0.421045	1.236223
C	1.007564	3.135719	-0.025670
C	-6.449288	-1.941303	1.451304
C	-7.434329	-0.645368	-0.483327
C	3.921455	-0.967438	0.202887
O	3.953349	-2.219127	0.224520
O	3.490003	1.931519	0.234774
O	1.509193	-2.413036	0.003837
O	1.969303	3.925565	0.079489
C	5.231835	-0.209879	0.288891
C	6.496137	-1.085181	0.244297
C	6.747513	-1.655763	-1.160820
C	7.707780	-0.271302	0.726400
C	-2.382293	0.686327	-0.560939
C	-2.421045	0.595174	-2.089305
O	-3.339784	1.956511	1.325246
H	-1.149353	-1.903921	-1.162856
H	-0.946668	-2.275521	0.532495
H	-3.583274	-1.905342	-1.123450
H	-3.700651	-2.296453	0.574400
H	-5.755681	1.312227	0.039698
H	-3.684266	2.405999	-0.665973
H	-6.064386	-2.283163	-0.631759
H	-0.004084	3.545022	-0.150079
H	-7.329646	-2.593210	1.469201
H	-5.600498	-2.519427	1.830089
H	-6.624570	-1.110333	2.143247
H	-8.302694	-1.312151	-0.509250
H	-7.278919	-0.249959	-1.492387
H	-7.688986	0.192964	0.174543
H	3.137602	2.878689	0.207298
H	2.495755	-2.633318	0.099998
H	5.257077	0.548099	-0.502630
H	5.199126	0.376885	1.216955
H	6.350656	-1.927833	0.930779
H	7.643721	-2.286310	-1.168793
H	5.907007	-2.266010	-1.502142
H	6.905726	-0.847081	-1.886506
H	8.617629	-0.881447	0.711794
H	7.566258	0.094458	1.749994
H	7.882814	0.599765	0.082125
H	-1.564038	0.037990	-2.477190
H	-3.336044	0.104279	-2.429644

H	-2.393637	1.600640	-2.519599
H	-2.518867	2.470056	1.332371

Conformer 33:

C	-2.447898	1.135403	0.031665
C	-2.626901	-0.262836	0.195365
C	-1.434947	-1.049983	0.352505
C	-0.133444	-0.503122	0.316777
C	-0.008166	0.869887	0.152988
C	-1.148236	1.718876	0.012912
C	1.075855	-1.399091	0.431390
C	2.330562	-0.675404	-0.066465
O	1.193992	1.492677	0.106203
C	3.631077	-1.428996	0.244293
C	4.887090	-0.621477	-0.014457
C	4.832262	0.707801	-0.192432
C	3.554749	1.507844	-0.220859
C	6.183698	-1.421742	-0.047527
H	2.257537	-0.566776	-1.155966
C	-1.012607	3.145748	-0.118117
C	6.351711	-2.129975	-1.411231
C	7.443562	-0.610886	0.289621
C	-3.930015	-0.948232	0.214494
O	-3.965097	-2.184545	0.410997
O	-3.505725	1.935628	-0.092465
O	-1.512943	-2.364476	0.530791
O	-1.979713	3.925777	-0.244970
C	-5.239844	-0.210982	0.016264
C	-6.485435	-1.108485	-0.082053
C	-7.752655	-0.260055	0.110827
C	-6.537492	-1.869831	-1.416487
C	2.404037	0.753751	0.485156
C	2.530403	0.851783	2.008377
O	3.251797	1.774621	-1.594942
H	1.205727	-1.732096	1.470986
H	0.910931	-2.311706	-0.150853
H	3.635363	-1.755220	1.296523
H	3.656687	-2.355520	-0.343274
H	5.737514	1.277928	-0.378170
H	3.709153	2.464469	0.302561
H	6.084697	-2.208477	0.716359
H	0.004456	3.560025	-0.098905
H	7.229867	-2.784939	-1.397116
H	5.483173	-2.744943	-1.667411
H	6.487667	-1.392426	-2.209706
H	8.312189	-1.275124	0.349694
H	7.344863	-0.092410	1.248861
H	7.660730	0.137727	-0.480361
H	-3.153228	2.878541	-0.185591
H	-2.503769	-2.586825	0.529848
H	-5.336058	0.495308	0.852127
H	-5.155824	0.433345	-0.866549
H	-6.437292	-1.846745	0.727335
H	-8.651479	-0.883960	0.054471
H	-7.755744	0.244540	1.083948
H	-7.835891	0.511463	-0.665246
H	-7.423547	-2.513010	-1.461807
H	-5.657335	-2.504359	-1.550913
H	-6.592720	-1.170786	-2.261385
H	1.697791	0.351866	2.510623
H	3.464102	0.401505	2.354066
H	2.526500	1.902866	2.311826
H	2.431267	2.288311	-1.618915

Conformer 38:

C	-2.435918	1.121561	-0.119798
C	-2.623187	-0.286329	-0.109209
C	-1.437725	-1.091493	0.005328
C	-0.142507	-0.548420	0.128843
C	-0.010601	0.836090	0.146321
C	-1.140751	1.697880	0.008281

C	1.065353	-1.449424	0.204673
C	2.333998	-0.663787	-0.143519
O	1.177879	1.457525	0.280019
C	3.625664	-1.450667	0.116915
C	4.888792	-0.618023	0.028959
C	4.836657	0.725733	0.051435
C	3.557781	1.528139	0.055328
C	6.188852	-1.408672	-0.060761
H	2.289231	-0.425004	-1.215666
C	-0.991679	3.130583	-0.011626
C	6.419815	-1.919954	-1.501142
C	7.430102	-0.658319	0.443625
C	-3.925038	-0.962601	-0.211962
O	-3.962935	-2.214914	-0.239233
O	-3.482049	1.935558	-0.254819
O	-1.520572	-2.419380	-0.000360
O	-1.951981	3.921067	-0.130786
C	-5.232677	-0.199660	-0.299950
C	-6.500446	-1.069545	-0.251014
C	-6.751173	-1.636262	1.155780
C	-7.709979	-0.251990	-0.732222
C	2.372354	0.685221	0.589445
C	2.445270	0.570633	2.118142
O	3.303420	2.071552	-1.247562
H	1.152341	-1.898592	1.204221
H	0.939476	-2.289848	-0.486009
H	3.586130	-1.911188	1.116485
H	3.682492	-2.293460	-0.583725
H	5.749952	1.312574	0.004620
H	3.662940	2.407609	0.701050
H	6.056935	-2.294745	0.578970
H	0.025296	3.533307	0.078517
H	7.293527	-2.579582	-1.538466
H	5.562697	-2.484007	-1.882643
H	6.600733	-1.079361	-2.180578
H	8.296738	-1.327632	0.447389
H	7.287680	-0.281245	1.461432
H	7.680042	0.191322	-0.201541
H	-3.121109	2.881509	-0.236118
H	-2.507244	-2.634524	-0.105911
H	-5.254602	0.561965	0.488055
H	-5.198254	0.382834	-1.230620
H	-6.359758	-1.914332	-0.935876
H	-7.650055	-2.263070	1.167284
H	-5.912209	-2.249212	1.496100
H	-6.904092	-0.825440	1.880246
H	-8.622367	-0.858391	-0.714548
H	-7.568986	0.111270	-1.756797
H	-7.880113	0.621178	-0.089449
H	1.596146	0.007454	2.514159
H	3.367523	0.079832	2.438858
H	2.420173	1.571407	2.559213
H	3.640888	1.451938	-1.907949

Conformer 43:

C	-2.567042	0.878220	-0.297977
C	-2.656180	-0.530094	-0.458604
C	-1.420134	-1.263471	-0.395793
C	-0.163785	-0.652501	-0.187778
C	-0.129008	0.725232	-0.023844
C	-1.315012	1.520392	-0.074359
C	1.097013	-1.482063	-0.176246
C	2.328734	-0.594734	-0.381036
O	1.023241	1.407046	0.180526
C	3.652814	-1.329686	-0.127037
C	4.863881	-0.416124	-0.104173
C	4.720410	0.914566	0.004297
C	3.394059	1.628118	0.069816
C	6.218473	-1.107250	-0.206466
H	2.332007	-0.241162	-1.419795
C	-1.273128	2.945713	0.119489

C	7.403135	-0.162690	-0.450199
C	6.490958	-1.996828	1.027860
C	-3.899825	-1.276100	-0.704156
O	-3.845550	-2.520217	-0.858787
O	-3.664687	1.631552	-0.342414
O	-1.412623	-2.582485	-0.543998
O	-2.281325	3.682058	0.076209
C	-5.267490	-0.635485	-0.766876
C	-5.892574	-0.357916	0.630009
C	-7.194529	0.437703	0.455179
C	-6.137617	-1.651473	1.420800
C	2.254754	0.668692	0.484541
C	2.264828	0.417746	1.995335
O	3.163398	2.202469	-1.221544
H	1.174430	-2.042669	0.766277
H	1.042564	-2.239693	-0.964920
H	3.589136	-1.882330	0.821849
H	3.791550	-2.096757	-0.900884
H	5.587748	1.566788	-0.006800
H	3.450076	2.437084	0.815334
H	6.151335	-1.778806	-1.076946
H	-0.293497	3.399396	0.321258
H	8.317118	-0.744899	-0.607070
H	7.246843	0.463775	-1.333925
H	7.578253	0.495494	0.408720
H	7.441385	-2.528623	0.911468
H	5.710161	-2.748223	1.179545
H	6.555737	-1.385507	1.935682
H	-3.371726	2.589559	-0.200971
H	-2.383014	-2.849231	-0.704906
H	-5.219686	0.301471	-1.324328
H	-5.904890	-1.343080	-1.307642
H	-5.191464	0.269381	1.193297
H	-7.649094	0.657929	1.427534
H	-7.014030	1.389115	-0.056383
H	-7.926069	-0.130574	-0.133074
H	-6.561946	-1.422501	2.404825
H	-5.217017	-2.222254	1.577189
H	-6.843653	-2.305489	0.894726
H	1.443345	-0.237904	2.296511
H	3.206076	-0.039155	2.310429
H	2.152520	1.367189	2.527079
H	2.309753	2.658494	-1.190709

Conformer 46:

C	-2.584791	0.844651	0.394149
C	-2.671407	-0.572604	0.426728
C	-1.426501	-1.293089	0.401081
C	-0.164993	-0.662159	0.327091
C	-0.131077	0.725110	0.306896
C	-1.325253	1.508716	0.343078
C	1.097903	-1.485093	0.252079
C	2.266581	-0.634269	-0.254479
O	1.025946	1.426054	0.239990
C	3.627542	-1.331727	-0.116039
C	4.810149	-0.424928	-0.400583
C	4.659984	0.908710	-0.444927
C	3.346544	1.626814	-0.266480
C	6.145591	-1.125596	-0.621538
H	2.103184	-0.419575	-1.318096
C	-1.280483	2.947042	0.350807
C	7.260379	-0.223042	-1.167127
C	6.622096	-1.845829	0.660380
C	-3.922716	-1.341822	0.504799
O	-3.867268	-2.594217	0.560382
O	-3.690246	1.587366	0.419282
O	-1.416223	-2.619831	0.439891
O	-2.296798	3.673032	0.375290
C	-5.298010	-0.714736	0.502283
C	-5.801796	-0.318154	-0.914707
C	-5.961307	-1.538526	-1.833355

C	-7.123423	0.453587	-0.786743
C	2.303341	0.731701	0.441282
C	2.551534	0.680250	1.951653
O	2.908002	2.027908	-1.569385
H	1.329246	-1.916684	1.236336
H	0.937743	-2.340104	-0.413012
H	3.721385	-1.755165	0.894767
H	3.659643	-2.193848	-0.795906
H	5.502678	1.552515	-0.675555
H	3.502037	2.526167	0.350251
H	5.957646	-1.905531	-1.376279
H	-0.290042	3.421522	0.339646
H	8.150070	-0.822204	-1.387136
H	6.957793	0.282193	-2.089644
H	7.553439	0.541955	-0.438759
H	7.552899	-2.389993	0.467631
H	5.888274	-2.569372	1.028108
H	6.814933	-1.120693	1.459775
H	-3.396364	2.555402	0.404959
H	-2.393420	-2.903605	0.496868
H	-5.971874	-1.469560	0.921255
H	-5.309845	0.169956	1.141156
H	-5.061651	0.359012	-1.357354
H	-6.300702	-1.226165	-2.827318
H	-5.023728	-2.089324	-1.957180
H	-6.702690	-2.239129	-1.430236
H	-7.493894	0.756089	-1.772552
H	-6.999618	1.357366	-0.180660
H	-7.896732	-0.167079	-0.316738
H	1.798471	0.071485	2.459296
H	3.538259	0.266136	2.172664
H	2.505742	1.691250	2.367082
H	2.060473	2.484451	-1.464940

Conformer 54:

C	2.554908	0.846331	0.372260
C	2.647816	-0.570599	0.400396
C	1.418716	-1.298580	0.230280
C	0.165180	-0.674556	0.045478
C	0.126510	0.712574	0.014441
C	1.306005	1.502915	0.174495
C	-1.090086	-1.503120	-0.080209
C	-2.330241	-0.642831	0.179710
O	-1.023677	1.407338	-0.155063
C	-3.646493	-1.352591	-0.166311
C	-4.858189	-0.442495	-0.153832
C	-4.722216	0.892859	-0.148577
C	-3.397700	1.610392	-0.088105
C	-6.207814	-1.150496	-0.154743
H	-2.356561	-0.388181	1.246768
C	1.261184	2.940112	0.116731
C	-6.561021	-1.650480	1.264684
C	-7.364712	-0.319808	-0.729179
C	3.887925	-1.332666	0.612154
O	3.836831	-2.586110	0.644109
O	3.646339	1.595467	0.520239
O	1.415091	-2.625757	0.250237
O	2.263396	3.672120	0.259365
C	5.248730	-0.696587	0.780683
C	5.917162	-0.285236	-0.562049
C	7.206719	0.495902	-0.269455
C	6.197658	-1.496934	-1.463024
C	-2.242678	0.697338	-0.560706
C	-2.211211	0.590029	-2.088165
O	-3.212731	2.045816	1.263673
H	-1.141195	-1.968791	-1.074725
H	-1.051434	-2.334196	0.631690
H	-3.571548	-1.821998	-1.160145
H	-3.798642	-2.184569	0.532959
H	-5.596286	1.536974	-0.138456
H	-3.427873	2.492956	-0.746194

H	-6.093252	-2.040401	-0.792872
H	0.285002	3.407865	-0.068909
H	-7.479952	-2.246658	1.243233
H	-5.770071	-2.275179	1.691638
H	-6.719157	-0.801980	1.939261
H	-8.270123	-0.932457	-0.794130
H	-7.134121	0.053695	-1.732185
H	-7.600329	0.540011	-0.092282
H	3.351780	2.561717	0.462496
H	2.381981	-2.903423	0.413757
H	5.176853	0.182862	1.422804
H	5.872664	-1.450390	1.272498
H	5.230186	0.389587	-1.086487
H	7.691287	0.809294	-1.200904
H	7.002457	1.393761	0.323507
H	7.922793	-0.122420	0.286463
H	6.652231	-1.173800	-2.406269
H	5.286948	-2.054335	-1.703130
H	6.891040	-2.194744	-0.977974
H	-1.372243	-0.023362	-2.427703
H	-3.136984	0.152146	-2.469277
H	-2.100517	1.586598	-2.525742
H	-2.363848	2.509597	1.308097

Conformer 60:

C	2.445107	1.127202	0.024609
C	2.626935	-0.276826	0.138553
C	1.436179	-1.082125	0.125581
C	0.140480	-0.545032	-0.017362
C	0.013041	0.832830	-0.157203
C	1.149303	1.697083	-0.124519
C	-1.072122	-1.441816	0.016274
C	-2.328654	-0.621808	0.327082
O	-1.175936	1.446498	-0.318438
C	-3.628335	-1.422822	0.164844
C	-4.886061	-0.577933	0.241048
C	-4.825222	0.760320	0.115523
C	-3.547431	1.552100	-0.023310
C	-6.189221	-1.342761	0.442422
H	-2.256059	-0.292689	1.373519
C	1.005865	3.126574	-0.230389
C	-7.404112	-0.466311	0.775608
C	-6.501249	-2.245157	-0.773439
C	3.928242	-0.948454	0.273754
O	3.961952	-2.193095	0.415513
O	3.497153	1.943983	0.059964
O	1.514146	-2.404412	0.251206
O	1.971699	3.918986	-0.206458
C	5.240455	-0.188360	0.261591
C	6.503498	-1.066520	0.266689
C	6.720789	-1.763953	-1.085876
C	7.726508	-0.214599	0.642702
C	-2.383023	0.658292	-0.519566
C	-2.506835	0.411238	-2.029137
O	-3.242817	2.222658	1.207274
H	-1.186697	-1.975318	-0.938024
H	-0.931640	-2.219970	0.773795
H	-3.606863	-1.957116	-0.795958
H	-3.667949	-2.208116	0.931813
H	-5.728149	1.360542	0.179583
H	-3.681501	2.364098	-0.747411
H	-6.021108	-2.011058	1.301731
H	-0.011599	3.525489	-0.331266
H	-8.269603	-1.099215	0.996852
H	-7.221320	0.168814	1.648326
H	-7.678906	0.180594	-0.065389
H	-7.410779	-2.827158	-0.590787
H	-5.694166	-2.953195	-0.983581
H	-6.663923	-1.637864	-1.671474
H	3.139245	2.886395	-0.036043
H	2.501959	-2.614778	0.354470

H	5.247691	0.496209	-0.594507
H	5.229116	0.478698	1.134196
H	6.374836	-1.842628	1.030618
H	7.616959	-2.394134	-1.058177
H	5.872327	-2.401439	-1.348753
H	6.860341	-1.025492	-1.886420
H	8.635948	-0.825352	0.662050
H	7.609697	0.243793	1.631544
H	7.885540	0.593478	-0.082696
H	-1.679450	-0.199079	-2.400638
H	-3.446291	-0.089234	-2.276562
H	-2.481468	1.369244	-2.556609
H	-3.530576	1.662551	1.940426

Conformer 62:

C	2.579481	0.888427	-0.304324
C	2.679108	-0.517807	-0.486416
C	1.439127	-1.242033	-0.582238
C	0.173688	-0.625394	-0.499912
C	0.125715	0.755015	-0.340663
C	1.315206	1.538061	-0.230010
C	-1.088610	-1.449722	-0.555494
C	-2.263583	-0.654257	0.022558
O	-1.029384	1.445590	-0.271769
C	-3.621414	-1.338771	-0.185255
C	-4.814557	-0.463901	0.142952
C	-4.682043	0.867792	0.277105
C	-3.363566	1.601537	0.226535
C	-6.144440	-1.195702	0.281282
H	-2.092680	-0.554836	1.103968
C	1.255802	2.964669	-0.040597
C	-6.254373	-1.873488	1.666211
C	-7.382915	-0.328343	0.012875
C	3.936726	-1.268543	-0.598400
O	3.895242	-2.510523	-0.779682
O	3.678816	1.633537	-0.199237
O	1.441359	-2.559612	-0.755336
O	2.269355	3.689547	0.055682
C	5.306357	-0.637316	-0.490340
C	5.769041	-0.393254	0.974151
C	5.917829	-1.704570	1.759593
C	7.085156	0.398296	0.966362
C	-2.295548	0.776408	-0.534592
C	-2.536051	0.859190	-2.048082
O	-2.939976	1.962959	1.548501
H	-1.303988	-1.761395	-1.587389
H	-0.945754	-2.377338	0.008684
H	-3.715013	-1.670417	-1.231218
H	-3.656425	-2.259671	0.410616
H	-5.547343	1.487429	0.496131
H	-3.481467	2.559604	-0.292783
H	-6.135380	-1.998944	-0.471359
H	0.261333	3.423969	0.025611
H	-7.158811	-2.488983	1.721172
H	-5.398886	-2.522645	1.878582
H	-6.310161	-1.117934	2.458036
H	-8.284884	-0.949103	0.025243
H	-7.324933	0.167175	-0.961547
H	-7.510910	0.443895	0.779598
H	3.372820	2.592562	-0.081621
H	2.422889	-2.830318	-0.801971
H	5.997880	-1.338455	-0.969708
H	5.327599	0.311504	-1.029057
H	5.010444	0.226193	1.467088
H	6.226800	-1.498816	2.790651
H	4.983541	-2.273300	1.796247
H	6.677771	-2.351126	1.303986
H	7.426798	0.595158	1.988734
H	6.967054	1.361490	0.458634
H	7.877120	-0.161470	0.452796
H	-1.770277	0.309537	-2.601713

H	-3.515917	0.458909	-2.320046
H	-2.494876	1.905187	-2.365756
H	-3.244454	1.282386	2.163332

Conformer 64:

C	2.431648	1.122590	0.077976
C	2.574672	-0.290011	0.115477
C	1.363655	-1.060920	0.038041
C	0.085048	-0.481615	-0.093716
C	-0.003840	0.904852	-0.157088
C	1.153932	1.734846	-0.058821
C	-1.151194	-1.345508	-0.129207
C	-2.390718	-0.510578	0.208793
O	-1.173653	1.558501	-0.300889
C	-3.707770	-1.269492	-0.012099
C	-4.940225	-0.392942	0.082246
C	-4.844094	0.947746	0.022129
C	-3.546008	1.714067	-0.040347
C	-6.296263	-1.071581	0.220534
H	-2.325534	-0.240893	1.272554
C	1.049966	3.171361	-0.087267
C	-6.569303	-2.072442	-0.919725
C	-6.465044	-1.749971	1.596047
C	3.855381	-1.003145	0.233031
O	3.854070	-2.254438	0.302980
O	3.504019	1.907313	0.175017
O	1.405093	-2.389700	0.089020
O	2.035739	3.934676	-0.004383
C	5.186932	-0.279513	0.287259
C	6.426360	-1.190395	0.265062
C	6.651853	-1.816021	-1.120871
C	7.663580	-0.394554	0.711005
C	-2.397159	0.815853	-0.565935
C	-2.499922	0.655715	-2.088752
O	-3.248849	2.300048	1.234436
H	-1.264038	-1.821269	-1.113784
H	-1.043690	-2.168513	0.585108
H	-3.690385	-1.753856	-0.999560
H	-3.772158	-2.091764	0.711110
H	-5.745431	1.555427	0.084650
H	-3.641126	2.570617	-0.717689
H	-7.053300	-0.279601	0.153198
H	0.045229	3.602712	-0.182045
H	-7.587643	-2.467759	-0.840387
H	-6.468262	-1.597615	-1.901572
H	-5.883170	-2.926026	-0.882246
H	-7.477466	-2.156362	1.695556
H	-6.305360	-1.038232	2.412680
H	-5.764993	-2.582492	1.728522
H	3.172657	2.862984	0.126458
H	2.385850	-2.631835	0.194638
H	5.228516	0.451575	-0.528442
H	5.176070	0.337669	1.195852
H	6.262664	-2.004630	0.981131
H	7.530558	-2.470864	-1.112814
H	5.792310	-2.414277	-1.434926
H	6.826382	-1.037602	-1.875368
H	8.556210	-1.029920	0.712124
H	7.539404	0.010445	1.722013
H	7.857967	0.448510	0.035746
H	-1.679558	0.048140	-2.479780
H	-3.445490	0.190875	-2.378774
H	-2.444515	1.640398	-2.562026
H	-3.581962	1.712903	1.925999

Conformer 71:

C	2.440170	1.129208	-0.072318
C	2.580523	-0.279475	-0.187379
C	1.365609	-1.038665	-0.307154
C	0.083122	-0.453179	-0.295254
C	-0.005608	0.930662	-0.189342

C	1.158562	1.748590	-0.068037
C	-1.155237	-1.311677	-0.367794
C	-2.372058	-0.528687	0.136266
O	-1.180025	1.591915	-0.188028
C	-3.701243	-1.261099	-0.101009
C	-4.924402	-0.407465	0.166282
C	-4.828348	0.931064	0.263218
C	-3.533621	1.704574	0.222147
C	-6.272355	-1.103698	0.296102
H	-2.247165	-0.388819	1.219509
C	1.058069	3.180277	0.055938
C	-6.609014	-1.962832	-0.938802
C	-6.367344	-1.941955	1.588031
C	3.862463	-1.000209	-0.187962
O	3.863622	-2.245171	-0.331558
O	3.519502	1.904140	0.026296
O	1.406357	-2.362727	-0.430536
O	2.049644	3.934159	0.154274
C	5.193996	-0.290988	-0.034023
C	6.417492	-1.216574	0.079534
C	7.703069	-0.409445	-0.163027
C	6.471053	-1.929495	1.440413
C	-2.418361	0.881248	-0.471491
C	-2.606970	0.905305	-1.994339
O	-3.164379	2.133030	1.540008
H	-1.322830	-1.665167	-1.395096
H	-1.011140	-2.214781	0.234550
H	-3.739777	-1.622928	-1.139088
H	-3.727387	-2.164708	0.520361
H	-5.723286	1.523024	0.448289
H	-3.663886	2.636570	-0.339897
H	-7.030346	-0.312773	0.365005
H	0.050863	3.616088	0.068133
H	-7.622171	-2.369693	-0.851914
H	-6.560807	-1.373969	-1.861093
H	-5.923212	-2.811403	-1.040558
H	-7.373619	-2.362001	1.693088
H	-6.162184	-1.331834	2.473819
H	-5.662690	-2.781103	1.582211
H	3.188990	2.858658	0.097377
H	2.391090	-2.610788	-0.424406
H	5.296623	0.383116	-0.895214
H	5.139131	0.385854	0.826448
H	6.337959	-1.982813	-0.700916
H	8.586552	-1.054027	-0.096326
H	7.704068	0.058757	-1.154187
H	7.817682	0.387848	0.582403
H	7.340860	-2.593792	1.496600
H	5.576753	-2.534847	1.610946
H	6.557542	-1.201429	2.257767
H	-1.811391	0.352953	-2.501330
H	-3.568493	0.474754	-2.284860
H	-2.575897	1.940095	-2.347768
H	-3.456994	1.463472	2.172504

Conformer 72:

C	-2.445119	1.135436	0.031009
C	-2.630066	-0.262741	0.199008
C	-1.440717	-1.052659	0.367025
C	-0.140825	-0.506971	0.351545
C	-0.008323	0.868341	0.192189
C	-1.145221	1.715356	0.021669
C	1.069412	-1.399186	0.476051
C	2.315929	-0.672682	-0.039609
O	1.185692	1.493529	0.181718
C	3.620708	-1.429514	0.243439
C	4.876204	-0.623666	-0.023055
C	4.824897	0.712458	-0.168076
C	3.548195	1.518084	-0.190262
C	6.168958	-1.428563	-0.088045
H	2.209537	-0.570400	-1.128996

C	-0.999376	3.137395	-0.156383
C	6.315529	-2.117866	-1.463837
C	7.437380	-0.629854	0.245662
C	-3.933295	-0.944121	0.207914
O	-3.974626	-2.181821	0.400217
O	-3.498801	1.937916	-0.113917
O	-1.523724	-2.368949	0.541662
O	-1.966016	3.916190	-0.298859
C	-5.240190	-0.202021	0.005322
C	-6.489327	-1.094509	-0.092858
C	-7.753454	-0.240978	0.097986
C	-6.543733	-1.857258	-1.426409
C	2.396674	0.757388	0.514437
C	2.560208	0.835174	2.038468
O	3.217115	1.895597	-1.533821
H	1.211887	-1.717207	1.518609
H	0.906108	-2.320312	-0.093199
H	3.639680	-1.759097	1.294052
H	3.636773	-2.354740	-0.346550
H	5.733571	1.282152	-0.342547
H	3.691071	2.470971	0.332293
H	6.074784	-2.226558	0.664345
H	0.020717	3.542080	-0.170156
H	7.185898	-2.782931	-1.468791
H	5.437844	-2.719427	-1.721081
H	6.455927	-1.370329	-2.252782
H	8.302888	-1.299592	0.283159
H	7.354375	-0.127436	1.214729
H	7.649242	0.130655	-0.514170
H	-3.138371	2.878582	-0.217265
H	-2.515405	-2.587369	0.530006
H	-5.335349	0.507504	0.838484
H	-5.151775	0.439274	-0.879243
H	-6.444803	-1.832217	0.717285
H	-8.654871	-0.861248	0.041438
H	-7.755291	0.264708	1.070558
H	-7.832778	0.530201	-0.678852
H	-7.432396	-2.496975	-1.471715
H	-5.665787	-2.495160	-1.559102
H	-6.595440	-1.158868	-2.272109
H	1.737281	0.330802	2.551759
H	3.500988	0.383852	2.363357
H	2.559142	1.883568	2.350864
H	3.510741	1.193554	-2.129515

Conformer 73:

C	-2.566113	0.877044	-0.300126
C	-2.660697	-0.532604	-0.456995
C	-1.427406	-1.270781	-0.383914
C	-0.174603	-0.665124	-0.155735
C	-0.134108	0.714205	0.013995
C	-1.314684	1.514127	-0.067616
C	1.086815	-1.492228	-0.132989
C	2.310836	-0.597244	-0.354181
O	1.006342	1.390169	0.254910
C	3.642110	-1.327766	-0.127953
C	4.852127	-0.412889	-0.113009
C	4.708176	0.917056	0.031542
C	3.381446	1.633500	0.105747
C	6.206188	-1.100058	-0.247646
H	2.281351	-0.251517	-1.397350
C	-1.258718	2.945977	0.078398
C	7.387084	-0.151241	-0.493382
C	6.496350	-2.006674	0.970491
C	-3.903601	-1.273065	-0.711580
O	-3.854033	-2.517556	-0.871876
O	-3.658408	1.636978	-0.364725
O	-1.423678	-2.590932	-0.536476
O	-2.265453	3.684217	0.018704
C	-5.269377	-0.627632	-0.775863
C	-5.892464	-0.341729	0.619790

C	-7.193343	0.455179	0.442864
C	-6.138605	-1.630796	1.417621
C	2.242270	0.666380	0.515538
C	2.289753	0.396335	2.025500
O	3.112976	2.310460	-1.129709
H	1.175513	-2.038349	0.816917
H	1.034872	-2.261020	-0.911032
H	3.594094	-1.882495	0.820216
H	3.771663	-2.093354	-0.904834
H	5.577849	1.567677	0.032817
H	3.425820	2.437013	0.850103
H	6.127305	-1.760165	-1.125888
H	-0.272782	3.398171	0.244953
H	8.298552	-0.730762	-0.672193
H	7.220556	0.489331	-1.365374
H	7.574693	0.493705	0.372711
H	7.446498	-2.533773	0.833674
H	5.719591	-2.762263	1.120795
H	6.571180	-1.408389	1.886019
H	-3.356694	2.595658	-0.233459
H	-2.393862	-2.851882	-0.707765
H	-5.217852	0.307088	-1.336799
H	-5.909760	-1.334770	-1.313787
H	-5.189576	0.287257	1.178884
H	-7.646302	0.681306	1.414679
H	-7.011676	1.403600	-0.073808
H	-7.926720	-0.114778	-0.141568
H	-6.561423	-1.396405	2.401079
H	-5.218453	-2.201922	1.575605
H	-6.846103	-2.286581	0.895622
H	1.478247	-0.267165	2.335810
H	3.240048	-0.055174	2.321036
H	2.177939	1.340540	2.566437
H	3.476854	1.782890	-1.853105

Conformer 79:

C	2.572260	0.860401	-0.338484
C	2.620021	-0.553639	-0.475607
C	1.354272	-1.236656	-0.526174
C	0.112644	-0.573597	-0.442561
C	0.115176	0.812013	-0.328915
C	1.332703	1.556117	-0.264324
C	-1.178171	-1.354144	-0.449023
C	-2.315066	-0.500426	0.122294
O	-1.014059	1.544389	-0.263881
C	-3.697805	-1.148816	-0.042598
C	-4.849564	-0.222991	0.292678
C	-4.666139	1.106684	0.385937
C	-3.330559	1.799542	0.274587
C	-6.228849	-0.835310	0.495458
H	-2.122666	-0.373592	1.197215
C	1.325955	2.989342	-0.121327
C	-6.687374	-1.662461	-0.721963
C	-6.303830	-1.675415	1.787462
C	3.848710	-1.351292	-0.584491
O	3.761033	-2.596115	-0.725057
O	3.698367	1.569511	-0.276580
O	1.307748	-2.558461	-0.655415
O	2.365500	3.680913	-0.066148
C	5.241033	-0.765534	-0.519714
C	5.737692	-0.491768	0.928324
C	5.854229	-1.781966	1.753191
C	7.080410	0.251770	0.873333
C	-2.307765	0.912444	-0.480095
C	-2.576808	0.955474	-1.990348
O	-2.863737	2.198457	1.570677
H	-1.422870	-1.691469	-1.466227
H	-1.057124	-2.267453	0.142919
H	-3.815232	-1.502565	-1.077504
H	-3.745324	-2.052093	0.577963
H	-5.511487	1.751588	0.620596

H	-3.433864	2.740246	-0.278512
H	-6.930909	0.000681	0.609924
H	0.349257	3.485117	-0.053582
H	-7.717777	-2.005462	-0.579797
H	-6.653843	-1.070815	-1.643117
H	-6.063508	-2.551793	-0.865773
H	-7.327247	-2.031697	1.947871
H	-6.010703	-1.086042	2.662498
H	-5.655448	-2.557417	1.738539
H	3.427818	2.541979	-0.185629
H	2.278263	-2.864833	-0.710780
H	5.899273	-1.505377	-0.987633
H	5.285585	0.164457	-1.088951
H	5.010052	0.169626	1.413383
H	6.188681	-1.554760	2.771722
H	4.901319	-2.315496	1.823926
H	6.582661	-2.469421	1.306069
H	7.446557	0.468765	1.883070
H	6.987304	1.201905	0.336628
H	7.843155	-0.351925	0.365340
H	-1.845605	0.357514	-2.540649
H	-3.577460	0.586468	-2.228812
H	-2.500997	1.988074	-2.343407
H	-3.165728	1.547604	2.218097

Conformer 80:

C	-2.549905	0.847386	-0.341727
C	-2.598943	-0.570560	-0.429385
C	-1.345973	-1.265713	-0.294747
C	-0.116995	-0.610897	-0.073024
C	-0.121437	0.775686	0.028455
C	-1.323426	1.533647	-0.116301
C	1.168137	-1.396076	0.014214
C	2.368521	-0.475243	-0.228945
O	0.992977	1.497561	0.258007
C	3.716979	-1.155968	0.050430
C	4.893669	-0.201277	0.053586
C	4.707821	1.128472	0.140625
C	3.361373	1.809132	0.142463
C	6.296589	-0.788975	-0.016358
H	2.347289	-0.179570	-1.287501
C	-1.313607	2.972005	-0.040869
C	6.565735	-1.798345	1.117404
C	6.590737	-1.421125	-1.392759
C	-3.813357	-1.360851	-0.671288
O	-3.723292	-2.609797	-0.766389
O	-3.662812	1.568997	-0.466453
O	-1.299497	-2.591051	-0.380403
O	-2.340416	3.674854	-0.157806
C	-5.195898	-0.761951	-0.797158
C	-5.858800	-0.425998	0.568731
C	-7.177604	0.322052	0.324145
C	-6.085950	-1.680729	1.424778
C	2.244763	0.826122	0.577243
C	2.269323	0.630254	2.099059
O	3.099964	2.406808	-1.135001
H	1.254399	-1.892085	0.991433
H	1.154915	-2.203269	-0.725655
H	3.673614	-1.669965	1.021996
H	3.876281	-1.950391	-0.689018
H	5.570302	1.793055	0.149385
H	3.361125	2.651636	0.843813
H	6.995367	0.047523	0.113548
H	-0.345515	3.462189	0.122911
H	7.610526	-2.126002	1.092056
H	6.375864	-1.355244	2.100917
H	5.939984	-2.692661	1.020432
H	7.632133	-1.757839	-1.439233
H	6.432273	-0.701743	-2.202915
H	5.956952	-2.294253	-1.584215
H	-3.392909	2.541878	-0.377416

H	-2.257882	-2.890116	-0.556793
H	-5.159487	0.144713	-1.403472
H	-5.802552	-1.514514	-1.312062
H	-5.187232	0.250614	1.110447
H	-7.659058	0.582505	1.273408
H	-7.011428	1.248702	-0.235471
H	-7.880734	-0.297764	-0.246541
H	-6.537288	-1.410636	2.386155
H	-5.153634	-2.215794	1.630436
H	-6.762533	-2.382206	0.921522
H	1.468504	-0.038336	2.425671
H	3.224423	0.217664	2.433487
H	2.123541	1.595846	2.592059
H	3.526850	1.870799	-1.816448

Conformer 83:

C	2.583112	0.845183	-0.393461
C	2.675383	-0.572899	-0.427419
C	1.432072	-1.297585	-0.410899
C	0.170413	-0.669932	-0.359893
C	0.129130	0.719698	-0.347509
C	1.322429	1.504758	-0.353428
C	-1.094282	-1.489603	-0.293970
C	-2.253317	-0.632962	0.226466
O	-1.022413	1.418888	-0.320722
C	-3.616637	-1.330643	0.118184
C	-4.797363	-0.423660	0.409146
C	-4.652985	0.914060	0.415444
C	-3.340909	1.636561	0.227119
C	-6.126737	-1.124819	0.664117
H	-2.057329	-0.424456	1.287954
C	1.270485	2.943666	-0.314836
C	-7.237776	-0.217600	1.209740
C	-6.618317	-1.867408	-0.599539
C	3.928165	-1.337017	-0.494338
O	3.879664	-2.590985	-0.541542
O	3.686111	1.592296	-0.398688
O	1.427365	-2.626135	-0.442307
O	2.287456	3.670078	-0.324301
C	5.301168	-0.703968	-0.492637
C	5.805335	-0.307665	0.924112
C	5.974472	-1.528858	1.839950
C	7.121773	0.472842	0.795705
C	-2.296827	0.732319	-0.475148
C	-2.582563	0.658807	-1.981192
O	-2.872054	2.149607	1.481571
H	-1.337173	-1.908086	-1.281013
H	-0.938035	-2.352807	0.361466
H	-3.725762	-1.757939	-0.889064
H	-3.638607	-2.190563	0.801210
H	-5.500205	1.557856	0.633127
H	-3.485356	2.529034	-0.392786
H	-5.925161	-1.892450	1.427934
H	0.278956	3.411800	-0.269756
H	-8.121183	-0.816865	1.452776
H	-6.926681	0.305432	2.119737
H	-7.545059	0.533367	0.472918
H	-7.545168	-2.409749	-0.384730
H	-5.887915	-2.595651	-0.964264
H	-6.823204	-1.156297	-1.408290
H	3.384956	2.559735	-0.375199
H	2.406707	-2.904773	-0.487597
H	5.977862	-1.455163	-0.913806
H	5.308465	0.182098	-1.129632
H	5.061689	0.363880	1.369302
H	6.312240	-1.216245	2.834477
H	5.041028	-2.086838	1.962550
H	6.720760	-2.223100	1.434722
H	7.492462	0.775137	1.781561
H	6.990647	1.377444	0.192416
H	7.898107	-0.141534	0.322282

H	-1.841097	0.042393	-2.496279
H	-3.575214	0.247282	-2.179983
H	-2.538722	1.665016	-2.408106
H	-3.128377	1.532403	2.179705

Conformer 86:

C	-2.452032	1.123824	0.129931
C	-2.631321	-0.284724	0.164231
C	-1.437342	-1.083284	0.223819
C	-0.138799	-0.534183	0.229530
C	-0.011805	0.850496	0.203545
C	-1.153371	1.706333	0.145286
C	1.075218	-1.429741	0.236375
C	2.308617	-0.652679	-0.235795
O	1.180824	1.477820	0.225590
C	3.619593	-1.430232	-0.051851
C	4.866434	-0.601113	-0.295568
C	4.804920	0.742671	-0.323880
C	3.532221	1.546398	-0.208536
C	6.160767	-1.385375	-0.478434
H	2.177850	-0.448298	-1.308080
C	-1.013213	3.139478	0.104029
C	7.351092	-0.554367	-0.976081
C	6.543216	-2.142324	0.814123
C	-3.933290	-0.968141	0.139177
O	-3.968602	-2.218828	0.210387
O	-3.509767	1.933097	0.090888
O	-1.514631	-2.410635	0.270346
O	-1.983797	3.925284	0.062867
C	-5.245504	-0.214106	0.043216
C	-6.496218	-1.096649	-0.109450
C	-7.755404	-0.269335	0.195568
C	-6.584599	-1.726317	-1.509033
C	2.401347	0.717597	0.451283
C	2.608133	0.648485	1.970424
O	3.157825	2.069693	-1.490095
H	1.243627	-1.847332	1.239215
H	0.899023	-2.291595	-0.415779
H	3.653358	-1.847701	0.964835
H	3.621729	-2.300360	-0.722197
H	5.699476	1.330661	-0.507280
H	3.701146	2.437053	0.407764
H	5.949540	-2.147941	-1.244747
H	0.005904	3.546840	0.104321
H	8.206863	-1.209314	-1.169196
H	7.118124	-0.024414	-1.905217
H	7.667195	0.185380	-0.231684
H	7.444444	-2.742487	0.650021
H	5.752491	-2.820617	1.148102
H	6.751476	-1.436029	1.626230
H	-3.152864	2.880518	0.071573
H	-2.506055	-2.630310	0.262850
H	-5.319681	0.410653	0.943660
H	-5.180256	0.510513	-0.776660
H	-6.430610	-1.909452	0.623752
H	-8.657744	-0.883744	0.101972
H	-7.732522	0.139555	1.212374
H	-7.854761	0.573350	-0.500480
H	-7.473700	-2.361442	-1.593486
H	-5.709949	-2.345467	-1.725729
H	-6.658690	-0.949160	-2.281155
H	1.805507	0.086349	2.455085
H	3.562404	0.179652	2.223109
H	2.605981	1.661577	2.383070
H	3.403931	1.425853	-2.167568

Conformer 87:

C	-2.552405	0.845594	-0.373878
C	-2.651660	-0.572213	-0.398616
C	-1.426001	-1.304684	-0.218239
C	-0.176297	-0.684698	-0.012706

C	-0.131350	0.704510	0.024726
C	-1.304104	1.497289	-0.166267
C	1.078821	-1.510598	0.123280
C	2.312031	-0.644830	-0.154007
O	1.006453	1.396218	0.231581
C	3.635814	-1.353529	0.163603
C	4.847508	-0.443511	0.144332
C	4.710760	0.894164	0.174305
C	3.385717	1.615868	0.121870
C	6.197101	-1.151267	0.112750
H	2.305213	-0.397202	-1.225074
C	-1.243214	2.936327	-0.155281
C	6.529507	-1.631672	-1.318396
C	7.363247	-0.331243	0.683593
C	-3.891140	-1.328908	-0.619902
O	-3.845247	-2.583308	-0.657606
O	-3.637479	1.599886	-0.542630
O	-1.426722	-2.633356	-0.243189
O	-2.242921	3.669312	-0.314396
C	-5.249639	-0.687567	-0.790459
C	-5.915920	-0.267435	0.550200
C	-7.204253	0.514740	0.255047
C	-6.197837	-1.473501	1.458320
C	2.231007	0.695440	0.591256
C	2.237866	0.568634	2.120666
O	3.160583	2.156079	-1.187718
H	1.141038	-1.961553	1.123841
H	1.041512	-2.351441	-0.577206
H	3.576260	-1.826475	1.156390
H	3.778821	-2.183469	-0.540018
H	5.586943	1.536765	0.176860
H	3.404554	2.493341	0.778509
H	6.089543	-2.050640	0.738307
H	-0.259637	3.398652	-0.003170
H	7.443647	-2.235170	-1.317651
H	5.729111	-2.243894	-1.746045
H	6.690250	-0.774168	-1.981566
H	8.268460	-0.945982	0.725182
H	7.148156	0.025547	1.695937
H	7.591413	0.538908	0.057993
H	-3.333675	2.565644	-0.494272
H	-2.393462	-2.905699	-0.417162
H	-5.173537	0.189164	-1.435852
H	-5.876992	-1.440434	-1.279454
H	-5.226957	0.408873	1.070001
H	-7.686967	0.834689	1.185295
H	-6.998654	1.408661	-0.343375
H	-7.922468	-0.105192	-0.296409
H	-6.651282	-1.144531	2.400156
H	-5.287625	-2.030765	1.700787
H	-6.892500	-2.173115	0.977611
H	1.407533	-0.050213	2.470836
H	3.172611	0.132347	2.481489
H	2.129608	1.561017	2.568090
H	3.568401	1.566217	-1.835535

Table S20. Eucalypcamal K (**2b**) DFT geometry optimized conformers calculated at the B3LYP/6-31+G(d,p) level of theory.

Conformer 5:

C	1.816942	-1.205267	-0.466279
C	1.935500	-0.588789	0.806386
C	0.720391	-0.415006	1.559183
C	-0.559027	-0.816059	1.089155
C	-0.619297	-1.380955	-0.178749
C	0.545634	-1.595453	-0.971977
C	-1.748938	-0.728074	2.027416
C	-3.052644	-0.069363	1.516726

O	-1.786343	-1.817716	-0.730198
C	-2.820191	1.377248	1.024149
C	-2.429149	1.510219	-0.437028
C	-2.518754	0.461848	-1.275951
C	-2.945829	-0.914088	-0.842561
C	-1.965013	2.893637	-0.877677
H	-3.722599	-0.023004	2.383949
C	0.455612	-2.187153	-2.284156
C	-1.296186	2.936332	-2.258655
C	-3.118580	3.921218	-0.813499
C	3.206399	-0.152702	1.409773
O	3.199215	0.349872	2.559348
O	2.894464	-1.416060	-1.221114
O	0.772383	0.132296	2.768904
O	1.445878	-2.399876	-3.015011
C	4.546027	-0.252344	0.716643
C	4.808074	0.890086	-0.305659
C	4.854593	2.269135	0.368811
C	6.111225	0.602803	-1.065845
C	-3.792599	-0.896297	0.443244
C	-4.170867	-2.303322	0.901703
O	-5.053929	-0.276090	0.122408
H	-1.982964	-1.738903	2.383649
H	-1.427984	-0.172684	2.909745
H	-3.728506	1.968271	1.200795
H	-2.047176	1.853175	1.641949
H	-2.233163	0.563888	-2.318676
H	-3.531076	-1.398371	-1.629446
H	-1.213298	3.208858	-0.137913
H	-0.544297	-2.460838	-2.643808
H	-0.902039	3.939767	-2.449116
H	-0.463437	2.229631	-2.329276
H	-2.007514	2.708444	-3.060831
H	-2.752126	4.918337	-1.079137
H	-3.561531	3.988885	0.184305
H	-3.911544	3.655213	-1.522468
H	2.572490	-1.852151	-2.076122
H	1.756612	0.334650	2.945455
H	5.297757	-0.197898	1.511136
H	4.635307	-1.211599	0.204365
H	3.988743	0.883750	-1.034288
H	5.023366	3.053549	-0.377537
H	3.925386	2.503401	0.897622
H	5.670042	2.321183	1.100404
H	6.312585	1.388115	-1.802969
H	6.059469	-0.353562	-1.596925
H	6.966150	0.563974	-0.379075
H	-4.752266	-2.247686	1.827298
H	-4.795354	-2.774925	0.137230
H	-3.295831	-2.933040	1.066491
H	-4.888486	0.556240	-0.342418

Conformer 6:

C	1.591111	-1.402340	-0.584041
C	1.897321	-0.353664	0.320592
C	0.817612	0.134360	1.136745
C	-0.506716	-0.376001	1.074537
C	-0.756474	-1.379268	0.146134
C	0.267395	-1.913955	-0.688234
C	-1.528986	0.102706	2.089267
C	-2.918750	0.559739	1.585103
O	-1.982440	-1.958321	0.006880
C	-2.814380	1.699475	0.544620
C	-2.684535	1.244288	-0.896128
C	-2.883772	-0.041233	-1.241502
C	-3.176526	-1.133424	-0.250599
C	-2.356319	2.288624	-1.954484
H	-3.438542	0.958904	2.464609
C	-0.017397	-2.955668	-1.644395
C	-3.369958	3.450230	-1.971450
C	-0.915848	2.822540	-1.809201

C	3.236175	0.249323	0.480795
O	3.392875	1.168823	1.315831
O	2.538584	-1.918867	-1.366117
O	1.045580	1.105214	2.017157
O	0.845259	-3.459932	-2.393367
C	4.426925	-0.210895	-0.336099
C	5.778789	0.494485	-0.095143
C	5.767280	1.983696	-0.480128
C	6.342746	0.269178	1.318100
C	-3.798480	-0.591558	1.050675
C	-4.048839	-1.699945	2.071561
O	-5.115183	-0.100069	0.729859
H	-1.669759	-0.687388	2.837097
H	-1.086097	0.941544	2.627859
H	-3.699043	2.345653	0.623457
H	-1.967172	2.346502	0.802356
H	-2.796733	-0.339306	-2.284906
H	-3.866305	-1.865907	-0.679281
H	-2.419571	1.781661	-2.926130
H	-1.053068	-3.313750	-1.702509
H	-3.158758	4.121324	-2.810746
H	-4.397156	3.086192	-2.084207
H	-3.318355	4.047064	-1.054280
H	-0.683373	3.503393	-2.635229
H	-0.185874	2.007397	-1.825754
H	-0.780413	3.380112	-0.876192
H	2.095658	-2.632491	-1.930346
H	2.030851	1.340015	1.929044
H	4.536049	-1.290618	-0.175327
H	4.149279	-0.132816	-1.394375
H	6.464679	-0.007554	-0.793298
H	6.783150	2.393382	-0.444249
H	5.390104	2.126383	-1.500169
H	5.142061	2.567182	0.200365
H	7.368525	0.649612	1.382901
H	6.370573	-0.798401	1.567908
H	5.742339	0.781272	2.074089
H	-4.466802	-1.272598	2.988373
H	-4.778338	-2.405777	1.663623
H	-3.139034	-2.249526	2.315697
H	-5.056503	0.479611	-0.042664

Conformer 9:

C	1.514057	1.382154	0.560526
C	1.866911	0.354121	-0.350632
C	0.814845	-0.160884	-1.185914
C	-0.526229	0.305374	-1.137728
C	-0.821428	1.290114	-0.202808
C	0.172917	1.848830	0.651463
C	-1.518275	-0.197008	-2.171027
C	-2.899777	-0.701366	-1.689742
O	-2.066741	1.828720	-0.075335
C	-2.773088	-1.840166	-0.653149
C	-2.682990	-1.396698	0.794614
C	-2.931672	-0.122829	1.149062
C	-3.238842	0.965769	0.156689
C	-2.333336	-2.492663	1.793954
H	-3.394727	-1.111272	-2.578618
C	-0.159027	2.870189	1.614286
C	-0.814347	-2.780745	1.781823
C	-2.813007	-2.227733	3.228783
C	3.226778	-0.203027	-0.498110
O	3.425162	-1.107773	-1.340473
O	2.433181	1.920754	1.361337
O	1.086360	-1.115960	-2.071438
O	0.676129	3.393892	2.380961
C	4.390270	0.286620	0.340709
C	5.768007	-0.370678	0.110796
C	5.800771	-1.864030	0.478106
C	6.343440	-0.109563	-1.291606
C	-3.822969	0.417101	-1.158859

C	-4.091783	1.526774	-2.173656
O	-5.128664	-0.117933	-0.863554
H	-1.672950	0.594064	-2.915072
H	-1.041660	-1.017388	-2.709278
H	-3.634966	-2.517899	-0.740311
H	-1.902597	-2.460864	-0.899359
H	-2.886669	0.178071	2.191943
H	-3.957216	1.674516	0.578191
H	-2.836953	-3.405150	1.440119
H	-1.206523	3.193833	1.661261
H	-0.586690	-3.641281	2.420061
H	-0.442614	-3.003631	0.777355
H	-0.258090	-1.917919	2.163986
H	-2.619793	-3.104940	3.854902
H	-3.886548	-2.015602	3.265044
H	-2.283834	-1.382996	3.683614
H	1.959267	2.613281	1.926655
H	2.077969	-1.318139	-1.972787
H	4.465631	1.371215	0.194148
H	4.100778	0.186512	1.393982
H	6.427157	0.145266	0.824405
H	6.830111	-2.239394	0.451886
H	5.414484	-2.031542	1.490945
H	5.204734	-2.459522	-0.217979
H	7.381791	-0.455872	-1.346752
H	6.339992	0.961415	-1.528038
H	5.770199	-0.631221	-2.062003
H	-4.482359	1.095151	-3.100494
H	-4.848824	2.206151	-1.771026
H	-3.195736	2.106198	-2.399000
H	-5.064147	-0.702572	-0.095241

Conformer 11:

C	1.669168	1.096407	0.633496
C	1.917399	0.110067	-0.354549
C	0.817330	-0.243244	-1.211238
C	-0.467049	0.357466	-1.126357
C	-0.660029	1.301665	-0.125184
C	0.383609	1.692140	0.763131
C	-1.504362	0.032982	-2.185737
C	-2.930769	-0.361099	-1.733468
O	-1.844929	1.953486	0.045416
C	-2.924248	-1.580116	-0.782940
C	-2.777190	-1.252315	0.692456
C	-2.885384	0.013951	1.134883
C	-3.096181	1.194960	0.227182
C	-2.531383	-2.440027	1.615740
H	-3.463443	-0.652721	-2.646866
C	0.155079	2.667206	1.801184
C	-2.088202	-2.066717	3.037198
C	-3.762145	-3.374360	1.670621
C	3.216606	-0.557460	-0.557790
O	3.316618	-1.446520	-1.433744
O	2.634515	1.467285	1.474008
O	0.988942	-1.165184	-2.154845
O	1.035954	3.039908	2.603886
C	4.443645	-0.218185	0.265271
C	5.741473	-0.913310	-0.181263
C	6.279711	-0.328761	-1.497208
C	6.795470	-0.808026	0.932756
C	-3.735927	0.803619	-1.117561
C	-3.894567	2.003682	-2.049068
O	-5.088058	0.382358	-0.847636
H	-1.576913	0.886729	-2.870741
H	-1.113313	-0.791449	-2.783552
H	-3.849870	-2.153184	-0.925624
H	-2.117804	-2.265810	-1.074841
H	-2.774746	0.240247	2.191181
H	-3.739944	1.936991	0.707986
H	-1.712854	-3.014605	1.155803
H	-0.852640	3.095675	1.874006

H	-1.833826	-2.972810	3.596482
H	-1.207121	-1.417459	3.031395
H	-2.885694	-1.556512	3.589591
H	-3.545343	-4.249585	2.291988
H	-4.053953	-3.737975	0.681150
H	-4.621227	-2.854617	2.111128
H	2.234354	2.160931	2.091840
H	1.950566	-1.482930	-2.067887
H	4.572342	0.870250	0.280029
H	4.207272	-0.470753	1.307846
H	5.519575	-1.974234	-0.347645
H	7.195852	-0.845262	-1.804931
H	5.552612	-0.427873	-2.307673
H	6.523618	0.735225	-1.379200
H	7.725159	-1.306889	0.637660
H	6.447002	-1.272130	1.862700
H	7.037264	0.239552	1.152963
H	-4.330140	1.680156	-2.999572
H	-4.577451	2.725795	-1.591853
H	-2.945227	2.504314	-2.242709
H	-5.080761	-0.262887	-0.126651

Conformer 12:

C	1.637103	-1.200921	-0.552419
C	1.867570	-0.124424	0.341391
C	0.750156	0.312710	1.134477
C	-0.534835	-0.290295	1.078890
C	-0.709226	-1.327085	0.170048
C	0.352086	-1.803110	-0.653254
C	-1.593090	0.137832	2.079277
C	-3.009269	0.485300	1.561289
O	-1.891386	-1.992390	0.037698
C	-2.980375	1.608809	0.498803
C	-2.809265	1.135644	-0.931758
C	-2.916402	-0.167187	-1.252289
C	-3.138421	-1.257584	-0.241234
C	-2.546320	2.179273	-2.008822
H	-3.560164	0.863956	2.430874
C	0.142213	-2.872347	-1.598425
C	-3.637349	3.267674	-2.055052
C	-1.147098	2.813996	-1.865591
C	3.165540	0.555283	0.509932
O	3.249842	1.525759	1.296462
O	2.619317	-1.653382	-1.331046
O	0.904799	1.321567	1.987799
O	1.039357	-3.322894	-2.340941
C	4.409749	0.131627	-0.245369
C	5.701035	0.862331	0.161444
C	6.203894	0.409872	1.541868
C	6.780592	0.641631	-0.910415
C	-3.803261	-0.735285	1.046641
C	-3.980569	-1.838109	2.088563
O	-5.149652	-0.343830	0.711275
H	-1.681220	-0.643590	2.844114
H	-1.212426	1.017334	2.600391
H	-3.909466	2.191387	0.560370
H	-2.183237	2.319734	0.747635
H	-2.801996	-0.478951	-2.289030
H	-3.773311	-2.044453	-0.658178
H	-2.567352	1.650318	-2.970528
H	-0.865837	-3.302539	-1.653711
H	-3.466347	3.935419	-2.906087
H	-4.636185	2.831630	-2.167123
H	-3.634039	3.884261	-1.149555
H	-0.956161	3.493239	-2.703507
H	-0.362609	2.051011	-1.861856
H	-1.057029	3.397395	-0.942953
H	2.229652	-2.402386	-1.888172
H	1.869290	1.627431	1.892022
H	4.531852	-0.953461	-0.149080
H	4.199927	0.280409	-1.313233

H	5.482390	1.935538	0.215630
H	7.116071	0.950382	1.818561
H	5.458543	0.592839	2.320521
H	6.443195	-0.661671	1.536660
H	7.706358	1.163364	-0.643934
H	6.457637	1.011919	-1.890305
H	7.020389	-0.423792	-1.018872
H	-4.432862	-1.423144	2.994708
H	-4.656115	-2.601699	1.691894
H	-3.035667	-2.317040	2.347639
H	-5.128247	0.222704	-0.072843

Conformer 13:

C	-1.812748	-0.758691	0.625057
C	-1.902189	0.335148	-0.274860
C	-0.749578	0.594292	-1.098019
C	0.437477	-0.185313	-1.055201
C	0.480546	-1.223386	-0.132888
C	-0.623848	-1.535145	0.712974
C	1.526742	0.075780	-2.079309
C	2.986685	0.226545	-1.589591
O	1.558390	-2.047887	-0.008952
C	3.135946	1.356133	-0.543613
C	2.928584	0.929692	0.896744
C	2.856943	-0.371112	1.234987
C	2.902637	-1.494271	0.236639
C	2.838487	2.013318	1.962555
H	3.569495	0.512843	-2.473575
C	-0.552756	-2.617063	1.663931
C	4.076148	2.932702	1.975145
C	1.543424	2.842280	1.833096
C	-3.075757	1.215240	-0.408655
O	-3.035344	2.172273	-1.219116
O	-2.837877	-1.072196	1.416203
O	-0.776385	1.602087	-1.963555
O	-1.493162	-2.931122	2.423547
C	-4.360730	1.030174	0.365203
C	-5.299159	-0.056451	-0.233617
C	-6.486874	-0.279160	0.714177
C	-5.779833	0.309646	-1.645530
C	3.609772	-1.087606	-1.070444
C	3.609007	-2.217634	-2.098293
O	5.004089	-0.886956	-0.764226
H	1.490133	-0.720359	-2.833056
H	1.264171	0.993286	-2.607737
H	4.135971	1.802021	-0.630316
H	2.441376	2.168672	-0.788826
H	2.719984	-0.650688	2.278154
H	3.427246	-2.358290	0.654108
H	2.800483	1.498455	2.931360
H	0.384520	-3.185920	1.708026
H	4.018847	3.628690	2.818769
H	5.003278	2.357988	2.077689
H	4.145644	3.532009	1.060768
H	1.467013	3.550456	2.665286
H	0.656906	2.200930	1.851103
H	1.522707	3.423292	0.904814
H	-2.543674	-1.861735	1.977814
H	-1.687586	2.047360	-1.850270
H	-4.143618	0.774990	1.403581
H	-4.871479	1.998353	0.331606
H	-4.735019	-0.995024	-0.287757
H	-7.071823	0.641507	0.833996
H	-7.158307	-1.050576	0.320968
H	-6.150108	-0.598452	1.706187
H	-6.425105	-0.480540	-2.045378
H	-4.948482	0.448310	-2.343645
H	-6.358018	1.241618	-1.634321
H	4.097076	-1.882434	-3.018771
H	4.178006	-3.063780	-1.701978
H	2.601052	-2.561743	-2.332885

H	5.078060	-0.311520	0.010147

Conformer 15:

C	1.638059	-1.373312	-0.421205
C	1.863773	-0.349895	0.533712
C	0.723052	0.099281	1.286054
C	-0.590894	-0.406457	1.098201
C	-0.760776	-1.380676	0.120625
C	0.330054	-1.885661	-0.645248
C	-1.685186	0.038886	2.052533
C	-3.058691	0.467079	1.482642
O	-1.966000	-1.962176	-0.135577
C	-2.961452	1.657325	0.503216
C	-2.664594	1.279024	-0.931630
C	-2.782744	0.009418	-1.350330
C	-3.145119	-1.130650	-0.448417
C	-2.282191	2.385654	-1.903681
H	-3.635695	0.804730	2.355786
C	0.128824	-2.898430	-1.652310
C	-3.354493	3.491257	-1.978022
C	-0.896877	2.986376	-1.587555
C	3.178163	0.259705	0.806345
O	3.278194	1.120770	1.710016
O	2.650651	-1.868296	-1.132221
O	0.881475	1.028564	2.225102
O	1.052725	-3.380752	-2.340394
C	4.427848	-0.124508	0.038748
C	5.674662	0.718388	0.357590
C	6.930795	0.004398	-0.167147
C	5.571031	2.136951	-0.225561
C	-3.857341	-0.686844	0.841728
C	-4.109560	-1.867457	1.784443
O	-5.128691	-0.187963	0.373677
H	-1.846352	-0.761122	2.786698
H	-1.294871	0.882513	2.623454
H	-3.913797	2.201593	0.511335
H	-2.206640	2.363706	0.869696
H	-2.587499	-0.241375	-2.391357
H	-3.792903	-1.842223	-0.967561
H	-2.217866	1.922596	-2.897049
H	-0.897645	-3.253256	-1.808851
H	-3.100582	4.210618	-2.764223
H	-4.341503	3.074555	-2.204368
H	-3.427521	4.047099	-1.036523
H	-0.620745	3.719580	-2.353437
H	-0.123505	2.212192	-1.564529
H	-0.887669	3.502542	-0.621268
H	2.259274	-2.566329	-1.751217
H	1.870860	1.260970	2.228837
H	4.614491	-1.186623	0.247672
H	4.208295	-0.101292	-1.034879
H	5.756871	0.805562	1.447580
H	7.832092	0.584507	0.059742
H	7.048606	-0.987603	0.284041
H	6.887035	-0.127405	-1.255801
H	6.462065	2.724511	0.022801
H	4.700315	2.669320	0.166640
H	5.492061	2.102731	-1.320188
H	-4.598691	-1.520468	2.703703
H	-4.769824	-2.590072	1.294342
H	-3.191075	-2.385553	2.063683
H	-5.722472	-0.106297	1.132034

Conformer 16:

C	-1.620438	-1.375132	0.421271
C	-1.851181	-0.359304	-0.540748
C	-0.714584	0.084567	-1.302375
C	0.599488	-0.422508	-1.118844
C	0.774699	-1.386203	-0.132686
C	-0.311102	-1.885436	0.643650
C	1.695363	0.010284	-2.075801

C	3.048608	0.482140	-1.492160
O	1.983466	-1.962408	0.120572
C	2.878427	1.665964	-0.511409
C	2.640732	1.273818	0.934089
C	2.805057	0.003521	1.347349
C	3.161382	-1.131412	0.427930
C	2.243473	2.364028	1.919955
H	3.633341	0.840891	-2.347986
C	-0.104902	-2.892216	1.655896
C	3.262571	3.520177	1.963259
C	0.822225	2.898496	1.645188
C	-3.167404	0.248111	-0.811871
O	-3.270281	1.105033	-1.718920
O	-2.629366	-1.863884	1.141620
O	-0.876022	1.010101	-2.244226
O	-1.024808	-3.367568	2.353695
C	-4.414621	-0.134404	-0.039708
C	-5.664463	0.702696	-0.362105
C	-6.917646	-0.011908	0.168791
C	-5.564483	2.125202	0.212046
C	3.878949	-0.649044	-0.847254
C	4.193420	-1.801458	-1.799301
O	5.173053	-0.150216	-0.453753
H	1.883643	-0.809855	-2.779555
H	1.297988	0.828304	-2.678225
H	3.771596	2.303861	-0.553189
H	2.057361	2.305477	-0.857447
H	2.640034	-0.249299	2.393284
H	3.813491	-1.847152	0.936402
H	2.230263	1.898761	2.914167
H	0.921819	-3.249135	1.806701
H	2.994848	4.227958	2.754947
H	4.275427	3.156137	2.168109
H	3.284529	4.077125	1.020167
H	0.533935	3.613940	2.422971
H	0.086629	2.088280	1.639945
H	0.761568	3.417869	0.682722
H	-2.235594	-2.557122	1.764078
H	-1.865571	1.242582	-2.245471
H	-4.598429	-1.198466	-0.241174
H	-4.193386	-0.103412	1.033397
H	-5.748414	0.782760	-1.452493
H	-7.820980	0.563936	-0.060685
H	-7.032939	-1.007075	-0.275973
H	-6.872156	-0.136624	1.258207
H	-6.457721	2.708314	-0.038722
H	-4.696093	2.657965	-0.184780
H	-5.484013	2.098046	1.306777
H	4.680627	-1.415805	-2.700278
H	4.885350	-2.493107	-1.309754
H	3.299260	-2.356175	-2.085993
H	5.063819	0.462672	0.286973

Conformer 17:

C	-1.565135	-1.227101	0.403655
C	-1.842047	-0.079007	-0.381079
C	-0.749654	0.468860	-1.139308
C	0.554108	-0.094868	-1.156827
C	0.773715	-1.207756	-0.353840
C	-0.260514	-1.793757	0.432439
C	1.583964	0.462375	-2.122656
C	2.996314	0.800207	-1.588591
O	1.976422	-1.846404	-0.300792
C	2.947821	1.805034	-0.415866
C	2.821755	1.190215	0.964990
C	2.977017	-0.132739	1.154574
C	3.207478	-1.106759	0.031035
C	2.548283	2.173672	2.096386
H	3.523275	1.282418	-2.420994
C	-0.003814	-2.940246	1.269230
C	1.053003	2.565343	2.131935

C	3.006839	1.698241	3.483014
C	-3.163359	0.569506	-0.471659
O	-3.288756	1.608106	-1.159676
O	-2.521449	-1.782948	1.146806
O	-0.947308	1.550512	-1.888431
O	-0.875549	-3.487833	1.975987
C	-4.384339	0.032840	0.248938
C	-5.704436	0.751304	-0.079783
C	-6.203720	0.411869	-1.493526
C	-6.765495	0.392720	0.973108
C	3.833986	-0.440179	-1.208469
C	4.024708	-1.429472	-2.356624
O	5.174136	-0.042719	-0.855791
H	1.683554	-0.236318	-2.962420
H	1.169975	1.377112	-2.549104
H	3.855849	2.425668	-0.421902
H	2.124230	2.511694	-0.576942
H	2.907646	-0.558699	2.151544
H	3.871108	-1.913238	0.355365
H	3.114594	3.086273	1.855829
H	1.017899	-3.340724	1.271930
H	0.885002	3.352706	2.874599
H	0.698835	2.937634	1.166117
H	0.437716	1.701902	2.407311
H	2.874973	2.501409	4.215398
H	4.062847	1.409021	3.483959
H	2.419375	0.842116	3.832864
H	-2.100507	-2.568119	1.625799
H	-1.920226	1.813073	-1.754583
H	-4.469113	-1.042314	0.053105
H	-4.169395	0.089666	1.324644
H	-5.524386	1.832045	-0.034533
H	-7.136969	0.942552	-1.713011
H	-5.472558	0.693557	-2.256031
H	-6.404813	-0.663423	-1.587344
H	-7.711534	0.903381	0.762173
H	-6.446692	0.681341	1.981404
H	-6.966520	-0.686111	0.982805
H	4.448849	-0.913799	-3.223840
H	4.728893	-2.207446	-2.047324
H	3.089609	-1.909163	-2.648068
H	5.149651	0.444271	-0.020024

Conformer 21:

C	-1.740765	-0.791609	0.555173
C	-1.882219	0.340098	-0.289255
C	-0.751305	0.681029	-1.112474
C	0.462979	-0.056527	-1.123750
C	0.556815	-1.134920	-0.252868
C	-0.523331	-1.526825	0.591113
C	1.526822	0.292771	-2.148521
C	2.987564	0.471283	-1.670582
O	1.664547	-1.925084	-0.182767
C	3.110044	1.547338	-0.568539
C	2.945146	1.045969	0.853511
C	2.928186	-0.270713	1.129688
C	2.992826	-1.337623	0.070800
C	2.840058	2.128441	1.920833
H	3.547835	0.822750	-2.545595
C	-0.399222	-2.649353	1.488094
C	1.412594	2.721079	1.960631
C	3.271719	1.685513	3.326709
C	-3.088496	1.181955	-0.365592
O	-3.092761	2.179673	-1.126453
O	-2.743060	-1.180024	1.342488
O	-0.825864	1.729497	-1.925680
O	-1.316353	-3.034092	2.243625
C	-4.355974	0.911046	0.411910
C	-5.261713	-0.178925	-0.229601
C	-6.429207	-0.490609	0.718264
C	-5.772251	0.237372	-1.616921

C	3.664037	-0.843572	-1.224625
C	3.686553	-1.922035	-2.306225
O	5.055257	-0.608192	-0.928997
H	1.506586	-0.466370	-2.940127
H	1.224542	1.225132	-2.627412
H	4.092447	2.037261	-0.636573
H	2.383999	2.347365	-0.759522
H	2.832869	-0.617878	2.154666
H	3.552841	-2.203440	0.435117
H	3.516313	2.938167	1.607045
H	0.558945	-3.184113	1.493252
H	1.373535	3.569538	2.652199
H	1.083559	3.075964	0.979521
H	0.695192	1.968555	2.305584
H	3.270428	2.544657	4.005466
H	4.278997	1.256320	3.325513
H	2.585403	0.941380	3.745889
H	-2.413199	-1.984059	1.861635
H	-1.750695	2.135232	-1.779075
H	-4.116237	0.613459	1.433920
H	-4.902204	1.860051	0.432203
H	-4.664861	-1.092533	-0.335398
H	-7.076946	-1.266272	0.294828
H	-6.069343	-0.844833	1.690108
H	-7.045565	0.401137	0.888650
H	-6.393303	-0.555592	-2.048372
H	-4.954855	0.440185	-2.315820
H	-6.383566	1.145859	-1.554156
H	4.147235	-1.525150	-3.216239
H	4.291688	-2.765164	-1.960217
H	2.687771	-2.290694	-2.542673
H	5.121307	-0.068231	-0.128778

Conformer 23:

C	1.543848	1.348605	0.428825
C	1.817172	0.377366	-0.567384
C	0.703358	-0.073717	-1.357733
C	-0.627868	0.385551	-1.172282
C	-0.844580	1.304803	-0.152545
C	0.216490	1.808242	0.654833
C	-1.698894	-0.044928	-2.157994
C	-3.042138	-0.580134	-1.607044
O	-2.073339	1.833304	0.107604
C	-2.842697	-1.786904	-0.662904
C	-2.640319	-1.443482	0.800630
C	-2.853943	-0.196720	1.258957
C	-3.229518	0.957144	0.369323
C	-2.220642	-2.604932	1.693227
H	-3.606313	-0.928387	-2.480836
C	-0.032534	2.769257	1.701273
C	-0.706884	-2.885138	1.550228
C	-2.594188	-2.442039	3.174108
C	3.154622	-0.178527	-0.844566
O	3.294065	-0.998706	-1.780329
O	2.528919	1.841654	1.178717
O	0.903885	-0.959969	-2.329594
O	0.864180	3.246701	2.427346
C	4.381023	0.214657	-0.044928
C	5.659988	-0.570807	-0.382700
C	6.884541	0.163349	0.186915
C	5.599368	-2.015227	0.139928
C	-3.915536	0.499857	-0.931935
C	-4.255440	1.676435	-1.844940
O	-5.197778	-0.053692	-0.574232
H	-1.905371	0.794613	-2.833189
H	-1.269534	-0.827312	-2.785309
H	-3.711955	-2.457451	-0.730048
H	-1.996397	-2.389063	-1.016373
H	-2.728180	0.032182	2.313465
H	-3.909326	1.636115	0.891674
H	-2.749791	-3.492603	1.314489

H	-1.071653	3.087881	1.852936
H	-0.433829	-3.787384	2.107981
H	-0.409968	-3.035145	0.508016
H	-0.123513	-2.048786	1.950402
H	-2.356870	-3.359817	3.722037
H	-3.662008	-2.237152	3.302934
H	-2.032423	-1.628823	3.646933
H	2.106578	2.498146	1.821937
H	1.900476	-1.160741	-2.327763
H	4.533083	1.290540	-0.206050
H	4.149765	0.137936	1.023745
H	5.757349	-0.609429	-1.474224
H	7.807892	-0.375479	-0.052584
H	6.972746	1.176791	-0.221280
H	6.824218	0.247838	1.279470
H	6.512993	-2.560910	-0.121412
H	4.752148	-2.560387	-0.284848
H	5.507309	-2.029459	1.233989
H	-4.717344	1.309256	-2.766733
H	-4.976591	2.326006	-1.340181
H	-3.376536	2.270652	-2.097587
H	-5.077794	-0.690240	0.144562

Conformer 30:

C	-1.831268	-0.661664	0.663040
C	-1.943797	0.354301	-0.321349
C	-0.811817	0.544101	-1.190761
C	0.376784	-0.230253	-1.112480
C	0.444365	-1.186908	-0.107417
C	-0.638906	-1.427869	0.787261
C	1.440462	-0.058808	-2.181288
C	2.911003	0.137573	-1.741393
O	1.528982	-1.994814	0.060510
C	3.079096	1.351550	-0.799315
C	2.897834	1.056003	0.679107
C	2.834492	-0.210506	1.129581
C	2.874418	-1.414709	0.228812
C	2.821023	2.271724	1.595310
H	3.471977	0.347505	-2.660246
C	-0.542354	-2.426588	1.823183
C	2.344039	1.970323	3.022874
C	4.165762	3.033947	1.634062
C	-3.121458	1.221022	-0.498951
O	-3.101237	2.108902	-1.385224
O	-2.837317	-0.908968	1.500919
O	-0.860877	1.477104	-2.135738
O	-1.463652	-2.677463	2.628320
C	-4.387227	1.100780	0.318257
C	-5.339050	-0.029917	-0.167024
C	-6.503012	-0.173876	0.824500
C	-5.854448	0.220798	-1.591840
C	3.552165	-1.123479	-1.122689
C	3.536704	-2.339227	-2.047219
O	4.951418	-0.891652	-0.864202
H	1.389147	-0.918780	-2.860377
H	1.162070	0.807953	-2.782284
H	4.074010	1.790473	-0.951044
H	2.373089	2.140165	-1.091470
H	2.700530	-0.413243	2.187957
H	3.413045	-2.235600	0.710690
H	2.082520	2.947334	1.137202
H	0.397766	-2.988261	1.892645
H	2.218356	2.905966	3.577175
H	1.383689	1.445391	3.028691
H	3.070616	1.361241	3.572935
H	4.073030	3.932982	2.252296
H	4.494234	3.351105	0.640124
H	4.952048	2.406448	2.070193
H	-2.528813	-1.649054	2.118874
H	-1.769143	1.931086	-2.037524
H	-4.144512	0.930948	1.368396

H	-4.899594	2.063546	0.218510
H	-4.775697	-0.970491	-0.158672
H	-7.182900	-0.974201	0.511863
H	-6.141659	-0.411749	1.830626
H	-7.086033	0.753904	0.883459
H	-6.507903	-0.598970	-1.910618
H	-5.040396	0.302257	-2.318761
H	-6.433730	1.150803	-1.642090
H	4.007947	-2.084851	-3.001726
H	4.114616	-3.147088	-1.588760
H	2.525712	-2.703347	-2.233510
H	5.038590	-0.248261	-0.146790

Conformer 31:

C	-1.778435	-1.315336	0.228731
C	-1.886787	-0.424920	-0.870989
C	-0.663253	-0.071358	-1.542761
C	0.614118	-0.560664	-1.158230
C	0.663075	-1.404898	-0.055814
C	-0.509988	-1.802525	0.650158
C	1.814429	-0.247754	-2.033242
C	3.107760	0.276004	-1.364571
O	1.825277	-1.955677	0.394108
C	2.860558	1.567434	-0.550261
C	2.460682	1.348120	0.895934
C	2.552333	0.133066	1.467519
C	2.980797	-1.104293	0.728700
C	1.983948	2.549056	1.701380
H	3.787726	0.527273	-2.187707
C	-0.431160	-2.678382	1.793319
C	3.017092	3.693282	1.722383
C	0.612014	3.060571	1.214348
C	-3.154961	0.122469	-1.382669
O	-3.137065	0.879876	-2.382772
O	-2.863425	-1.699916	0.899595
O	-0.704762	0.740674	-2.593648
O	-1.428681	-3.058981	2.441456
C	-4.504880	-0.158570	-0.763217
C	-4.815538	0.716278	0.484675
C	-4.890152	2.210619	0.138041
C	-6.122352	0.233494	1.131069
C	3.840097	-0.780103	-0.508210
C	4.229006	-2.038201	-1.282418
O	5.095475	-0.250920	-0.036796
H	2.059605	-1.144066	-2.616260
H	1.499720	0.499614	-2.762883
H	3.767559	2.186715	-0.566780
H	2.096478	2.171121	-1.054808
H	2.271143	0.005173	2.511413
H	3.560049	-1.761186	1.383711
H	1.854508	2.202710	2.735017
H	0.566582	-3.020697	2.095521
H	2.681379	4.490723	2.393776
H	3.994173	3.346603	2.076624
H	3.151791	4.136456	0.729644
H	0.257697	3.864362	1.868788
H	-0.135921	2.261770	1.222466
H	0.665582	3.464629	0.197677
H	-2.548278	-2.318211	1.636787
H	-1.688860	0.970385	-2.734466
H	-5.242145	0.060259	-1.542879
H	-4.580294	-1.210700	-0.484196
H	-4.008383	0.564512	1.211167
H	-5.091438	2.801693	1.038560
H	-3.960343	2.580297	-0.305334
H	-5.696119	2.407303	-0.579356
H	-6.358299	0.827261	2.021223
H	-6.052023	-0.816462	1.434350
H	-6.964289	0.329977	0.434000
H	4.819365	-1.764295	-2.162358
H	4.848002	-2.675748	-0.644322

H	3.358648	-2.613119	-1.600857
H	4.921425	0.444872	0.612679

Conformer 32:

C	1.632077	1.303975	0.655900
C	1.952867	0.320918	-0.314804
C	0.889592	-0.098523	-1.188762
C	-0.432256	0.417555	-1.121150
C	-0.697330	1.353290	-0.128765
C	0.309725	1.818067	0.765680
C	-1.433609	0.022744	-2.191315
C	-2.838167	-0.457837	-1.754463
O	-1.923219	1.929347	0.023892
C	-2.767406	-1.669371	-0.797101
C	-2.659391	-1.325748	0.678001
C	-2.852705	-0.066664	1.111865
C	-3.126117	1.094248	0.194876
C	-2.350401	-2.491054	1.610744
H	-3.339651	-0.786796	-2.672820
C	0.009077	2.790808	1.787308
C	-1.945699	-2.083519	3.034273
C	-3.521721	-3.499248	1.658900
C	3.291030	-0.280046	-0.486802
O	3.461552	-1.138222	-1.382279
O	2.563774	1.754487	1.495644
O	1.131946	-1.005708	-2.131096
O	0.856619	3.232148	2.591282
C	4.464778	0.109958	0.389087
C	5.818446	-0.584156	0.126824
C	5.791483	-2.097944	0.398614
C	6.415706	-0.257116	-1.252440
C	-3.722755	0.657010	-1.155476
C	-3.943850	1.840002	-2.096027
O	-5.049322	0.153191	-0.900509
H	-1.550889	0.866426	-2.882595
H	-0.984109	-0.778657	-2.779194
H	-3.653638	-2.299851	-0.947631
H	-1.916198	-2.304772	-1.075358
H	-2.769393	0.171548	2.168058
H	-3.821380	1.796487	0.663614
H	-1.493308	-3.016508	1.162477
H	-1.025089	3.153178	1.846045
H	-1.640460	-2.969010	3.601055
H	-1.107140	-1.380150	3.033094
H	-2.779080	-1.621718	3.576171
H	-3.257892	-4.355996	2.287786
H	-3.780191	-3.885578	0.668753
H	-4.415725	-3.030988	2.087468
H	2.112169	2.429228	2.099337
H	2.113328	-1.254128	-2.037540
H	4.582900	1.197888	0.312705
H	4.161975	-0.045637	1.431889
H	6.490383	-0.140128	0.875991
H	6.806241	-2.509787	0.356307
H	5.389205	-2.314397	1.395843
H	5.180465	-2.625084	-0.338386
H	7.440386	-0.639455	-1.323116
H	6.455575	0.826025	-1.420156
H	5.829506	-0.706143	-2.058045
H	-4.347518	1.485079	-3.049413
H	-4.675091	2.521335	-1.651170
H	-3.024765	2.396826	-2.281994
H	-5.011663	-0.485287	-0.174450

Conformer 35:

C	-1.658402	-1.286812	0.515878
C	-1.900863	-0.373466	-0.541227
C	-0.776405	-0.020298	-1.365744
C	0.537700	-0.516012	-1.154169
C	0.726217	-1.370981	-0.074791
C	-0.347901	-1.780720	0.767223

C	1.619259	-0.195836	-2.169838
C	2.982064	0.332668	-1.662017
O	1.938629	-1.920194	0.218724
C	2.828528	1.613770	-0.810686
C	2.603624	1.383925	0.673325
C	2.767518	0.164405	1.218086
C	3.119812	-1.060769	0.419337
C	2.214759	2.614290	1.484731
H	3.553932	0.595101	-2.560434
C	-0.128895	-2.679700	1.873781
C	1.696050	2.313220	2.897753
C	3.374535	3.634777	1.549928
C	-3.217441	0.215885	-0.847558
O	-3.332720	0.974435	-1.836988
O	-2.656852	-1.691962	1.299921
O	-0.949988	0.803853	-2.395574
O	-1.038690	-3.074334	2.632576
C	-4.450565	-0.069614	-0.013263
C	-5.697959	0.747596	-0.391296
C	-6.946395	0.108353	0.237747
C	-5.567658	2.219527	0.032512
C	3.821023	-0.722464	-0.909097
C	4.123307	-1.972261	-1.733473
O	5.120176	-0.185529	-0.588877
H	1.795027	-1.089743	-2.780933
H	1.214414	0.552270	-2.852784
H	3.720772	2.240744	-0.938695
H	1.999284	2.216088	-1.204755
H	2.600003	0.007500	2.279492
H	3.778631	-1.716744	0.995422
H	1.395376	3.095129	0.928815
H	0.898368	-3.026999	2.042504
H	1.342032	3.236501	3.367576
H	0.862987	1.603509	2.882383
H	2.483763	1.902716	3.539947
H	3.056987	4.534684	2.087001
H	3.711861	3.946379	0.557169
H	4.232099	3.209725	2.084801
H	-2.255580	-2.320283	1.983472
H	-1.937835	1.042877	-2.403061
H	-4.651021	-1.145990	-0.101673
H	-4.204109	0.066863	1.046032
H	-5.806808	0.717607	-1.481993
H	-6.876339	0.094270	1.332888
H	-7.848129	0.670637	-0.028690
H	-7.083990	-0.925096	-0.100632
H	-6.459586	2.786791	-0.256375
H	-4.702668	2.696947	-0.435839
H	-5.461595	2.302634	1.122242
H	4.599106	-1.687881	-2.677185
H	4.821106	-2.607715	-1.180216
H	3.225246	-2.553129	-1.946506
H	5.020759	0.507566	0.078911

Conformer 36:

C	1.827331	-1.215453	-0.435722
C	1.941164	-0.562842	0.819098
C	0.722361	-0.363842	1.559651
C	-0.556709	-0.769839	1.093186
C	-0.612128	-1.373745	-0.157497
C	0.557364	-1.615507	-0.936453
C	-1.745832	-0.647300	2.029776
C	-3.067591	-0.041484	1.500610
O	-1.775599	-1.823636	-0.705071
C	-2.903488	1.400052	0.973646
C	-2.450342	1.509939	-0.467890
C	-2.498084	0.446700	-1.285479
C	-2.933867	-0.917644	-0.842837
C	-1.991083	2.890999	-0.917578
H	-3.731937	-0.000191	2.375983
C	0.472271	-2.242066	-2.232559

C	-1.300784	2.922403	-2.288165
C	-3.157991	3.904834	-0.886911
C	3.209888	-0.113110	1.416004
O	3.199661	0.417496	2.553068
O	2.908210	-1.452870	-1.177898
O	0.771115	0.212978	2.756073
O	1.466174	-2.481560	-2.950435
C	4.551634	-0.229128	0.729294
C	4.813549	0.884672	-0.324042
C	4.852973	2.282475	0.311102
C	6.120345	0.580486	-1.071285
C	-3.777194	-0.912316	0.443533
C	-4.096307	-2.332826	0.919147
O	-5.006641	-0.271710	0.040512
H	-1.958974	-1.641239	2.444133
H	-1.429130	-0.040193	2.878972
H	-3.865186	1.918559	1.065967
H	-2.201189	1.945335	1.618293
H	-2.180471	0.525411	-2.320799
H	-3.513991	-1.409753	-1.628249
H	-1.254498	3.225787	-0.170614
H	-0.526967	-2.518048	-2.592005
H	-0.918321	3.928534	-2.488972
H	-0.456706	2.226980	-2.334769
H	-1.996690	2.670315	-3.096584
H	-2.801657	4.900721	-1.171482
H	-3.609967	3.986839	0.105763
H	-3.942300	3.609140	-1.592919
H	2.588603	-1.912032	-2.021929
H	1.755007	0.416741	2.932048
H	5.301191	-0.150715	1.523868
H	4.644301	-1.202138	0.244261
H	3.996817	0.854937	-1.054959
H	5.022139	3.045876	-0.456632
H	3.920756	2.528603	0.829079
H	5.665268	2.358019	1.044204
H	6.321585	1.345194	-1.829808
H	6.073702	-0.390795	-1.575116
H	6.973022	0.564205	-0.380768
H	-4.680943	-2.298722	1.847442
H	-4.689709	-2.847657	0.156824
H	-3.199234	-2.925078	1.104866
H	-5.672754	-0.432980	0.722149

Conformer 38:

C	1.691650	1.272975	0.419528
C	1.842417	0.605197	-0.823516
C	0.638737	0.339637	-1.567614
C	-0.659262	0.704447	-1.119271
C	-0.749235	1.327198	0.119539
C	0.402356	1.628993	0.904200
C	-1.839880	0.514362	-2.054405
C	-3.116140	-0.170556	-1.510233
O	-1.934672	1.743650	0.646298
C	-2.821755	-1.574397	-0.935709
C	-2.449687	-1.612617	0.534221
C	-2.597160	-0.528905	1.317733
C	-3.061290	0.806216	0.802003
C	-1.942527	-2.955318	1.045876
H	-3.779970	-0.295153	-2.374456
C	0.281338	2.276017	2.187507
C	-0.458601	-3.160921	0.663891
C	-2.140929	-3.183351	2.551769
C	3.133951	0.203022	-1.406159
O	3.152839	-0.355426	-2.529603
O	2.755859	1.565121	1.166095
O	0.719318	-0.263495	-2.748999
O	1.258412	2.564968	2.909933
C	4.466545	0.404421	-0.721637
C	4.788948	-0.675567	0.350160
C	4.913402	-2.078295	-0.262744

C	6.072498	-0.283375	1.096713
C	-3.895749	0.683764	-0.486956
C	-4.324921	2.047989	-1.023756
O	-5.134506	0.032605	-0.140765
H	-2.115101	1.492826	-2.466984
H	-1.491291	-0.074630	-2.903857
H	-3.699588	-2.222609	-1.073499
H	-2.027594	-2.049204	-1.525305
H	-2.349224	-0.574497	2.374459
H	-3.670838	1.314565	1.554363
H	-2.521509	-3.726674	0.515596
H	-0.730914	2.522190	2.532123
H	-0.132691	-4.170007	0.938149
H	-0.285732	-3.035219	-0.409029
H	0.175968	-2.442897	1.194619
H	-1.851833	-4.205911	2.815692
H	-3.184481	-3.038823	2.849955
H	-1.519682	-2.507225	3.149592
H	2.411728	2.025192	1.999662
H	1.712683	-0.428005	-2.915324
H	5.219945	0.353994	-1.514830
H	4.505159	1.389081	-0.253169
H	3.968822	-0.682230	1.077897
H	5.122758	-2.818539	0.517650
H	4.000324	-2.386501	-0.781466
H	5.732937	-2.117327	-0.990580
H	6.315706	-1.023451	1.867267
H	5.966528	0.690829	1.585616
H	6.925337	-0.227018	0.408560
H	-4.892642	1.920030	-1.950669
H	-4.976888	2.533237	-0.291474
H	-3.474117	2.703431	-1.213260
H	-4.940032	-0.764459	0.372194

Conformer 40:

C	-1.702149	-1.289326	0.362628
C	-1.848213	-0.559719	-0.845609
C	-0.641048	-0.254036	-1.568370
C	0.656080	-0.634510	-1.131012
C	0.741343	-1.322112	0.073983
C	-0.414441	-1.665996	0.835105
C	1.835459	-0.392470	-2.056779
C	3.132165	0.226476	-1.482956
O	1.923117	-1.764477	0.586726
C	2.905631	1.613363	-0.845845
C	2.474648	1.596393	0.604983
C	2.577099	0.480480	1.342805
C	3.048975	-0.830666	0.789763
C	1.980783	2.927314	1.156441
H	3.791458	0.363965	-2.352279
C	-0.298256	-2.373741	2.086241
C	0.523194	3.199544	0.719968
C	2.118327	3.077578	2.678559
C	-3.137616	-0.132723	-1.414361
O	-3.153372	0.478102	-2.510377
O	-2.769420	-1.622767	1.087393
O	-0.718206	0.403508	-2.720967
O	-1.278595	-2.703703	2.786687
C	-4.472625	-0.367998	-0.745220
C	-4.798564	0.657021	0.378124
C	-4.922055	2.088474	-0.164518
C	-6.083919	0.227582	1.100675
C	3.881866	-0.692947	-0.496762
C	4.253123	-2.059773	-1.079977
O	5.088035	-0.036114	-0.051683
H	2.088039	-1.342250	-2.545789
H	1.490644	0.263480	-2.857323
H	3.839156	2.189803	-0.894239
H	2.176419	2.172742	-1.445348
H	2.298501	0.479402	2.392647
H	3.652561	-1.363644	1.529359

H	2.607553	3.703533	0.691101
H	0.713115	-2.629291	2.426260
H	0.214852	4.205076	1.026653
H	0.395264	3.128252	-0.364424
H	-0.156655	2.478586	1.187244
H	1.847401	4.094858	2.980458
H	3.143234	2.884789	3.011045
H	1.451551	2.392709	3.214610
H	-2.427651	-2.123226	1.898663
H	-1.710840	0.573521	-2.883487
H	-5.223384	-0.278005	-1.537455
H	-4.512628	-1.374798	-0.326573
H	-3.980417	0.627882	1.107502
H	-5.133929	2.788870	0.651179
H	-4.007583	2.422480	-0.664423
H	-5.739571	2.163317	-0.891893
H	-6.329925	0.928508	1.906172
H	-5.978482	-0.769527	1.541174
H	-6.934787	0.204682	0.408111
H	4.825792	-1.933010	-2.007692
H	4.874945	-2.604914	-0.362718
H	3.378642	-2.672893	-1.302130
H	5.755410	-0.120554	-0.745822

Conformer 42:

C	1.751633	-1.268004	-0.358021
C	1.893637	-0.410567	0.767046
C	0.685124	-0.066361	1.474068
C	-0.599372	-0.567777	1.129866
C	-0.678322	-1.393172	0.016065
C	0.472266	-1.757422	-0.743254
C	-1.771480	-0.281696	2.050987
C	-3.092814	0.238898	1.436702
O	-1.849587	-1.949881	-0.401715
C	-2.887078	1.545656	0.635396
C	-2.536565	1.353789	-0.827368
C	-2.636466	0.147196	-1.415289
C	-3.025552	-1.106256	-0.682019
C	-2.100851	2.572595	-1.629186
H	-3.745922	0.469742	2.287117
C	0.355863	-2.599058	-1.908851
C	-3.143777	3.707492	-1.591417
C	-0.715917	3.088367	-1.185216
C	3.170245	0.120706	1.271007
O	3.162125	0.921983	2.237731
O	2.806370	-1.612093	-1.094491
O	0.748794	0.744232	2.524077
O	1.328617	-2.945586	-2.611542
C	4.541347	-0.240238	0.735397
C	5.256200	0.969153	0.073064
C	6.715944	0.603898	-0.231180
C	4.533684	1.453231	-1.193478
C	-3.844013	-0.811276	0.589395
C	-4.192381	-2.085846	1.355910
O	-5.120607	-0.288165	0.171111
H	-1.986925	-1.190225	2.626804
H	-1.439131	0.456992	2.781557
H	-3.798925	2.155185	0.693989
H	-2.111506	2.148908	1.122613
H	-2.391441	0.039139	-2.470473
H	-3.620283	-1.759417	-1.326796
H	-2.007470	2.245155	-2.672812
H	-0.649431	-2.946732	-2.177711
H	-2.840777	4.518901	-2.261618
H	-4.130776	3.357874	-1.913876
H	-3.244088	4.132994	-0.586942
H	-0.394443	3.907803	-1.837200
H	0.038522	2.297424	-1.237623
H	-0.733932	3.472515	-0.159622
H	2.465077	-2.211712	-1.836550
H	1.732585	1.001408	2.623117

H	5.120270	-0.531055	1.620567
H	4.509647	-1.081484	0.047739
H	5.251177	1.785092	0.806929
H	7.248334	1.458410	-0.663678
H	7.250827	0.299421	0.675452
H	6.773976	-0.222854	-0.950003
H	5.054933	2.314121	-1.626738
H	3.504445	1.764310	-0.982409
H	4.494490	0.663466	-1.951922
H	-4.752636	-1.832725	2.261391
H	-4.828256	-2.718934	0.730081
H	-3.305425	-2.657064	1.632361
H	-4.976763	0.419562	-0.472856

Conformer 43:

C	1.641098	-1.375268	-0.420039
C	1.864324	-0.348321	0.531413
C	0.721881	0.102920	1.280092
C	-0.592027	-0.404049	1.092281
C	-0.758612	-1.382370	0.118509
C	0.333799	-1.889314	-0.644328
C	-1.686883	0.044779	2.045117
C	-3.063153	0.470043	1.483101
O	-1.963089	-1.965798	-0.138904
C	-2.975408	1.658551	0.500531
C	-2.670990	1.280039	-0.932183
C	-2.784194	0.008984	-1.348767
C	-3.143278	-1.129669	-0.443263
C	-2.286140	2.385265	-1.904523
H	-3.643918	0.803474	2.352197
C	0.135021	-2.905656	-1.647822
C	-3.356533	3.492609	-1.979779
C	-0.900457	2.983972	-1.585854
C	3.178026	0.262898	0.804317
O	3.276049	1.127016	1.705169
O	2.655245	-1.872192	-1.127631
O	0.878338	1.035610	2.215689
O	1.059833	-3.390184	-2.333266
C	4.429313	-0.123260	0.040228
C	5.674924	0.721823	0.358068
C	6.932472	0.007094	-0.162243
C	5.570968	2.138249	-0.230182
C	-3.857359	-0.686040	0.851149
C	-4.102309	-1.862370	1.800815
O	-5.127309	-0.113228	0.482484
H	-1.843306	-0.751056	2.784855
H	-1.297221	0.892360	2.610374
H	-3.934379	2.190530	0.507814
H	-2.228530	2.373087	0.867467
H	-2.582180	-0.243973	-2.388169
H	-3.783606	-1.847935	-0.968351
H	-2.220607	1.922025	-2.897847
H	-0.891062	-3.261745	-1.804439
H	-3.099825	4.212356	-2.764714
H	-4.344035	3.078175	-2.208223
H	-3.430877	4.046960	-1.037669
H	-0.622531	3.717566	-2.350707
H	-0.128030	2.208874	-1.562450
H	-0.892306	3.499002	-0.619036
H	2.265644	-2.572579	-1.744693
H	1.867540	1.268518	2.220754
H	4.616460	-1.184417	0.253608
H	4.211665	-0.104240	-1.033846
H	5.755362	0.812851	1.447865
H	7.832921	0.588744	0.064063
H	7.050435	-0.983248	0.292540
H	6.890539	-0.128480	-1.250511
H	6.461144	2.727415	0.017437
H	4.699262	2.671284	0.158887
H	5.493647	2.100136	-1.324802
H	-4.593573	-1.506583	2.711848

H	-4.761526	-2.598450	1.322556
H	-3.182790	-2.384127	2.069900
H	-5.723752	-0.821764	0.205132

Conformer 45:

C	1.681906	1.099110	0.626873
C	1.923997	0.098223	-0.347796
C	0.817782	-0.268795	-1.190685
C	-0.467847	0.329109	-1.104443
C	-0.653951	1.291137	-0.118091
C	0.396801	1.695429	0.755992
C	-1.504754	-0.017317	-2.158605
C	-2.947665	-0.361360	-1.718111
O	-1.835508	1.947971	0.053141
C	-3.015997	-1.574973	-0.766797
C	-2.805607	-1.255599	0.699243
C	-2.869724	0.009567	1.142408
C	-3.088353	1.189948	0.244446
C	-2.565715	-2.450779	1.612747
H	-3.469621	-0.638110	-2.645658
C	0.174161	2.683594	1.782709
C	-2.105427	-2.090726	3.032063
C	-3.810601	-3.365631	1.673795
C	3.222270	-0.570302	-0.550444
O	3.318881	-1.470402	-1.415621
O	2.652736	1.484229	1.454515
O	0.985902	-1.201756	-2.124532
O	1.061176	3.070909	2.572062
C	4.453613	-0.219414	0.261632
C	5.749302	-0.920638	-0.181678
C	6.281282	-0.354203	-1.508038
C	6.808648	-0.800105	0.925679
C	-3.718515	0.829076	-1.111567
C	-3.811637	2.046799	-2.035876
O	-5.055062	0.408239	-0.763876
H	-1.548066	0.808852	-2.880168
H	-1.125378	-0.873092	-2.718710
H	-3.999770	-2.047588	-0.871735
H	-2.281906	-2.327698	-1.084051
H	-2.726157	0.239288	2.193864
H	-3.729104	1.930202	0.731351
H	-1.759235	-3.035923	1.143895
H	-0.834585	3.108489	1.859900
H	-1.863862	-3.002976	3.587344
H	-1.212537	-1.457581	3.022152
H	-2.889976	-1.566871	3.590154
H	-3.606712	-4.240980	2.299851
H	-4.107476	-3.728319	0.685506
H	-4.661579	-2.827880	2.107042
H	2.255107	2.186796	2.064482
H	1.948062	-1.517136	-2.039190
H	4.582535	0.869015	0.260760
H	4.222470	-0.457841	1.308698
H	5.526649	-1.983721	-0.332447
H	7.196192	-0.874622	-1.812963
H	5.550307	-0.464706	-2.313515
H	6.525404	0.711397	-1.405842
H	7.737028	-1.302934	0.633065
H	6.464660	-1.251366	1.863565
H	7.051341	0.250420	1.130378
H	-4.244395	1.756751	-3.001947
H	-4.460737	2.801245	-1.580024
H	-2.840438	2.506773	-2.223431
H	-5.587036	0.383701	-1.570556

Conformer 46:

C	1.607541	1.401261	0.584403
C	1.908362	0.344831	-0.312777
C	0.823668	-0.149245	-1.118736
C	-0.500989	0.359527	-1.052184
C	-0.744817	1.373821	-0.133302

C	0.284838	1.914611	0.690262
C	-1.522122	-0.132594	-2.062866
C	-2.930964	-0.546700	-1.574617
O	-1.967560	1.958204	0.008487
C	-2.898009	-1.692364	-0.539378
C	-2.706527	-1.249556	0.895037
C	-2.861469	0.035950	1.248223
C	-3.162983	1.132746	0.273241
C	-2.388335	-2.309551	1.939474
H	-3.442255	-0.926550	-2.471049
C	0.005244	2.962598	1.640847
C	-3.453458	-3.423682	1.981921
C	-0.978105	-2.906437	1.752495
C	3.245734	-0.259702	-0.475222
O	3.399130	-1.184046	-1.305773
O	2.559254	1.924416	1.356934
O	1.048459	-1.124463	-1.995510
O	0.872627	3.474240	2.379684
C	4.439934	0.204127	0.334824
C	5.789991	-0.504744	0.094238
C	5.777605	-1.991529	0.488406
C	6.350063	-0.288936	-1.322050
C	-3.779190	0.627359	-1.043359
C	-3.969930	1.763853	-2.052375
O	-5.078069	0.138671	-0.645218
H	-1.633848	0.635624	-2.838963
H	-1.088494	-0.996205	-2.568910
H	-3.844643	-2.244126	-0.591280
H	-2.115501	-2.407901	-0.819925
H	-2.743410	0.333188	2.288640
H	-3.849004	1.861902	0.712748
H	-2.401870	-1.804174	2.914028
H	-1.030692	3.318588	1.704666
H	-3.252364	-4.106100	2.814938
H	-4.458194	-3.009635	2.116651
H	-3.451437	-4.019599	1.062369
H	-0.753401	-3.604083	2.566877
H	-0.212088	-2.124629	1.754297
H	-0.892342	-3.461733	0.812024
H	2.118830	2.642953	1.917384
H	2.033757	-1.358891	-1.909870
H	4.550273	1.282584	0.166806
H	4.165479	0.133392	1.394468
H	6.478785	0.000579	0.787189
H	6.792764	-2.403005	0.451725
H	5.403478	-2.127348	1.510485
H	5.149139	-2.578136	-0.186365
H	7.375141	-0.671229	-1.387699
H	6.378604	0.777088	-1.578482
H	5.746479	-0.804770	-2.072950
H	-4.388862	1.373201	-2.988633
H	-4.668504	2.500554	-1.643007
H	-3.036866	2.278129	-2.286592
H	-5.616174	0.020289	-1.439439

Conformer 47:

C	-1.571734	-1.216847	0.449041
C	-1.843530	-0.096259	-0.376068
C	-0.745601	0.425934	-1.144536
C	0.559717	-0.133875	-1.130475
C	0.773949	-1.220356	-0.289805
C	-0.266913	-1.780389	0.506747
C	1.591065	0.396446	-2.110800
C	3.015628	0.723989	-1.603211
O	1.975747	-1.856481	-0.203645
C	3.024356	1.781789	-0.479965
C	2.834462	1.240021	0.920523
C	2.952143	-0.071941	1.175662
C	3.203001	-1.097309	0.111000
C	2.559418	2.284867	1.993833
H	3.535631	1.155764	-2.470683

C	-0.015062	-2.895586	1.386055
C	1.079539	2.730313	1.963959
C	2.960385	1.860944	3.414359
C	-3.163907	0.548346	-0.497225
O	-3.286872	1.559776	-1.225261
O	-2.533429	-1.749327	1.202462
O	-0.940478	1.478023	-1.935753
O	-0.892396	-3.421678	2.102476
C	-4.387655	0.040748	0.239812
C	-5.704957	0.751650	-0.115174
C	-6.204974	0.363648	-1.516119
C	-6.767842	0.435024	0.949245
C	3.826293	-0.515088	-1.169817
C	3.969930	-1.577985	-2.263301
O	5.144111	-0.102119	-0.749044
H	1.671074	-0.315183	-2.942710
H	1.185113	1.309986	-2.547452
H	3.986932	2.310458	-0.489224
H	2.262456	2.542385	-0.692355
H	2.848607	-0.453840	2.187214
H	3.864979	-1.883521	0.483879
H	3.166760	3.163986	1.728989
H	1.008069	-3.291183	1.414072
H	0.917136	3.560766	2.659819
H	0.767141	3.062988	0.969424
H	0.425505	1.903834	2.263136
H	2.834535	2.701124	4.105504
H	4.005104	1.537145	3.457843
H	2.333835	1.041379	3.784090
H	-2.114724	-2.517025	1.711252
H	-1.914312	1.743799	-1.817447
H	-4.475685	-1.040252	0.081012
H	-4.173784	0.133794	1.313170
H	-5.520991	1.832674	-0.108669
H	-7.136080	0.889724	-1.755171
H	-5.472183	0.614969	-2.287616
H	-6.410191	-0.713566	-1.571353
H	-7.712050	0.940998	0.719458
H	-6.448478	0.758747	1.946639
H	-6.972585	-0.642075	0.997825
H	4.394349	-1.131967	-3.171947
H	4.646221	-2.366492	-1.918022
H	3.017918	-2.041546	-2.525606
H	5.679991	0.058061	-1.537405

Conformer 50:

C	-1.748129	-0.780462	0.575942
C	-1.883240	0.331787	-0.294822
C	-0.746022	0.653572	-1.117148
C	0.470362	-0.080107	-1.099321
C	0.557328	-1.140156	-0.204616
C	-0.530570	-1.513400	0.638063
C	1.537186	0.252277	-2.127647
C	3.006086	0.408495	-1.667077
O	1.663725	-1.928516	-0.105049
C	3.188259	1.513311	-0.605673
C	2.961975	1.078182	0.826212
C	2.907384	-0.222118	1.152590
C	2.990123	-1.327486	0.143101
C	2.858539	2.206380	1.843993
H	3.556314	0.718237	-2.567418
C	-0.412284	-2.613185	1.563306
C	1.453353	2.849703	1.814268
C	3.232083	1.806995	3.279031
C	-3.088735	1.170989	-0.399481
O	-3.090897	2.146995	-1.188169
O	-2.756843	-1.152776	1.362913
O	-0.817420	1.679797	-1.958978
O	-1.336436	-2.983053	2.318109
C	-4.358408	0.923083	0.382559
C	-5.267553	-0.177960	-0.234474

C	-6.436585	-0.464153	0.719542
C	-5.776176	0.208163	-1.631194
C	3.652461	-0.904996	-1.180119
C	3.621995	-2.034368	-2.214445
O	5.024772	-0.654577	-0.808180
H	1.497988	-0.506515	-2.919861
H	1.246623	1.188457	-2.606370
H	4.213577	1.902719	-0.662131
H	2.532429	2.359252	-0.846961
H	2.778309	-0.531996	2.185607
H	3.549442	-2.175459	0.547399
H	3.573946	2.977957	1.520354
H	0.547762	-3.143558	1.592370
H	1.422656	3.728840	2.467251
H	1.166152	3.171681	0.808697
H	0.699393	2.137778	2.167880
H	3.239943	2.692030	3.924044
H	4.223069	1.344182	3.322967
H	2.507932	1.103121	3.704652
H	-2.429782	-1.944456	1.903039
H	-1.743539	2.087263	-1.829443
H	-4.121189	0.648551	1.411530
H	-4.901015	1.874416	0.379835
H	-4.673702	-1.095738	-0.319086
H	-7.086922	-1.246961	0.313511
H	-6.078282	-0.797478	1.699317
H	-7.049875	0.433394	0.869448
H	-6.398938	-0.592859	-2.045040
H	-4.957746	0.393358	-2.333744
H	-6.385275	1.119358	-1.589336
H	4.078424	-1.699465	-3.154766
H	4.194601	-2.888101	-1.838168
H	2.609527	-2.377297	-2.431967
H	5.556373	-0.610928	-1.614306

Conformer 51:

C	1.673103	-1.132315	-0.656240
C	1.855926	-0.631146	0.661425
C	0.667319	-0.464469	1.460111
C	-0.636858	-0.812163	1.015081
C	-0.755657	-1.287230	-0.284302
C	0.373939	-1.455577	-1.138167
C	-1.788113	-0.763381	2.003261
C	-3.098817	-0.055601	1.585072
O	-1.948556	-1.671083	-0.818652
C	-2.858806	1.413492	1.171099
C	-2.538636	1.633663	-0.295028
C	-2.687374	0.645455	-1.195841
C	-3.101949	-0.753081	-0.826879
C	-2.081717	3.040582	-0.660706
H	-3.734521	-0.051767	2.478924
C	0.216755	-1.930526	-2.490964
C	-0.592115	3.243207	-0.299722
C	-2.333241	3.435749	-2.123432
C	3.154848	-0.299650	1.268483
O	3.184086	0.197571	2.421183
O	2.707739	-1.285405	-1.480472
O	0.769374	0.017644	2.693484
O	1.169629	-2.092891	-3.282097
C	4.507127	-0.533765	0.625412
C	5.250211	0.792947	0.308545
C	6.693884	0.488547	-0.115679
C	4.526485	1.628071	-0.758776
C	-3.893429	-0.805433	0.493580
C	-4.271369	-2.234340	0.879293
O	-5.158520	-0.152469	0.266408
H	-2.025326	-1.790400	2.307235
H	-1.423747	-0.267523	2.903949
H	-3.746991	2.015991	1.411651
H	-2.056625	1.838269	1.787316
H	-2.477231	0.821414	-2.247071

H	-3.725120	-1.186488	-1.614174
H	-2.662994	3.728235	-0.027682
H	-0.802531	-2.158683	-2.826782
H	-0.301268	4.286689	-0.461680
H	-0.381765	2.997212	0.745306
H	0.043538	2.611865	-0.929981
H	-2.079086	4.490035	-2.274210
H	-3.381557	3.296255	-2.406964
H	-1.713656	2.852082	-2.813299
H	2.338543	-1.641284	-2.354354
H	1.762489	0.207150	2.841347
H	5.088248	-1.073280	1.383291
H	4.440572	-1.150302	-0.267410
H	5.280118	1.373166	1.239585
H	7.246681	1.415365	-0.305715
H	7.230912	-0.069316	0.659676
H	6.717016	-0.107494	-1.036433
H	5.068431	2.561468	-0.947518
H	3.510195	1.895009	-0.448139
H	4.453016	1.080997	-1.705225
H	-4.810632	-2.231034	1.831686
H	-4.935637	-2.649026	0.115377
H	-3.399156	-2.883475	0.964720
H	-5.001025	0.703715	-0.156001

Conformer 52:

C	1.651148	-1.201150	-0.552340
C	1.876957	-0.118211	0.334668
C	0.754921	0.324497	1.118304
C	-0.531120	-0.275479	1.057584
C	-0.700203	-1.321697	0.157602
C	0.366752	-1.803761	-0.655132
C	-1.589234	0.166793	2.053144
C	-3.020227	0.473689	1.550419
O	-1.879587	-1.990536	0.022594
C	-3.059195	1.598324	0.492608
C	-2.828517	1.141356	-0.931527
C	-2.894615	-0.158355	-1.259786
C	-3.127074	-1.253713	-0.264585
C	-2.573947	2.199595	-1.994993
H	-3.561588	0.835283	2.436662
C	0.161302	-2.878136	-1.595318
C	-3.708597	3.241410	-2.063983
C	-1.206336	2.889979	-1.813152
C	3.173727	0.562279	0.505117
O	3.256131	1.535276	1.289092
O	2.637619	-1.660499	-1.321671
O	0.907393	1.336037	1.969218
O	1.062969	-3.336183	-2.327994
C	4.420167	0.137344	-0.246038
C	5.709065	0.873660	0.158176
C	6.211862	0.430035	1.541472
C	6.790399	0.650209	-0.911345
C	-3.783599	-0.765737	1.038960
C	-3.902245	-1.892335	2.069800
O	-5.110420	-0.374194	0.625714
H	-1.652415	-0.590881	2.844923
H	-1.218260	1.068409	2.542524
H	-4.041852	2.084495	0.528404
H	-2.329445	2.371266	0.762809
H	-2.750054	-0.467228	-2.293433
H	-3.759105	-2.036548	-0.692487
H	-2.549120	1.675925	-2.959618
H	-0.847722	-3.305002	-1.656558
H	-3.547063	3.919372	-2.909147
H	-4.683654	2.760708	-2.195394
H	-3.750588	3.853653	-1.156189
H	-1.023201	3.586241	-2.639050
H	-0.391189	2.159706	-1.797906
H	-1.161527	3.466157	-0.882467
H	2.249915	-2.413343	-1.875346

H	1.872720	1.640402	1.876308
H	4.544954	-0.946813	-0.143211
H	4.211434	0.279342	-1.315002
H	5.487436	1.946539	0.206433
H	7.122319	0.974514	1.816188
H	5.465089	0.615128	2.318244
H	6.454071	-0.640891	1.542176
H	7.714538	1.175832	-0.646736
H	6.467431	1.014384	-1.893507
H	7.033125	-0.415146	-1.013940
H	-4.352966	-1.512746	2.995791
H	-4.545857	-2.683508	1.672120
H	-2.937535	-2.336329	2.319120
H	-5.661070	-0.281711	1.414730

Conformer 53:

C	1.655260	1.201781	0.553174
C	1.878332	0.114743	-0.329207
C	0.754400	-0.330537	-1.108787
C	-0.531433	0.271216	-1.049914
C	-0.696806	1.322250	-0.155157
C	0.371723	1.806398	0.654846
C	-1.589438	-0.175314	-2.044454
C	-3.023738	-0.476806	-1.551874
O	-1.874880	1.994283	-0.020501
C	-3.075724	-1.597636	-0.490490
C	-2.838823	-1.139800	0.931825
C	-2.898389	0.161381	1.256568
C	-3.124358	1.254175	0.256573
C	-2.584668	-2.195924	1.997174
H	-3.567821	-0.833963	-2.435161
C	0.168850	2.884710	1.590549
C	-3.718644	-3.238397	2.066368
C	-1.216732	-2.885944	1.815691
C	3.174445	-0.567408	-0.499267
O	3.254329	-1.544663	-1.277990
O	2.643298	1.663173	1.319369
O	0.904586	-1.346403	-1.954506
O	1.071469	3.345111	2.320749
C	4.423113	-0.138847	0.246179
C	5.710977	-0.876124	-0.159489
C	6.208671	-0.437837	-1.546341
C	6.795903	-0.647784	0.905345
C	-3.780738	0.765786	-1.052061
C	-3.887938	1.886181	-2.090665
O	-5.106048	0.296954	-0.734715
H	-1.645547	0.575840	-2.842989
H	-1.220044	-1.081940	-2.525420
H	-4.064943	-2.069638	-0.527677
H	-2.355232	-2.379761	-0.759174
H	-2.747939	0.472907	2.288783
H	-3.748931	2.044676	0.688557
H	-2.559773	-1.671207	2.961352
H	-0.839627	3.313084	1.650945
H	-3.556367	-3.916291	2.911443
H	-4.694273	-2.758803	2.197786
H	-3.760771	-3.850002	1.158312
H	-1.034020	-3.582502	2.641432
H	-0.401714	-2.155531	1.801121
H	-1.171674	-3.461495	0.884710
H	2.257600	2.418783	1.870269
H	1.869471	-1.651587	-1.861864
H	4.547100	0.944957	0.138782
H	4.217942	-0.276868	1.316377
H	5.489790	-1.949307	-0.202650
H	7.118470	-0.982913	-1.822038
H	5.459291	-0.626542	-2.319727
H	6.450255	0.633221	-1.552281
H	7.719386	-1.174008	0.639645
H	6.476539	-1.008152	1.890093
H	7.038435	0.418111	1.002774

H	-4.341159	1.498326	-3.008317
H	-4.527982	2.691606	-1.707600
H	-2.921335	2.332231	-2.329079
H	-5.672610	1.057559	-0.547165

Conformer 56:

C	1.827282	-0.758858	-0.626261
C	1.911158	0.343568	0.263336
C	0.753757	0.609765	1.077466
C	-0.434879	-0.167216	1.032598
C	-0.471614	-1.216358	0.121692
C	0.638831	-1.535725	-0.713647
C	-1.524745	0.110088	2.053013
C	-2.994312	0.216273	1.580301
O	-1.546409	-2.044341	-0.002467
C	-3.213445	1.335214	0.538510
C	-2.951747	0.930530	-0.896013
C	-2.838038	-0.361781	-1.240118
C	-2.891520	-1.489481	-0.255206
C	-2.877201	2.025095	-1.950578
H	-3.563619	0.488097	2.481010
C	0.572123	-2.624276	-1.657172
C	-4.154933	2.887560	-1.985350
C	-1.623668	2.909065	-1.785650
C	3.083799	1.224373	0.396079
O	3.040876	2.186783	1.200108
O	2.857685	-1.080958	-1.407133
O	0.778465	1.622383	1.937984
O	1.517876	-2.947389	-2.406720
C	4.370908	1.035362	-0.373818
C	5.311722	-0.042142	0.237260
C	6.500840	-0.272116	-0.707095
C	5.790571	0.340167	1.645518
C	-3.584081	-1.113486	1.066822
C	-3.520835	-2.256614	2.084362
O	-4.961052	-0.910488	0.683252
H	-1.464428	-0.657168	2.835725
H	-1.275225	1.050029	2.547264
H	-4.253410	1.678454	0.600623
H	-2.593426	2.200059	0.804305
H	-2.673059	-0.636160	-2.280417
H	-3.414742	-2.349686	-0.681463
H	-2.794628	1.520138	-2.921930
H	-0.366935	-3.189574	-1.705861
H	-4.112938	3.590161	-2.824762
H	-5.050439	2.268715	-2.104438
H	-4.268550	3.477927	-1.069264
H	-1.561273	3.631021	-2.607394
H	-0.709174	2.307705	-1.792371
H	-1.646963	3.478578	-0.850086
H	2.566079	-1.875841	-1.962959
H	1.689751	2.066807	1.825847
H	4.156206	0.769269	-1.409927
H	4.878650	2.005425	-0.349184
H	4.750019	-0.981578	0.301134
H	7.174180	-1.037286	-0.304960
H	6.165650	-0.603232	-1.695746
H	7.083300	0.648823	-0.836692
H	6.436525	-0.444710	2.054703
H	4.958231	0.485684	2.341030
H	6.367541	1.272742	1.624484
H	-3.999843	-1.954404	3.024504
H	-4.055778	-3.125918	1.688783
H	-2.498189	-2.563530	2.307871
H	-5.502889	-0.902506	1.483678

Conformer 57:

C	-1.795887	-1.311289	0.222855
C	-1.900170	-0.409235	-0.867578
C	-0.673790	-0.048559	-1.530336
C	0.603015	-0.537884	-1.144098

C	0.647874	-1.396955	-0.052231
C	-0.528958	-1.802461	0.643456
C	1.801208	-0.209373	-2.017615
C	3.115226	0.266721	-1.353755
O	1.805975	-1.955280	0.397840
C	2.940134	1.559944	-0.527697
C	2.486159	1.347663	0.900116
C	2.533007	0.132038	1.466909
C	2.963540	-1.105274	0.740159
C	2.026736	2.559725	1.697309
H	3.788538	0.499312	-2.191502
C	-0.454250	-2.688076	1.779192
C	3.104731	3.660579	1.756962
C	0.691299	3.128164	1.174403
C	-3.166193	0.142691	-1.378605
O	-3.145870	0.906366	-2.374118
O	-2.883399	-1.704290	0.884894
O	-0.712760	0.770518	-2.576184
O	-1.454532	-3.077811	2.417987
C	-4.517585	-0.139252	-0.762617
C	-4.825372	0.724812	0.493412
C	-4.893384	2.222711	0.161085
C	-6.134595	0.241405	1.134427
C	3.816763	-0.815711	-0.507475
C	4.141571	-2.097011	-1.281431
O	5.042140	-0.279726	0.036204
H	2.022479	-1.086509	-2.639487
H	1.490500	0.569750	-2.715066
H	3.899262	2.091142	-0.493878
H	2.243138	2.227646	-1.048661
H	2.225122	0.001469	2.502734
H	3.537763	-1.760489	1.400760
H	1.853507	2.212873	2.724458
H	0.542713	-3.029253	2.084830
H	2.784647	4.465927	2.427035
H	4.056358	3.266855	2.129074
H	3.282524	4.105477	0.771499
H	0.351747	3.946196	1.819344
H	-0.088897	2.360651	1.160507
H	0.788970	3.529574	0.159738
H	-2.570243	-2.330038	1.616966
H	-1.696469	1.000379	-2.718485
H	-5.253627	0.089703	-1.540552
H	-4.596950	-1.193693	-0.493563
H	-4.019290	0.562762	1.218804
H	-5.092988	2.805978	1.067049
H	-3.961476	2.592665	-0.277577
H	-5.697796	2.429651	-0.555231
H	-6.368510	0.827553	2.030164
H	-6.068832	-0.811730	1.427614
H	-6.975759	0.348169	0.437868
H	4.731599	-1.860041	-2.176070
H	4.731363	-2.766057	-0.646729
H	3.246811	-2.634830	-1.597934
H	5.714885	-0.291535	-0.657784

Conformer 58:

C	-1.553001	-1.353228	0.418794
C	-1.821626	-0.366901	-0.563577
C	-0.703453	0.097520	-1.340034
C	0.627813	-0.360476	-1.152556
C	0.839584	-1.298029	-0.147724
C	-0.226540	-1.814876	0.644656
C	1.697698	0.090400	-2.131547
C	3.059999	0.582117	-1.586874
O	2.065703	-1.831685	0.112624
C	2.926592	1.790342	-0.636568
C	2.662351	1.446632	0.813613
C	2.833214	0.196340	1.269335
C	3.220625	-0.953935	0.389301
C	2.250373	2.615744	1.698376

H	3.618033	0.916535	-2.473480
C	0.017781	-2.789592	1.679307
C	0.754242	2.953140	1.505897
C	2.567913	2.430010	3.189392
C	-3.157924	0.190588	-0.840522
O	-3.294411	1.022591	-1.766505
O	-2.541804	-1.859656	1.154872
O	-0.901033	0.995270	-2.302225
O	-0.882934	-3.280948	2.391366
C	-4.387694	-0.214130	-0.051652
C	-5.665609	0.575061	-0.384600
C	-6.892272	-0.167113	0.169917
C	-5.608141	2.012528	0.157134
C	3.904202	-0.524131	-0.920709
C	4.188571	-1.722842	-1.830878
O	5.161772	0.032255	-0.481136
H	1.879180	-0.726584	-2.841867
H	1.274385	0.902239	-2.724626
H	3.856888	2.373500	-0.661429
H	2.142381	2.460794	-1.010239
H	2.675193	-0.040691	2.317495
H	3.896500	-1.631034	0.918566
H	2.825654	3.485362	1.345306
H	1.056894	-3.105796	1.835293
H	0.494121	3.862467	2.058799
H	0.498787	3.117824	0.454693
H	0.127062	2.136752	1.880696
H	2.344440	3.351414	3.737560
H	3.622990	2.185804	3.348601
H	1.962507	1.633110	3.635933
H	-2.121566	-2.525319	1.790348
H	-1.897906	1.194531	-2.302363
H	-4.538507	-1.287809	-0.227656
H	-4.161069	-0.151410	1.018941
H	-5.758192	0.627993	-1.475963
H	-7.814926	0.374361	-0.066426
H	-6.978120	-1.175168	-0.251951
H	-6.836603	-0.265992	1.261510
H	-6.520881	2.561186	-0.101198
H	-4.759208	2.563486	-0.256554
H	-5.520915	2.012539	1.251665
H	4.654778	-1.386411	-2.765854
H	4.879766	-2.406349	-1.327492
H	3.286370	-2.281578	-2.083523
H	5.745427	0.110627	-1.247645

Conformer 59:

C	-1.842554	-0.660743	0.664266
C	-1.948854	0.362381	-0.313174
C	-0.810008	0.559995	-1.171904
C	0.381122	-0.209856	-1.088219
C	0.441156	-1.176834	-0.091624
C	-0.650015	-1.425704	0.791651
C	1.446325	-0.022627	-2.154164
C	2.926543	0.125847	-1.728365
O	1.522592	-1.987218	0.081103
C	3.168490	1.330417	-0.794081
C	2.925456	1.061635	0.677072
C	2.820544	-0.194771	1.137145
C	2.869348	-1.405178	0.254236
C	2.854875	2.290014	1.574984
H	3.473547	0.316649	-2.663109
C	-0.559172	-2.429914	1.822624
C	2.360381	2.014139	3.001637
C	4.211625	3.030253	1.616705
C	-3.126445	1.227828	-0.493962
O	-3.103575	2.119205	-1.376835
O	-2.855012	-0.916771	1.491822
O	-0.856094	1.496663	-2.113836
O	-1.487422	-2.689930	2.617158
C	-4.395903	1.103497	0.317286

C	-5.346887	-0.022915	-0.179102
C	-6.514148	-0.173414	0.807564
C	-5.857678	0.238256	-1.603718
C	3.531857	-1.151456	-1.110598
C	3.449568	-2.381785	-2.019124
O	4.916180	-0.913950	-0.776763
H	1.369800	-0.856296	-2.864219
H	1.182723	0.869835	-2.723404
H	4.207626	1.660699	-0.909786
H	2.543810	2.173550	-1.118074
H	2.654094	-0.389417	2.192299
H	3.406331	-2.220124	0.747275
H	2.130888	2.971779	1.102055
H	0.383020	-2.986979	1.898900
H	2.247904	2.957975	3.545123
H	1.390172	1.507276	3.005351
H	3.071124	1.396774	3.563006
H	4.131322	3.933018	2.231760
H	4.547430	3.337079	0.621964
H	4.985829	2.388808	2.052842
H	-2.549240	-1.660885	2.106753
H	-1.766007	1.947569	-2.019623
H	-4.157721	0.926790	1.367294
H	-4.906877	2.067446	0.221572
H	-4.784128	-0.963875	-0.175675
H	-7.193690	-0.970897	0.486888
H	-6.156078	-0.418965	1.813012
H	-7.096656	0.754377	0.871597
H	-6.510061	-0.579185	-1.930710
H	-5.041158	0.325364	-2.327196
H	-6.436868	1.168599	-1.648936
H	3.912100	-2.166208	-2.990874
H	3.990928	-3.213217	-1.556582
H	2.422825	-2.704914	-2.196318
H	5.439918	-0.971013	-1.587191

Conformer 60:

C	1.610858	-1.403695	-0.583133
C	1.908998	-0.343468	0.310119
C	0.822282	0.153224	1.111977
C	-0.502365	-0.356791	1.045748
C	-0.742628	-1.375523	0.131092
C	0.288872	-1.918672	-0.689132
C	-1.524061	0.139352	2.054686
C	-2.935437	0.550051	1.574941
O	-1.964684	-1.961907	-0.011801
C	-2.913151	1.693752	0.537106
C	-2.713456	1.250581	-0.895573
C	-2.862838	-0.036473	-1.246658
C	-3.160652	-1.131588	-0.268211
C	-2.392832	2.309018	-1.940512
H	-3.450675	0.925398	2.467930
C	0.011998	-2.970544	-1.635806
C	-3.456405	3.424522	-1.984600
C	-0.982293	2.904411	-1.750549
C	3.245786	0.262491	0.472775
O	3.396816	1.190719	1.299313
O	2.564430	-1.929081	-1.352082
O	1.044873	1.132325	1.984532
O	0.880417	-3.484597	-2.371797
C	4.442178	-0.204515	-0.332245
C	5.791296	0.506183	-0.091526
C	5.779134	1.991162	-0.492509
C	6.348035	0.297150	1.327101
C	-3.778238	-0.626269	1.052867
C	-3.960447	-1.758180	2.068325
O	-5.068643	-0.060065	0.750915
H	-1.630036	-0.623813	2.836610
H	-1.091579	1.007275	2.554047
H	-3.867050	2.232327	0.588963
H	-2.139189	2.418295	0.818245

H	-2.737517	-0.336127	-2.285724
H	-3.839619	-1.867955	-0.713681
H	-2.404705	1.803285	-2.915024
H	-1.023653	-3.327573	-1.699708
H	-3.252268	4.107347	-2.816537
H	-4.461592	3.012368	-2.121867
H	-3.456203	4.019045	-1.064316
H	-0.755620	3.602410	-2.564073
H	-0.216983	2.121932	-1.751458
H	-0.898045	3.458770	-0.809475
H	2.125927	-2.650080	-1.910195
H	2.030301	1.367411	1.899980
H	4.552649	-1.282129	-0.158893
H	4.170427	-0.138747	-1.392871
H	6.482053	-0.001934	-0.780467
H	6.794009	2.403265	-0.455271
H	5.407375	2.122157	-1.516080
H	5.148817	2.580552	0.178090
H	7.372772	0.680208	1.393409
H	6.376416	-0.767659	1.588543
H	5.742445	0.816202	2.074151
H	-4.382478	-1.359140	2.995983
H	-4.656714	-2.508325	1.671387
H	-3.026775	-2.275858	2.292712
H	-5.685964	-0.775139	0.545247

Conformer 61:

C	1.673233	-1.292598	-0.502683
C	1.907469	-0.358057	0.537165
C	0.775793	0.013123	1.344001
C	-0.538605	-0.482031	1.130485
C	-0.717630	-1.361981	0.069061
C	0.364369	-1.789707	-0.754695
C	-1.619760	-0.135640	2.140079
C	-3.005495	0.338686	1.644919
O	-1.925757	-1.918764	-0.227431
C	-2.936218	1.617944	0.783833
C	-2.636116	1.389729	-0.683162
C	-2.750858	0.166292	-1.223555
C	-3.108823	-1.055329	-0.431233
C	-2.246740	2.625055	-1.484330
H	-3.573617	0.581015	2.551758
C	0.153489	-2.708131	-1.846213
C	-1.707987	2.334209	-2.891772
C	-3.416406	3.633263	-1.559507
C	3.222028	0.235130	0.843066
O	3.331566	1.010654	1.820046
O	2.678065	-1.715907	-1.269099
O	0.943660	0.854455	2.360791
O	1.069218	-3.121708	-2.588009
C	4.460894	-0.065383	0.022299
C	5.705195	0.759940	0.392920
C	6.958123	0.110375	-0.216331
C	5.576277	2.223608	-0.058943
C	-3.806973	-0.748748	0.909622
C	-4.038793	-2.016138	1.737317
O	-5.082095	-0.144230	0.616690
H	-1.762492	-1.002830	2.797937
H	-1.223659	0.652428	2.781805
H	-3.898484	2.137775	0.857714
H	-2.186648	2.300102	1.206826
H	-2.541836	0.000106	-2.276185
H	-3.755680	-1.715840	-1.020369
H	-1.438571	3.112587	-0.917124
H	-0.873655	-3.053166	-2.019938
H	-1.363414	3.263318	-3.357480
H	-0.864030	1.637432	-2.868593
H	-2.482956	1.911407	-3.541587
H	-3.104738	4.534974	-2.097563
H	-3.762009	3.941698	-0.568783
H	-4.267408	3.195476	-2.093675

H	2.281499	-2.357116	-1.943249
H	1.931777	1.091767	2.371609
H	4.661677	-1.139654	0.132996
H	4.221516	0.050575	-1.040970
H	5.807191	0.750289	1.484644
H	7.857747	0.678309	0.045338
H	7.094399	-0.916578	0.141880
H	6.894979	0.076016	-1.311453
H	6.465924	2.797063	0.224836
H	4.707960	2.708725	0.395086
H	5.476714	2.286369	-1.150634
H	-4.520177	-1.756067	2.685241
H	-4.701916	-2.702076	1.194283
H	-3.115053	-2.559350	1.941492
H	-5.681090	-0.822421	0.276184

Conformer 64:

C	-1.705640	-1.292547	0.349041
C	-1.849118	-0.546063	-0.848942
C	-0.640410	-0.230567	-1.565174
C	0.656399	-0.617965	-1.131643
C	0.738330	-1.323040	0.063232
C	-0.418901	-1.676473	0.818298
C	1.836102	-0.363985	-2.054631
C	3.137601	0.242413	-1.481585
O	1.918824	-1.773379	0.573011
C	2.922491	1.622681	-0.826302
C	2.484264	1.590746	0.621600
C	2.580424	0.465199	1.346074
C	3.047710	-0.840525	0.775779
C	1.990323	2.915521	1.187119
H	3.800311	0.384511	-2.344381
C	-0.305166	-2.401392	2.059432
C	0.535024	3.194720	0.747188
C	2.120775	3.047744	2.711512
C	-3.137495	-0.111326	-1.414308
O	-3.151077	0.515297	-2.501356
O	-2.774335	-1.635302	1.067477
O	-0.715566	0.443408	-2.708003
O	-1.286254	-2.740736	2.754463
C	-4.473971	-0.357019	-0.751862
C	-4.803828	0.652107	0.384644
C	-4.927540	2.090707	-0.138715
C	-6.090503	0.211409	1.097998
C	3.882699	-0.692436	-0.513451
C	4.245091	-2.051827	-1.118734
O	5.097637	0.005712	-0.176749
H	2.082791	-1.306373	-2.560713
H	1.491883	0.306138	-2.843451
H	3.863681	2.186713	-0.864671
H	2.201919	2.197414	-1.421739
H	2.295247	0.451098	2.394240
H	3.642944	-1.386954	1.516561
H	2.620190	3.695985	0.733498
H	0.705698	-2.662123	2.397214
H	0.226782	4.197078	1.064129
H	0.412362	3.136203	-0.338500
H	-0.148003	2.469326	1.202829
H	1.848497	4.061360	3.024243
H	3.144306	2.851603	3.046579
H	1.451684	2.356614	3.236574
H	-2.434430	-2.147129	1.872087
H	-1.707962	0.615990	-2.870224
H	-5.222772	-0.256753	-1.544712
H	-4.514140	-1.369587	-0.347353
H	-3.987448	0.613893	1.115593
H	-5.142246	2.779709	0.685901
H	-4.012190	2.432482	-0.631712
H	-5.743287	2.174596	-0.867077
H	-6.339337	0.900981	1.912380
H	-5.985058	-0.791533	1.525056

H	-6.939594	0.197068	0.403036
H	4.819527	-1.906057	-2.038874
H	4.866194	-2.621196	-0.415093
H	3.367630	-2.662249	-1.336813
H	5.673020	-0.587300	0.325226

Conformer 69:

C	-1.669509	-1.290691	0.506299
C	-1.906641	-0.363172	-0.539413
C	-0.776959	0.003136	-1.351044
C	0.537604	-0.489472	-1.135806
C	0.720397	-1.361451	-0.068195
C	-0.359784	-1.784878	0.759585
C	1.618465	-0.149464	-2.147113
C	3.000374	0.332438	-1.644764
O	1.929689	-1.914708	0.228412
C	2.918419	1.615472	-0.790552
C	2.627080	1.391450	0.679298
C	2.747956	0.171297	1.225037
C	3.110786	-1.053078	0.439876
C	2.240085	2.630155	1.476903
H	3.564992	0.576923	-2.556251
C	-0.145822	-2.696103	1.857056
C	1.699653	2.343334	2.884618
C	3.412894	3.634801	1.551955
C	-3.222209	0.227304	-0.846477
O	-3.334293	0.995766	-1.828763
O	-2.672798	-1.709842	1.276976
O	-0.947012	0.837039	-2.374102
O	-1.060170	-3.105619	2.602569
C	-4.459197	-0.067280	-0.020633
C	-5.703683	0.757020	-0.393130
C	-6.955688	0.112563	0.223406
C	-5.572097	2.223333	0.049303
C	3.807676	-0.748671	-0.897221
C	4.049076	-2.021632	-1.713912
O	5.084315	-0.204784	-0.498853
H	1.767839	-1.022723	-2.795324
H	1.221189	0.630976	-2.797564
H	3.872488	2.150224	-0.868253
H	2.158802	2.286267	-1.213817
H	2.546187	0.010358	2.279726
H	3.765236	-1.705384	1.024491
H	1.433647	3.119171	0.908479
H	0.881998	-3.038607	2.031510
H	1.355453	3.273944	3.347633
H	0.855180	1.647184	2.862068
H	2.473552	1.921390	3.536107
H	3.104292	4.537431	2.090274
H	3.758745	3.943077	0.561099
H	4.262674	3.193925	2.085359
H	-2.274579	-2.346191	1.954870
H	-1.934976	1.073797	-2.384788
H	-4.661354	-1.141982	-0.124020
H	-4.216955	0.055189	1.041273
H	-5.808550	0.740703	-1.484514
H	-7.855427	0.679722	-0.039546
H	-7.093818	-0.916497	-0.127970
H	-6.889771	0.085045	1.318556
H	-6.461905	2.795909	-0.235729
H	-4.704407	2.704653	-0.409940
H	-5.469643	2.292761	1.140317
H	4.527747	-1.773118	-2.669849
H	4.714731	-2.690069	-1.158560
H	3.127231	-2.564557	-1.926900
H	5.668310	-0.200798	-1.269101

2. References

(1) Liu, H.; He, X. Z.; Feng, M. Y.; Yuan-Zeng; Rauwolf, T. J.; Shao, L. D.; Ni, W.; Yan, H.; Porco, J. A.; Hao, X. J.; Qin, X. J.; Liu, H. Y. Acylphloroglucinols with Acetylcholinesterase Inhibitory Effects from the Fruits of *Eucalyptus robusta*. *Bioorg Chem* **2020**, *103*. https://doi.org/10.1016/j.bioorg.2020.104127.

(2) Daus, M.; Wunnoo, S.; Voravuthikunchai, S. P.; Saithong, S.; Poldorn, P.; Jungsuttiwong, S.; Chomlamay, N.; Yangok, K.; Watanapokasin, R.; Chakthong, S. Phloroglucinol–Meroterpenoids from the Leaves of *Eucalyptus camaldulensis* Dehnh. *Phytochemistry* **2022**, *200*. https://doi.org/10.1016/j.phytochem.2022.113179.

(3) Baxter, J. R.; Holland, D. C.; Gavranich, B.; Nicolle, D.; Hayton, J. B.; Avery, V. M.; Carroll, A. R. NMR Fingerprints of Formyl Phloroglucinol Meroterpenoids and Their Application to the Investigation of *Eucalyptus gittinsii* subsp. *gittinsii*. *J Nat Prod* **2023**, *86* (5), 1317–1334. https://doi.org/10.1021/acs.jnatprod.3c00139.

(4) Grimblat, N.; Zanardi, M. M.; Sarotti, A. M. Beyond DP4: An Improved Probability for the Stereochemical Assignment of Isomeric Compounds Using Quantum Chemical Calculations of NMR Shifts. *Journal of Organic Chemistry* **2015**, *80* (24), 12526–12534. https://doi.org/10.1021/acs.joc.5b02396.
